# Revived ancient viruses from deep-sea ecosystems are biothreats by triggering gut dysbiosis

**DOI:** 10.1128/mbio.01217-25

**Published:** 2025-07-11

**Authors:** Rui Wang, Mengqi Chu, Xiaobo Zhang

**Affiliations:** 1 College of Life Sciences, Laboratory for Marine Biology and Biotechnology of Qingdao Marine Science and Technology Center and Southern Marine Science and Engineering Guangdong Laboratory (Zhuhai), Zhejiang University 98445 , Hangzhou, People’s Republic of China; The University of Tennessee Knoxville, Knoxville, Tennessee, USA; Universita degli Studi di Padova, Padova, Italy

**Keywords:** ancient virus, deep-sea sediment, gut microbiota, mouse, health

## Abstract

As human activities increase in the deep sea, ancient viruses trapped in sediments may revive in the human gut by infecting gut bacteria. Nevertheless, the potential biosecurity risks that deep-sea viruses pose to humans remain unexplored. Here, two bacteriophages DP105 and DP016, purified from the ancient deep-sea sediments, proliferated in the gut of mice to trigger the intestinal inflammation of mice. The biothreats posed by deep-sea viruses to mouse gut microbiota were further evaluated globally using the viruses purified from 106 ancient deep-sea sediment samples. The viruses purified from nine of these sediments could revive in the mouse gut, leading to disrupting gut bacterial communities and causing inflammatory symptoms, liver damage, or irregular glucose metabolism. Viruses with biosecurity risks were found in the seafloors within three distinct oceans.

**IMPORTANCE:**

Our findings highlighted the biothreats of ancient deep-sea viruses to mammal health. The biosecurity should be evaluated before exploring the deep-sea resources.

## INTRODUCTION

In the last few decades, the global spread of various infectious diseases has occurred frequently, and the emergence of new pathogens has seriously jeopardized human health and posed great challenges to public health (1, 2). The outbreaks caused by viruses continue to cause global concern, such as the recurring outbreaks of Ebola virus (3), human immunodeficiency virus (4), and severe acute respiratory syndrome coronavirus 2 (5). Virus traceability is particularly important for the prevention of infectious diseases, which is conducive to exploring the origin of pathogens and preventing infectious disease epidemics. According to some reports, the viruses may originate directly or indirectly from wildlife and then transmit to humans, e.g., dengue virus spreads rapidly in populations through mosquitoes (6). Severe acute respiratory syndrome coronavirus and Middle East respiratory syndrome coronavirus are possibly from bats (7). In addition, climate change alters ecosystem environments, with complex effects on the range of infectious diseases, resulting in dynamic changes in disease prevalence (8, 9). Therefore, the virus traceability requires more comprehensive investigation and analysis, and the potential pathogens in the environment should be characterized in detail. In recent decades, the origin tracing of the infectious viruses has been well characterized on the land (10). Nevertheless, the origins of viruses are largely unknown (7, 11, 12). As well known, the ecosystems of the earth consist of the sunlight-dependent ecosystem, including the land and the marine surface, and the sunlight-independent deep-sea ecosystem (13–16). At present, however, the origin tracing of human pathogenic viruses in the deep sea remains to be explored.

The deep sea, the largest ecosystem and usually defined as an extreme environment, differs from the terrestrial and offshore environments (17, 18). Despite low temperature, high pressure, and no light, the deep sea harbors a lot of biological entities including viruses (14, 15). Some eukaryotic viruses can move between marine and terrestrial hosts, thus potentially remaining infectious for a long time and becoming a source of new pathogens (19–21). The marine viruses belonging to *Caliciviridae* are zoonotic and may cause human disease (22). At present, however, no eukaryotic virus in the deep sea is infectious to humans. Except for eukaryotic viruses, the deep sea is rich in prokaryotic viruses, especially bacteriophages (phages), the viruses that infect bacteria (14, 15, 23). Bacteriophages, widely found in animal and human guts, help shape gut microbiota, regulate mucosal immunity, and influence inflammation, which are closely related to human health (24–27). Due to the more and more frequent human activities, such as scientific investigations and resource exploitation, in the deep sea (28, 29), a lot of deep-sea samples containing viruses come into the land, thus causing the biothreats of deep-sea bacteriophages to invade human gut microbiota. However, the risks of deep-sea viruses to human health remain unclear.

To investigate whether deep-sea viruses could impact human health by affecting the gut microbiota, the viruses purified from each of 106 deep-sea sediment samples worldwide were tested in mice for their effects on health. The results indicated that some deep-sea viruses caused health damage in mice by altering gut microbiota.

## MATERIALS AND METHODS

### Deep-sea sediment sample collection and conventional radiocarbon age determination

A total of 106 sediment samples were collected from the Pacific, Atlantic, and Indian Oceans during the 22nd, 26th, 30th, 34th, 39th, 40th, and 45th geomicrobiology cruises of oceanic vessel No.1 (Dayang No. 1) (Table S1). Samples from ecosystems such as hydrothermal vents, cold seeps, ocean basins, hadal trenches, and mid-ocean ridges were sealed on-site and stored at −20°C. Conventional radiocarbon age of deep-sea sediments was determined by Beta Analytical Testing Laboratories (USA) as described previously (30).

### Isolation of deep-sea bacteria and bacteriophages

The sediment sample was incubated with 1 mL Luria-Bertani (LB) medium at 37°C for 1 hour, and the supernatant was spread on an LB agar plate. The single bacterial colonies were purified three times. Subsequently, the strains were identified by 16S rRNA gene sequencing.

Mitomycin (500 ng/mL) was added to the bacterial culture (OD_600_ = 0.3–0.4), followed by incubation at 37°C for 6 hours. The bacterial culture was then centrifuged at 10,000 × *g* for 30 minutes, and the supernatant was collected and mixed with 10% (wt/vol) PEG8000, incubating overnight at 4°C. Subsequently, the mixture was centrifuged at 40,000 × *g* for 3 hours and resuspended in SM buffer (10 mM Tris-HCl, 100 mM NaCl, 10 mM MgSO_4_, pH 7.5). An equal volume of chloroform was added and then centrifuged to collect the supernatant. The bacteriophages were purified using CsCl density gradient (1.3, 1.45, 1.5, and 1.7 g/mL) centrifugation (200,000 × *g*, 6 hours), followed by ultrafiltration (10 kDa cutoff). The purified bacteriophages were resuspended in saline magnesium buffer (SM buffer; 50 mM Tris [pH 7.5], 100 mM NaCl, 10 mM MgSO_4_) and stored at 4°C.

### Purification of deep-sea viruses

Glass beads were added to 5 g of sediment samples, along with 5 mL of SM buffer, and shaken for 30 minutes. The mixture was centrifuged at 5,000 × *g* for 7 minutes, and the supernatant was collected. This step was repeated eight to nine times. The combined supernatants (40 mL) were centrifuged at 5,000 × *g* for 20 minutes. PEG6000 (4 g) was then added, and the mixture was incubated at 4°C overnight. Subsequently, the sample was centrifuged at 100,000 × *g* for 2 hours, and the viruses were collected.

### Treatment of mice with deep-sea viruses and sample collection

Eight-week-old Institute of Cancer Research (ICR) mice were divided into experimental and control groups, with three males and three females in each group. Before the experiment, the mice were provided with sterilized water for 3 days in a specific pathogen-free environment.

Treatment of mice with purified bacteriophage. The mice were gavaged or clystered with the bacteriophage DP105 or DP016, with SM buffer serving as the control. Gavage was performed daily, while clyster was carried out once every 2 days. After 6 days, colon tissues from the mice were collected.

Treatment of mice with viruses from deep-sea sediments. The mice in the experimental group were gavaged daily with viruses purified from 106 deep-sea sediment samples for 3 days, followed by regular feeding for 4 days. Body weights were measured, and fecal samples were collected on days 3 and 10. On day 10, their blood and intestinal tissues were collected.

### Quantitative real-time PCR

Total RNAs from tissues were extracted using RNA isolation kit (Ambion, USA), and cDNA was synthesized using the PrimeScript RT reagent kit (TaKaRa, Japan). Quantitative real-time PCR was performed with specific primers (Table S4), and the relative mRNA expression levels were calculated using the 2^(−△△Ct)^ method.

### Sequencing of bacterial 16S rRNA and diversity analysis

Bacterial DNA was extracted using the FastDNA SPIN kit (MP Biomedicals, USA) for 16S rRNA sequencing. The sequence-specific primers (V4-V5, 515F, 5′-GTGCCAGCMGCCGCGG-3′; 907R, 5′-CCGTCAATTCMTTTRAGTTT-3′, M = A/C; R = A/G) were used for amplification. The alpha diversity, including the Chao, Simpson, and Shannon indices, was evaluated using the online Mothur software (http://www.mothur.org/wiki). The beta diversity was analyzed by generating principal co-ordinate analysis (PCoA) plots at the operational taxonomic unit (OTU) level using the weighted UniFrac distance. The vegan package in R (version 3.4.4) (https://www.r-project.org, Mingkebio, China) was used.

### Hematological analysis

Hematological analysis of mouse whole blood samples was performed using the Sysmex xt-2000i whole blood cell analyzer (Sysmex, Japan). Alanine aminotransferase (ALT) and glucose levels in serum samples were measured with the Roche Cobas c311 fully automatic biochemical analyzer (Roche, Switzerland).

### Examination of tuft cells

Fresh mouse intestinal tissues were immersed in 4% paraformaldehyde, embedded in paraffin, and sectioned. The sections were sequentially washed with xylene, anhydrous ethanol, 85% ethanol, and 75% ethanol, then immersed in EDTA antigen retrieval buffer (Wuhan ServiceBio Technology, China) and heated. The sections were incubated overnight at 4°C with double cortin-like kinase 1 (DCLK1) (Abcam, UK) antibody as a marker for tuft cells. Subsequently, they were incubated at room temperature for 50 minutes with a fluorescently labeled secondary antibody (Abcam) and counterstained with 4′,6-diamidino-2-phenylindole (DAPI) for nuclei staining.

### Correlation analysis

The online OmicStudio software (https://www.omicstudio.cn) was applied for correlation analysis. Based on Spearman’s rank correlation coefficient using the vegan package in R (Version 3.6.1), the correlation analysis was performed.

### Statistical analysis

The experiments were biologically repeated for at least three times. The numerical data were characterized with one-way analysis of variance and shown as mean ± standard deviation. The intergroup significant difference analysis was conducted using a two-tailed Student’s *t*-test. The power analysis was performed using G*Power to evaluate the achieved power after data collection, with an alpha level of 0.05, which confirmed that the statistical power was sufficient (power > 0.8).

## RESULTS

### Viral composition of deep-sea sediments

**Fig 1:**
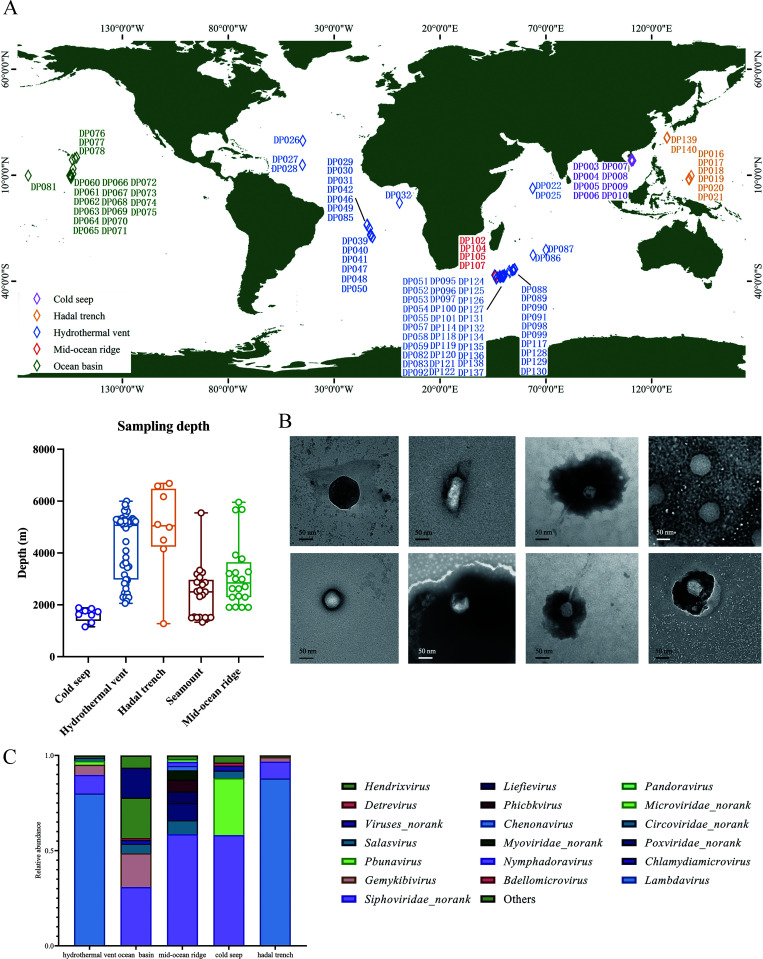
Viral composition of deep-sea sediments. (**A**) The distribution and sampling depth of deep-sea sediment samples. (**B**) Representative images of the viruses purified from deep-sea sediments. The viruses were observed with transmission electron microscopy. Scale bar, 50 nm. (**C**) The dominant families in the samples from different geographical environments. Only the families with the abundance above 1% in each geographical environment were indicated. The remaining families were classified as “others.”

To explore the impact of deep-sea viruses on the gut microbiota and health of mice on a global scale, a total of 106 deep-sea sediment samples from different deep-sea locations were used to purify viruses, with sampling depths ranging from 1,154 to 6,682 m (Fig. 1A; Table S1). The viruses could be observed using a transmission electron microscope (Fig. 1B).

Based on the previous virome data set of deep-sea sediments (National Omics Data Encyclopedia database accession number OEP002479) revealed by our laboratory, most of the viruses from 106 deep-sea sediments were unclassified (14). Among the classified viruses, 25.75% were prokaryote viruses and 44.55% were eukaryote viruses. The classified viruses belonged to 12 families and 102 species. At the family level, the samples from ocean basins contained the viruses of *Circoviridae*, *Genomoviridae*, *Siphoviridae,* and *Phycodnaviridae* (Fig. 1C). The samples from mid-ocean ridges mainly contained *Siphoviridae*, *Myoviridae*, *Circoviridae*, *Asfarviridae*, and *Phycodnaviridae*. The most abundant family in the samples from hadal trenches was *Siphoviridae*, followed by *Genomoviridae*, *Circoviridae*, *Myoviridae*, *Podoviridae*, and *Herelleviridae*. The samples from cold seeps were dominated by *Circoviridae*, *Siphoviridae*, *Microviridae,* and *Myoviridae. Circoviridae*, *Myoviridae*, *Siphoviridae*, *Genomoviridae*, *Microviridae*, *Podoviridae*, and *Phycodnaviridae* were significantly accumulated in the samples from hydrothermal vents (Fig. 1C).

Collectively, these results demonstrated that there existed numerous viruses in the bottoms of global oceans.

### Gut microbiota dysbiosis of mice induced by deep-sea viruses

**Fig 2:**
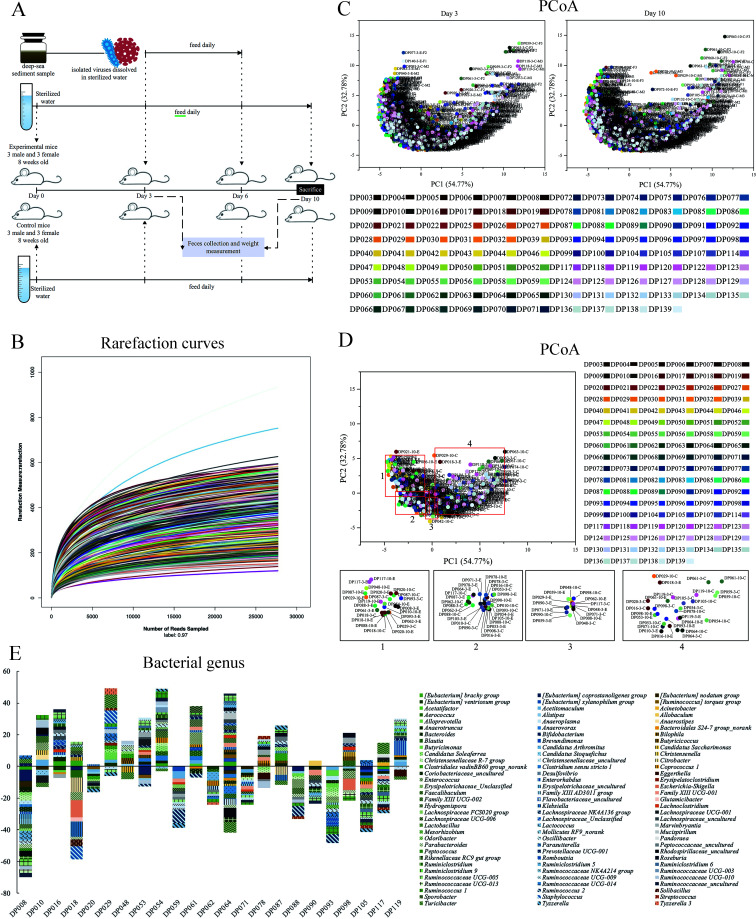
Gut microbiota dysbiosis of mice induced by deep-sea viruses. (**A**) A flow diagram of the experiment. Six male and six female ICR mice (8 week old) were randomly divided into two groups (experimental mice and control mice). Each group contained three female and three male mice. Both groups of mice were fed with sterilized water in a sterile environment for 3 days, followed by feeding with the viruses purified from deep-sea sediments or sterilized water daily for a 3 day period. Four days later, all the mice were sacrificed. Body weight measurement and fecal sample collection of mice were conducted at days 3 and 10. (**B**) Rarefaction curves of the 16S rRNA genes for gut microbiota in all mice. (**C**) PCoA of the mouse gut bacterial communities of 106 experiment groups and control groups at day 3 (left) and day 10 (right). For each group, three female and three male mice were used. PCoA analysis was conducted based on weighted UniFrac distance. (**D**) PCoA analysis of the bacterial communities of 106 groups of mice treated with the viruses isolated from deep-sea sediments. The mice were fed with the viruses from one of 106 deep-sea sediment samples, and then the fecal samples were collected for the analysis of gut microbiota. Each dot represented a group of mice at day 3 or 10. The enlarged images below showed the samples with altered beta diversity of gut microbiota (*P* < 0.05). “E” indicated the fecal samples of experiment mice, while “C” represented the fecal samples of control mice. (**E**) Significantly increased or decreased bacterial genus in the mice treated with the viruses purified from 23 deep-sea sediments. The significant difference in bacterial abundance at the genus level between different treatments was analyzed using a two-tailed Student’s *t*-test.

To investigate the effects of deep-sea viruses on a global scale on the gut microbiota of mice, ICR mice were fed with purified viruses from 106 deep-sea sediment samples or sterile water daily for three consecutive days (Fig. 2A). Gut microbiota analysis of mouse feces was conducted before virus feeding and 4 days after returning to normal feeding (Fig. 2A). A total of 12,225,944 clean reads and 3,933 OTUs were determined based on the 16S rRNA gene sequence (Table S2). The rarefaction curves showed that all samples reached the plateaus on the basis of OTUs with 97% similarity (Fig. 2B). The sequencing coverage of the samples ranged from 98.86% to 99.91% (Table S2). The analysis of Shannon, Simpson, and Chao indices revealed that the mouse gut microbiota exhibited high diversity (Table S2). The PCoA results showed that the gut microbiota was similar between male and female mice in each group (Fig. 2C), showing that the statistical errors between repeated experiments were smaller and the data of repeated experiments were reliable. Meanwhile, no significant changes in the gut microbiota of control groups were observed at different sampling times (Fig. 2C), indicating that the mouse gut microbiota is stable.

Further analysis revealed that the gut microbiota structure (beta diversity) of mice underwent changes after treatment with the purified viruses from 23 deep-sea sediment samples, including DP008, DP010, DP016, DP018, DP020, DP029, DP048, DP053, DP054, DP059, DP061, DP062, DP064, DP071, DP078, DP087, DP088, DP090, DP093, DP098, DP105, DP117, and DP119 (Fig. 2D). The gut microbiota abundance of the mice treated with the viruses from these 23 deep-sea sediment samples showed significant changes in the abundance of bacteria belonging to 26 genera (Fig. 2E; Table S3).

Taken together, the viruses from 23 deep-sea sediments proliferated in the mouse gut microbiota, resulting in changes in gut bacterial community.

### Impact of deep-sea viruses on mouse health

**Fig 3:**
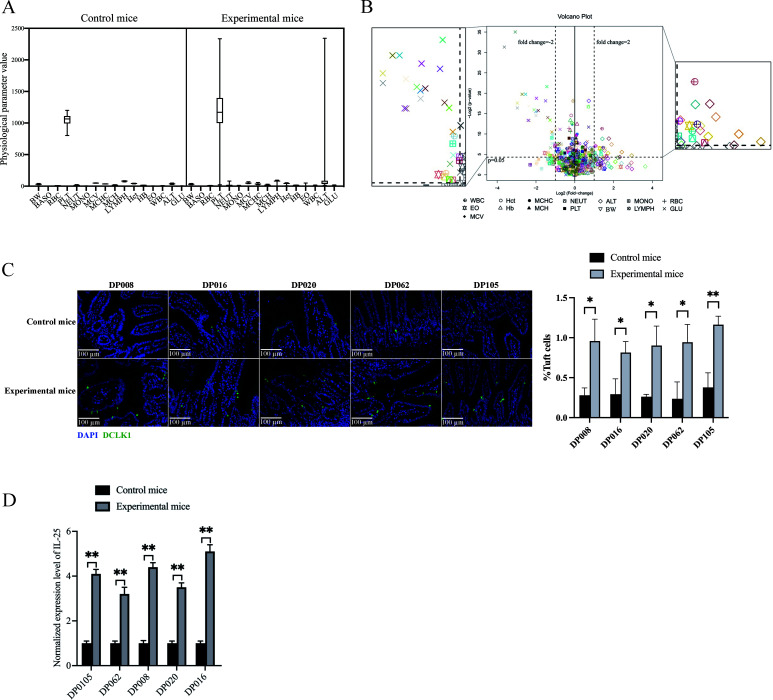
Impact of deep-sea viruses on mouse health. (**A**) Box plots of the values of 16 physiological parameters of mice. The experimental mice were fed with the viruses purified from one of 106 deep-sea sediment samples, while the control mice were fed with sterilized water. At day 10 after feeding, the physiological parameters of mice were examined. Body weight, BW; basophilic granulocyte, BASO; red blood cell, RBC; platelet, PLT; neutrophil, NEUT; monocyte, MONO; mean corpuscular volume, MCV; mean corpuscular hemoglobin concentration, MCHC; mean corpuscular hemoglobin, MCH; lymphocyte, LYMPH; hematocrit, Hct; hemoglobin, Hb; eosinophil, EO; white blood cell, WBC; alanine aminotransferase, ALT; blood glucose, GLU. (**B**) Influence of deep-sea viruses on mouse health. The experimental mice and the control mice were subjected to the examination of 16 physiological parameters. The differences in physiological parameters between the experimental mice and the control mice were shown with volcano plots. *X* and *Y* axes indicated the fold change and the statistical significance of 16 physiological parameters of each mouse, respectively. The enlarged image showed physiological parameters with changes exceeding twofold. (**C**) Impact of deep-sea viruses on tuft cells of mouse small intestine tissues. The small intestines of the mice treated with deep-sea viruses or the control mice were labeled with the antibody against DCLK1 (green), a marker protein of tuft cells. The nuclei were stained with DAPI (blue). The significant differences between treatments were indicated with asterisks (*P < 0.05; **P < 0.01). Scale bar, 100 µm. (**D**) The expression level of IL-25 secreted by tuft cells in the small intestine tissues of mice. The expression level of IL-25 in the control mice and the experimental mice was detected using quantitative real-time PCR (**P < 0.01). In all panels, bar graphs were shown as mean ± standard deviation. The intergroup significant difference was analyzed using a two-tailed Student’s *t*-test.

To evaluate whether deep-sea viruses affect mouse health, the mice were treated with the purified viruses from 106 deep-sea sediment samples, while the control group was given sterile water (Fig. 2A), followed by the detection of 16 physiological parameters associated with inflammation, obesity, and/or liver disease. The physiological parameters data were reliable, with no significant differences observed between the six mice of an experimental group or its corresponding control group (Fig. 3A).

It was found that nine physiological parameters did not exhibit significant difference between control and experimental mice for the purified viruses from 106 deep-sea sediments, including mean corpuscular hemoglobin (MCH) concentration, mean corpuscular hemoglobin, mean corpuscular volume, hematocrit (Hct), hemoglobin (Hb), and the numbers of basophilic granulocyte (BASO), red blood cell, platelet (PLT), and lymphocyte (LYMPH) (Fig. 3A). The number of BASO did not change in the mice treated with the deep-sea viruses compared with the control mice (Fig. 3A), showing that the deep-sea viruses did not induce an allergic response.

The blood glucose (GLU) levels in mice significantly decreased after treatment with the viruses from 20 sediment samples (DP018, DP048, DP051, DP052, DP059, DP060, DP066, DP073, DP077, DP091, DP094, DP099, DP101, DP102, DP104, DP105, DP118, DP119, DP123, and DP134) (Fig. 3B; Table S5). Among the 20 sediment samples, the viruses from five sediments (DP018, DP048, DP059, DP105, and DP119) significantly decreased the mouse body weight (Fig. 3B; Table S5), suggesting that these deep-sea viruses affected the mouse glucose metabolism. The viruses purified from 12 deep-sea sediments (DP016, DP020, DP055, DP068, DP071, DP072, DP074, DP092, DP100, DP105, DP123, and DP128) caused a significant increase in ALT levels in mice (Fig. 3B; Table S5). The viruses from two of the 12 sediment samples (DP016 and DP020) caused significant weight loss in mice (Fig. 3B; Table S5). These data suggested that these deep-sea viruses might harm the mouse liver by disrupting the gut microbiota in mice.

The white blood cell count in mice was significantly increased after intragastric administration of the purified viruses of four deep-sea sediments (DP016, DP020, DP062, and DP105) compared with the control mice (Fig. 3B). The mice treated with the viruses from sample DP020 showed a significant increase in the proportion of monocytes (MONO) (Fig. 3B). The viruses from DP008 or DP054 caused a significant increase in the proportion of neutrophils (NEUT) in mice (Fig. 3B). At the same time, the treatment of the viruses from DP008, DP016, DP020, DP062, or DP105 significantly decreased the mouse weight (Fig. 3B). These data indicated that the viruses from the deep-sea sediment samples DP008, DP016, DP020, DP062, and DP105 might trigger inflammatory responses in mice.

To further determine whether deep-sea viruses caused inflammation, the number of tuft cells, critical cells in gut inflammation (31), and the level of IL-25, a cytokine produced by tuft cells, were measured in the small intestines of mice exposed to the viruses from samples DP008, DP016, DP020, DP062, or DP105. Compared with the control mice, the quantity of tuft cells in the tissue of the small intestine showed a significant increase (Fig. 3C), suggesting that the deep-sea virus triggered the inflammation of mice. Meanwhile, the relative expression level of IL-25 was significantly increased in the experimental mice compared to the control mice (Fig. 3D). These results indicated that the viruses from five deep-sea sediment samples (DP008, DP016, DP020, DP062, and DP105) could provoke inflammatory symptoms of mouse gut.

Taken together, the viruses from deep-sea sediments DP008, DP016, DP018, DP020, DP048, DP059, DP062, DP105, and DP119 could trigger damage to mouse health, including inflammatory symptoms, liver damage, or glucose metabolism abnormalities.

### Damage to mouse health by deep-sea viruses through the gut microbiota

**Fig 4:**
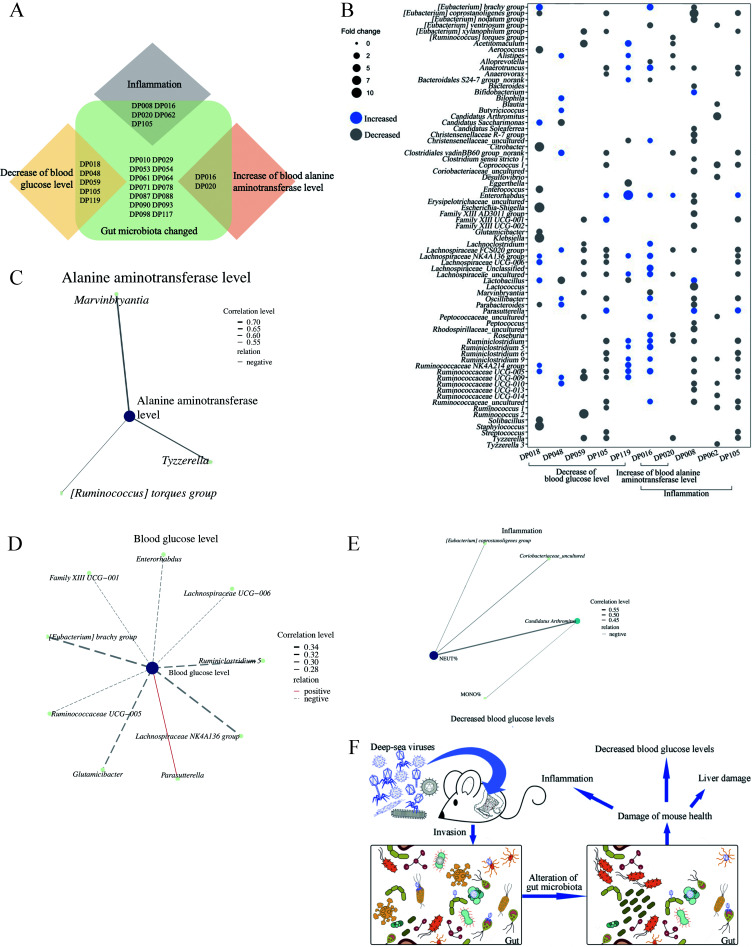
Damage to mouse health by deep-sea viruses through gut microbiota. (**A**) The deep-sea sediments affecting mouse health. The Venn diagram showed the deep-sea sediment samples from which the viruses could alter the gut microbiota to destroy mouse health. (**B**) Influence of deep-sea viruses on mouse gut microbiota and mouse health. The increased and decreased gut bacteria, after treatment of the viruses from deep-sea sediment, led to the decrease of the blood glucose level, the increase of the ALT level of mice, and/or the intestinal inflammation of mice. The increase or decrease level of bacteria was shown as log2(fold change) value. Only the bacteria with log2(fold change) ≥ 1 (fold change ≥ 2) were indicated. (**C**) Correlation analysis of the gut bacteria and the ALT level of the mice treated with deep-sea viruses. The correlation analysis was conducted using Spearman’s rank correlation coefficient. The gut bacteria with significant changes in relative abundance of the mice treated with deep-sea viruses were analyzed. (**D**) The relationship between the gut bacteria and the blood glucose level of mice. The correlation analysis was conducted using Spearman’s rank correlation coefficient. (**E**) Correlation analysis of the gut bacteria and inflammation of mice. The correlation level between bacteria and the percentage of neutrophil (NEUT) or monocyte (MONO) of mice was represented using the line width. (**F**) Model for the invasion of deep-sea viruses into mouse gut microbiota. The deep-sea viruses invaded the gut microbiota of mice, resulting in the alteration of mouse gut bacterial community and then the damage of mouse health. In all panels, correlation analysis was conducted using Spearman’s rank correlation coefficient.

To investigate how deep-sea viruses impaired mouse health, the relationship between gut microbiota and health status in the mice treated with deep-sea viruses was further evaluated. The results indicated that the viruses from nine deep-sea sediment samples (DP008, DP016, DP018, DP020, DP048, DP059, DP062, DP105, and DP119) damaged mouse health and significantly altered their gut microbiota compared to controls (Fig. 4A). The viruses from deep-sea sediment samples DP018, DP048, DP059, DP105, and DP119 caused a significant reduction in blood glucose levels, while the viruses from DP016 and DP020 led to a significant increase in ALT level in mouse blood (Fig. 4A). The treatment with viruses from DP008, DP016, DP020, DP062, and DP105 resulted in intestinal inflammation (Fig. 4A).

The results showed that the viruses from DP018, DP048, DP059, DP105, and DP119 significantly altered the abundance of 26 bacterial genera in the gut microbiota of mice, leading to a significant increase in blood ALT level (Fig. 4B). Based on the correlation analysis, the abundances of the genera *Marvinbryantia*, *Tyzzerella,* and [*Ruminococcus*] torque group were negatively correlated with the ALT levels in mice (Fig. 4C), suggesting that the deep-sea viruses might reduce the contents of gut bacteria from these three genera, leading to liver damage in mice.

The viruses from DP018, DP048, DP059, DP105, and DP119 significantly altered the abundance of 49 bacterial genera of mouse gut microbiota, leading to the decrease in mouse blood glucose level (Fig. 4B). Among the 49 bacterial genera with changes in abundance, eight were negatively correlated with the blood glucose levels in mice, including *Enterorhabdus,* Family XIII UCG-001, *Glutamicibacter, Lachnospiraceae* UCG-006*, Ruminococcaceae* UCG-005*, Lachnospiraceae* NK4A136 group*, Ruminiclostridium* 5*,* and [*Eubacterium*] brachy group, while *Parasutterella* showed a positive correlation with the blood glucose levels in mice (Fig. 4D). As shown in Fig. 4B, the contents of eight bacteria that negatively correlated with the blood glucose level and the abundance of *Parasutterella* were significantly increased and decreased in the deep-sea-virus-treated mice, respectively. In this context, deep-sea viruses from DP018, DP048, DP059, DP105, and DP119 could lower the blood glucose level of mice by altering their gut bacterial community.

In the mice treated with the viruses purified from DP008, DP016, DP020, DP062, or DP105, the abundances of 59 bacterial genera of mouse gut microbiota were significantly altered, resulting in intestinal inflammation (Fig. 4B). The correlation analysis demonstrated that two bacterial genera (*Coriobacteriaceae* uncultured and [*Eubacterium*] *coprostanoligenes* group) were negatively correlated with the percentage of neutrophil (NEUT) of mice, while *Candidatus* Arthromitus was negatively correlated with the proportions of monocyte (MONO) and NEUT of mice (Fig. 4E). These data indicated that the deep-sea viruses from DP008, DP016, DP020, DP062, or DP105 could decrease the contents of 59 bacteria of mouse gut microbiota, thus leading to inflammation in mice.

Collectively, these results presented that the viruses changed the composition of gut microbiota, thus damaging the mouse health, including the decreased blood glucose level, liver damage, and inflammation (Fig. 4F).

### Mouse intestinal inflammation induced by deep-sea viruses

**Fig 5:**
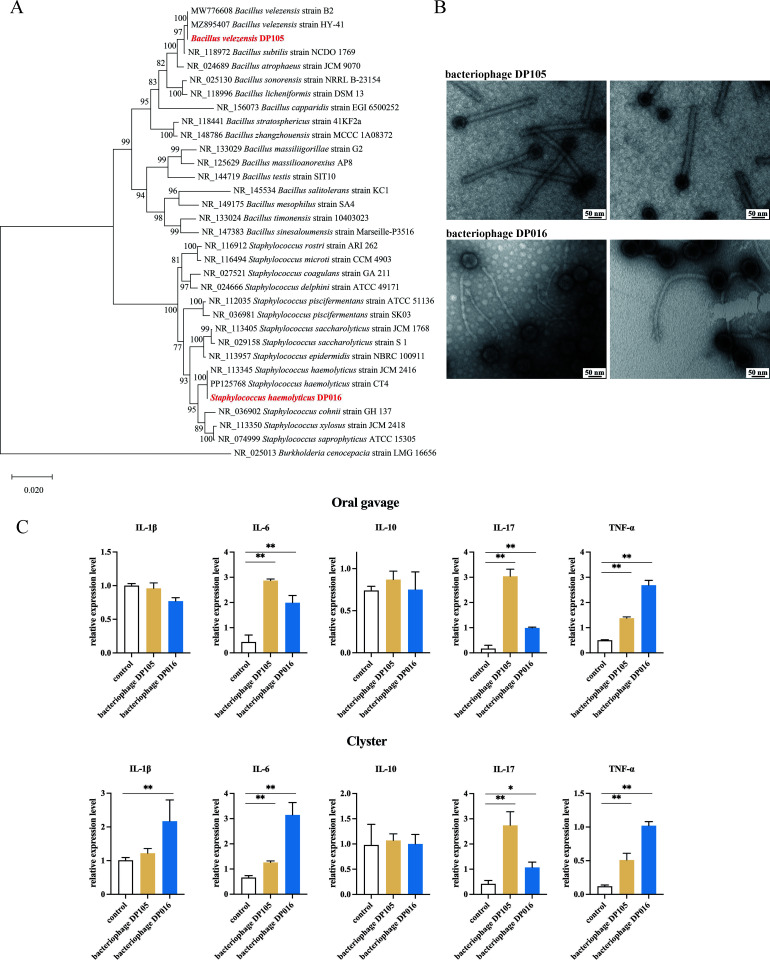
Mouse intestinal inflammation induced by deep-sea bacteriophages. (**A**) The bacterial strains isolated from deep-sea sediments. After screening for bacteria from deep-sea sediments, the strains belonging to *B. velezensis* and *S. haemolyticus* were obtained from DP105 and DP016, respectively. The phylogenetic tree was constructed based on the bacterial 16S rRNA gene sequences by the Neighbor-Joining method and visualized by MEGA software. (**B**) Bacteriophages purified from *B. velezensis* DP105 (DP105 bacteriophage) or *S. haemolyticus* DP016 (DP016 bacteriophage). Scale bar, 50 nm. (**C**) Influence of deep-sea bacteriophages on mouse health. Mice were treated with deep-sea bacteriophage DP105 or bacteriophage DP016 by oral gavage (*n* = 6) or clyster (*n* = 6). The mice treated with SM buffer (*n* = 6) were used as controls. Oral gavage was performed every day, while clyster was conducted once every 2 days. Six days later, the colons of mice were subjected to quantitative real-time PCR to detect the cytokines (*P < 0.05; **P < 0.01).

To evaluate the influence of deep-sea bacteriophages, the viruses infecting bacteria, on the gut bacterial community and mouse health, the bacteriophages purified from DP016 and DP105, the deep-sea sediments rich in bacteriophages (14), were characterized. Based on the 16S rRNA gene sequences, two bacterial strains homologous with *Bacillus velezensis* and *Staphylococcus haemolyticus* were isolated from DP105 (named as *B. velezensis* DP105) and DP016 (named as *S. haemolyticus* DP016), respectively (Fig. 5A). After screening for bacteriophages, two types of bacteriophages were purified from *B. velezensis* DP105 (named as bacteriophage DP105) and *S. haemolyticus* DP016 (named as bacteriophage DP016), respectively (Fig. 5B). Bacteriophage DP105 and bacteriophage DP016 shared typical morphology of *Myoviridae* and *Siphoviridae*, respectively (Fig. 5B). The sediments DP016 and DP105 were 3369–3330 and 2862–2824 years old, showing that bacteriophage DP016 and bacteriophage DP105 were ancient bacteriophages.

To investigate the impact of deep-sea bacteriophages on mouse health, mice were treated with bacteriophage DP105 or bacteriophage DP016 by oral gavage or clyster, and then the expression levels of cytokines (interleukin-1 beta [IL-1β], IL-6, IL-10, IL-17, and tumor necrosis factor alpha [TNF-α]) associated with intestinal inflammation in the colon tissues of mice were examined. The results showed that both bacteriophages significantly upregulated TNF-α, IL-6, and IL-17 in the colon of mice compared with the control (Fig. 5C), indicating that the deep-sea bacteriophages could induce intestinal inflammation in mice.

Taken together, these findings presented that the bacteriophages from the ancient deep-sea sediments could proliferate in the gut of mice to trigger intestinal inflammation.

### Distribution pattern of the deep-sea sediments containing the viruses damaging mouse health

**Fig 6:**
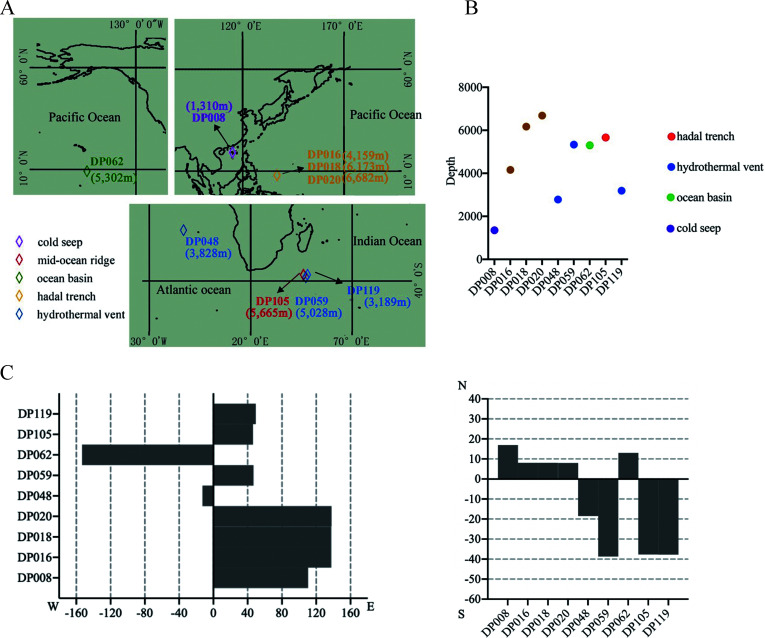
Distribution pattern of the deep-sea sediments containing the viruses destroying mouse health. (**A**) Distribution of deep-sea sediments containing viruses harmful to mouse health. The sampling environments were indicated with different colors. (**B**) Sampling depth of the deep-sea sediments containing the viruses destroying mouse health. (**C**) Distribution of the longitude and latitude of the deep-sea sediments with biosecurity risk.

To characterize the global distribution of the deep-sea sediments with potential biosafety risks, sampling points of sediments containing viruses harmful to mouse health were analyzed. Based on the research results of 106 sediment samples, nine of them (DP048, DP018, DP059, DP105, DP119, DP016, DP020, DP008, and DP062) were found to contain viruses that could alter the gut microbiota of mice and cause health damage. These sediments came from three oceans and five ecosystems (ocean basins, cold seeps, hydrothermal vents, hadal trenches, and mid-ocean ridges) (Fig. 6A), suggesting that the viruses with biosecurity risks were present in different deep-sea ecosystems of oceans.

The sampling depth of the nine sediment samples ranged from 1,350 to 6,173 m (Fig. 6B). Among them, half of the samples (DP018, DP020, DP062, DP059, and DP105) were distributed between 5,300 and 6,700 m (Fig. 6B). The sampling locations of the nine samples ranged from 150°W to 130°E and 20°N to 40°S (Fig. 6C).

Taken together, these results exhibited that the viruses harmful to mouse health were mainly distributed in the bottom of different deep-sea ecosystems of oceans between 10°N and 40°S.

## DISCUSSION

Infectious viruses, one of the most important human pathogens, are great threats to human health (2). Due to the lack of efficient strategies to cure viral diseases, the prevention of virus infection remains the mainstay, thus making viral traceability important (32–35). Although a great deal of investigations of pathogenic virus tracing have been carried out in the land, the origins of viruses are still largely unknown (10, 36–38). The deep sea, a distinct ecosystem on the earth, is rich in viruses (14, 15). However, the biothreat of deep-sea viruses remains unknown. In this study, the data presented that the viruses from nine of 106 deep-sea sediments on a global scale were able to impair mouse health, such as reducing the blood glucose level, causing liver damage, and inducing intestinal inflammation, by altering the gut microbiota of mice. The purified deep-sea bacteriophage DP105 and bacteriophage DP016 could cause mouse intestinal inflammation. The deep-sea-derived bacteriophages might shape the taxonomic composition and function of microbial communities in the mouse gut through influencing the bacterial colonization and selecting for specific virulence phenotypes (39, 40). When there is a mutualistic relationship between the eukaryotic host and the gut bacteria, the presence of virulent bacteriophages might be deleterious (39). In this context, our findings demonstrated that the viruses derived from the deep sea might be biosecurity threats to human health for the first time.

Except for the pathogenic viruses that directly infect human cells, bacteriophages, one of the most abundant types of viruses, can alter the composition of the gut bacterial community of humans (41). The bacteriophage community is involved in human diseases, such as inflammatory bowel diseases including ulcerative colitis and Crohn’s disease (24, 42–44). The presence of bacteriophages can worsen the inflammatory symptoms of the gut by enhancing the function of bacterial pathogens (45). In the deep sea, bacteriophages are the most widespread and abundant entities with a great deal of genetic diversity (23, 46, 47). The bacteriophages have great effects on the biogeochemical cycles of deep-sea ecosystems by regulating microbial communities (23, 48–50). Through participating in the hosts’ metabolic pathways, the bacteriophages can compensate the hosts’ metabolisms to help the hosts adapt to deep-sea extreme environments, which is essential for host survival (23). In the present investigation, however, the findings revealed that some deep-sea bacteriophages could proliferate in the gut of mice to alter the bacterial communities of mouse gut and further to destroy the mouse health, leading to alien species invasion of deep-sea bacteriophages into mammal gut. Although there are differences in the gut microbiota and immune responses between mice and humans, the deep-sea viruses still pose a potential threat to human health. The influence of deep-sea viruses on human health merited being characterized in multiple models in the future. As one of the most ancient environments on the earth, the deep sea, unexplored by humans (51), is rich in diverse populations of viruses including pathogenetic bacteriophages (52–54). The ancient viruses in the bottom of the deep sea may be hazardous to human beings, just like Pandora’s box. With the increasing activities of humans in the deep sea in recent years, such as scientific and mineral explorations, more and more deep-sea samples are brought into the land (55, 56). Once the Pandora’s box rich in diverse viruses is opened, the deep-sea viruses including bacteriophages may contact humans to invade the gut microbiota, possibly resulting in significant damage to human health. Our findings revealed that the viruses that could invade the human gut microbiota were broadly in the bottoms of oceans all over the world. In this context, the biosecurity risks of deep-sea viruses should be evaluated before performing scientific research and mining in the deep sea.

## Data Availability

The data that support the findings of this study are openly available in the NCBI database (https://www.ncbi.nlm.nih.gov/) under GenBank accession no. PRJNA721276.

## SUPPLEMENTAL MATERIAL

Supplemental tables mbio.01217-25-s0001.docx Table S1 to S4.

Table S5 mbio.01217-25-s0002.xlsx Influence of deep-sea viruses on mouse health.

ASM does not own the copyrights to Supplemental Material that may be linked to, or accessed through, an article. The authors have granted ASM a non-exclusive, world-wide license to publish the Supplemental Material files. Please contact the corresponding author directly for reuse.
