# Supplementary material for: Revived ancient viruses from deep-sea ecosystems are biothreats by triggering gut dysbiosis
Source: mBio. 2025 Jul 11;16(8):e01217-25. doi: 10.1128/mbio.01217-25 (PMC12345240; doi:10.1128/mbio.01217-25)
Supplement: Supplemental tables — Table S1 to S4. [file mbio.01217-25-s0001.docx]

| **Table S1. The information of sampling locations.** | | | | | |
| --- | --- | --- | --- | --- | --- |
| **Sample** | **Latitude** | **Longitude** | **Depth (m)** | **Geographical environment** | **Ocean** |
| DP027 | 44°48′W | 26°6′N | 3475 | hydrothermal vent | Atlantic Ocean |
| DP028 | 44°54′W | 14°42′N | 6000 | hydrothermal vent | Atlantic Ocean |
| DP029 | 44°54′W | 14°42′N | 5501 | hydrothermal vent | Atlantic Ocean |
| DP031 | 14°18′W | 13°18′S | 3601 | hydrothermal vent | Atlantic Ocean |
| DP032 | 14°30′W | 13°30′S | 5069 | hydrothermal vent | Atlantic Ocean |
| DP039 | 0°54′E | 3°6′S | 3534 | hydrothermal vent | Atlantic Ocean |
| DP040 | 11°54′W | 19°18′S | 2450 | hydrothermal vent | Atlantic Ocean |
| DP041 | 11°54′W | 19°18′S | 3434 | hydrothermal vent | Atlantic Ocean |
| DP042 | 12°0′W | 19°12′S | 5330 | hydrothermal vent | Atlantic Ocean |
| DP043 | 13°18′W | 15°6′S | 5608 | hydrothermal vent | Atlantic Ocean |
| DP044 | 13°18′W | 15°6′S | 5200 | hydrothermal vent | Atlantic Ocean |
| DP046 | 12°48′W | 18°0′S | 4084 | hydrothermal vent | Atlantic Ocean |
| DP047 | 12°42′W | 18°24′S | 4438 | hydrothermal vent | Atlantic Ocean |
| DP048 | 13°18′W | 15°6′S | 3828 | hydrothermal vent | Atlantic Ocean |
| DP049 | 12°42′W | 18°24′S | 5132 | hydrothermal vent | Atlantic Ocean |
| DP083 | 14°30′W | 13°36′S | 2949 | hydrothermal vent | Atlantic Ocean |
| DP025 | 63°42′E | 3°36′N | 2428 | hydrothermal vent | Indian Ocean |
| DP026 | 63°42′E | 3°36′N | 3613 | hydrothermal vent | Indian Ocean |
| DP030 | 14°30′W | 13°30′S | 3155 | hydrothermal vent | Indian Ocean |
| DP050 | 49°42′E | 37°48′S | 2975 | hydrothermal vent | Indian Ocean |
| DP051 | 49°48′E | 37°36′S | 2976 | hydrothermal vent | Indian Ocean |
| DP052 | 49°48′E | 37°42′S | 2779 | hydrothermal vent | Indian Ocean |
| DP053 | 49°42′E | 37°42′S | 2295 | hydrothermal vent | Indian Ocean |
| DP054 | 48°12′E | 37°30′S | 2286 | hydrothermal vent | Indian Ocean |
| DP055 | 47°12′E | 38°12′S | 2284 | hydrothermal vent | Indian Ocean |
| DP056 | 46°42′E | 38°42′S | 2143 | hydrothermal vent | Indian Ocean |
| DP058 | 47°0′E | 38°36′S | 2826 | hydrothermal vent | Indian Ocean |
| DP081 | 49°6′E | 37°54′S | 2058 | hydrothermal vent | Indian Ocean |
| DP082 | 49°42′E | 37°48′S | 2625 | hydrothermal vent | Indian Ocean |
| DP085 | 63°54′E | 27°48′S | 3773 | mid-ocean ridge | Indian Ocean |
| DP086 | 70°0′E | 25°18′S | 2999 | mid-ocean ridge | Indian Ocean |
| DP087 | 54°18′E | 34°48′S | 3272 | mid-ocean ridge | Indian Ocean |
| DP088 | 54°12′E | 34°54′S | 3206 | mid-ocean ridge | Indian Ocean |
| DP089 | 54°30′E | 34°48′S | 3239 | mid-ocean ridge | Indian Ocean |
| DP090 | 54°36′E | 34°48′S | 2335 | mid-ocean ridge | Indian Ocean |
| DP091 | 50°18′E | 37°36′S | 1902 | mid-ocean ridge | Indian Ocean |
| DP092 | 50°18′E | 37°42′S | 1905 | mid-ocean ridge | Indian Ocean |
| DP093 | 50°12′E | 37°48′S | 1905 | mid-ocean ridge | Indian Ocean |
| DP094 | 50°6′E | 37°54′S | 1912 | mid-ocean ridge | Indian Ocean |
| DP095 | 49°42′E | 37°42′S | 2987 | mid-ocean ridge | Indian Ocean |
| DP096 | 52°54′E | 35°54′S | 2621 | mid-ocean ridge | Indian Ocean |
| DP097 | 52°48′E | 35°54′S | 2624 | mid-ocean ridge | Indian Ocean |
| DP098 | 49°30′E | 37°36′S | 2294 | mid-ocean ridge | Indian Ocean |
| DP099 | 49°36′E | 37°42′S | 2297 | mid-ocean ridge | Indian Ocean |
| DP100 | 47°24′E | 37°42′S | 2699 | mid-ocean ridge | Indian Ocean |
| DP104 | 46°18′E | 37°42′S | 5679 | mid-ocean ridge | Indian Ocean |
| DP105 | 46°18′E | 37°48′S | 5665 | mid-ocean ridge | Indian Ocean |
| DP107 | 46°24′E | 37°42′S | 5955 | mid-ocean ridge | Indian Ocean |
| DP114 | 45°48′E | 37°18′S | 3921 | mid-ocean ridge | Indian Ocean |
| DP117 | 52°36′E | 34°6′S | 5548 | seamount | Indian Ocean |
| DP118 | 48°30′E | 37°42′S | 2502 | seamount | Indian Ocean |
| DP119 | 48°24′E | 37°42′S | 3189 | seamount | Indian Ocean |
| DP120 | 48°18′E | 38°12′S | 2864 | seamount | Indian Ocean |
| DP122 | 49°18′E | 37°42′S | 2380 | seamount | Indian Ocean |
| DP123 | 48°24′E | 38°0′S | 2318 | seamount | Indian Ocean |
| DP124 | 48°30′E | 38°6′S | 2774 | seamount | Indian Ocean |
| DP125 | 48°12′E | 38°0′S | 2871 | seamount | Indian Ocean |
| DP126 | 48°18′E | 38°6′S | 2519 | seamount | Indian Ocean |
| DP127 | 46°30′E | 38°54′S | 1332 | seamount | Indian Ocean |
| DP128 | 46°36′E | 38°48′S | 3251 | seamount | Indian Ocean |
| DP129 | 55°18′E | 34°24′S | 3057 | seamount | Indian Ocean |
| DP130 | 55°12′E | 34°24′S | 2556 | seamount | Indian Ocean |
| DP131 | 55°6′E | 34°24′S | 3332 | seamount | Indian Ocean |
| DP132 | 49°42′E | 37°54′S | 1535 | seamount | Indian Ocean |
| DP133 | 49°48′E | 37°42′S | 1523 | seamount | Indian Ocean |
| DP134 | 49°54′E | 37°48′S | 1496 | seamount | Indian Ocean |
| DP135 | 49°54′E | 37°54′S | 1486 | seamount | Indian Ocean |
| DP136 | 49°48′E | 37°48′S | 1512 | seamount | Indian Ocean |
| DP137 | 49°42′E | 37°54′S | 1513 | seamount | Indian Ocean |
| DP138 | 49°36′E | 37°30′S | 1511 | seamount | Indian Ocean |
| DP003 | 111°18′E | 18°0′N | 1884 | cold seep | Pacific Ocean |
| DP004 | 115°18′E | 20°0′N | 1154 | cold seep | Pacific Ocean |
| DP005 | 111°6′E | 17°36′N | 1765 | cold seep | Pacific Ocean |
| DP006 | 111°0′E | 17°36′N | 1736 | cold seep | Pacific Ocean |
| DP007 | 110°42′E | 17°18′N | 1604 | cold seep | Pacific Ocean |
| DP008 | 115°18′E | 19°54′N | 1310 | cold seep | Pacific Ocean |
| DP009 | 110°48′E | 17°18′N | 1627 | cold seep | Pacific Ocean |
| DP010 | 111°6′E | 17°42′N | 1855 | cold seep | Pacific Ocean |
| DP016 | 137°30′E | 8°0′N | 4159 | hadal trench | Pacific Ocean |
| DP017 | 137°30′E | 8°6′N | 4993 | hadal trench | Pacific Ocean |
| DP018 | 137°36′E | 8°6′N | 6173 | hadal trench | Pacific Ocean |
| DP019 | 137°36′E | 8°0′N | 6582 | hadal trench | Pacific Ocean |
| DP020 | 137°48′E | 8°0′N | 6682 | hadal trench | Pacific Ocean |
| DP021 | 138°42′E | 9°36′N | 5100 | hadal trench | Pacific Ocean |
| DP022 | 138°48′E | 9°36′N | 4500 | hadal trench | Pacific Ocean |
| DP059 | 154°18′W | 8°30′N | 5028 | hydrothermal vent | Pacific Ocean |
| DP060 | 153°18′W | 10°30′N | 4961 | hydrothermal vent | Pacific Ocean |
| DP061 | 153°12′W | 12°54′N | 5340 | hydrothermal vent | Pacific Ocean |
| DP062 | 154°48′W | 9°48′N | 5302 | hydrothermal vent | Pacific Ocean |
| DP063 | 154°24′W | 9°48′N | 5125 | hydrothermal vent | Pacific Ocean |
| DP064 | 154°18′W | 9°48′N | 5267 | hydrothermal vent | Pacific Ocean |
| DP065 | 154°48′W | 9°36′N | 5226 | hydrothermal vent | Pacific Ocean |
| DP066 | 154°12′W | 9°36′N | 5207 | hydrothermal vent | Pacific Ocean |
| DP067 | 154°24′W | 9°36′N | 5277 | hydrothermal vent | Pacific Ocean |
| DP068 | 154°36′W | 9°36′N | 5217 | hydrothermal vent | Pacific Ocean |
| DP069 | 154°36′W | 9°30′N | 5188 | hydrothermal vent | Pacific Ocean |
| DP070 | 154°18′W | 9°30′N | 5134 | hydrothermal vent | Pacific Ocean |
| DP071 | 154°42′W | 9°48′N | 5294 | hydrothermal vent | Pacific Ocean |
| DP072 | 154°18′W | 9°42′N | 5261 | hydrothermal vent | Pacific Ocean |
| DP073 | 154°36′W | 9°36′N | 5143 | hydrothermal vent | Pacific Ocean |
| DP074 | 154°42′W | 9°30′N | 5240 | hydrothermal vent | Pacific Ocean |
| DP075 | 152°24′W | 17°48′N | 5212 | hydrothermal vent | Pacific Ocean |
| DP076 | 151°30′W | 18°12′N | 5627 | hydrothermal vent | Pacific Ocean |
| DP077 | 153°36′W | 17°12′N | 5786 | hydrothermal vent | Pacific Ocean |
| DP078 | 174°30′W | 9°42′N | 5868 | hydrothermal vent | Pacific Ocean |
| DP139 | 105°24′E | 30°30′N | 1275 | hadal trench | Pacific Ocean |

| **Table S2. Diversities of mouse gut bacterial communities*.** | | | | | | |
| --- | --- | --- | --- | --- | --- | --- |
| Sample ID | Number of reads | Number of operational taxonomic unit (OTU) | Coverage | Shannon index | Simpson index | Chao1 estimator |
| DP003-3-C-F1 | 27,679 | 341 | 0.9981 | 4.09 | 0.0344 | 390 |
| DP003-3-C-F2 | 27,679 | 249 | 0.9976 | 3.53 | 0.0608 | 328 |
| DP003-3-C-F3 | 27,679 | 337 | 0.9978 | 4.09 | 0.0392 | 385 |
| DP003-3-C-M1 | 27,679 | 341 | 0.9967 | 3.08 | 0.1782 | 473 |
| DP003-3-C-M2 | 27,679 | 379 | 0.9962 | 3.09 | 0.1415 | 488 |
| DP003-3-C-M3 | 27,679 | 307 | 0.9969 | 3.78 | 0.0479 | 424 |
| DP003-3-E-F1 | 27,679 | 296 | 0.9967 | 2.53 | 0.1884 | 372 |
| DP003-3-E-F2 | 27,679 | 358 | 0.9973 | 3.51 | 0.0851 | 423 |
| DP003-3-E-F3 | 27,679 | 283 | 0.9968 | 2.52 | 0.1855 | 376 |
| DP003-3-E-M1 | 27,679 | 368 | 0.9961 | 3.11 | 0.1305 | 506 |
| DP003-3-E-M2 | 27,679 | 381 | 0.9963 | 3.68 | 0.0592 | 498 |
| DP003-3-E-M3 | 27,679 | 355 | 0.9971 | 3.9 | 0.0428 | 443 |
| DP003-10-C-F1 | 27,679 | 221 | 0.9981 | 3.08 | 0.105 | 276 |
| DP003-10-C-F2 | 27,679 | 329 | 0.9978 | 3.94 | 0.0513 | 378 |
| DP003-10-C-F3 | 27,679 | 306 | 0.9977 | 3.48 | 0.0762 | 369 |
| DP003-10-C-M1 | 27,679 | 340 | 0.9967 | 3.41 | 0.1016 | 451 |
| DP003-10-C-M2 | 27,679 | 259 | 0.9969 | 3.01 | 0.0834 | 398 |
| DP003-10-C-M3 | 27,679 | 367 | 0.9975 | 3.39 | 0.0855 | 409 |
| DP003-10-E-F1 | 27,679 | 379 | 0.9969 | 3.63 | 0.1005 | 496 |
| DP003-10-E-F2 | 27,679 | 339 | 0.9969 | 3.58 | 0.0573 | 422 |
| DP003-10-E-F3 | 27,679 | 448 | 0.996 | 3.74 | 0.0747 | 572 |
| DP003-10-E-M1 | 27,679 | 453 | 0.9954 | 3.4 | 0.1059 | 591 |
| DP003-10-E-M2 | 27,679 | 424 | 0.9961 | 3.13 | 0.1609 | 531 |
| DP003-10-E-M3 | 27,679 | 530 | 0.9952 | 4.19 | 0.0546 | 679 |
| DP004-3-C-F1 | 27,679 | 454 | 0.9968 | 4.09 | 0.0421 | 535 |
| DP004-3-C-F2 | 27,679 | 446 | 0.9973 | 4.32 | 0.0361 | 501 |
| DP004-3-C-F3 | 27,679 | 440 | 0.997 | 4.5 | 0.0251 | 521 |
| DP004-3-C-M1 | 27,679 | 392 | 0.9962 | 2.76 | 0.136 | 486 |
| DP004-3-C-M2 | 27,679 | 552 | 0.9966 | 4.28 | 0.0398 | 626 |
| DP004-3-C-M3 | 27,679 | 503 | 0.9967 | 4.43 | 0.0301 | 574 |
| DP004-3-E-F1 | 27,679 | 338 | 0.9979 | 3.7 | 0.0805 | 419 |
| DP004-3-E-F2 | 27,679 | 342 | 0.9975 | 3.03 | 0.1554 | 415 |
| DP004-3-E-F3 | 27,679 | 294 | 0.997 | 2.74 | 0.16 | 371 |
| DP004-3-E-M1 | 27,679 | 292 | 0.9969 | 2.1 | 0.2918 | 375 |
| DP004-3-E-M2 | 27,679 | 332 | 0.9971 | 3.27 | 0.0969 | 409 |
| DP004-3-E-M3 | 27,679 | 368 | 0.9974 | 3.59 | 0.0847 | 439 |
| DP004-10-C-F1 | 27,679 | 491 | 0.9966 | 4.36 | 0.029 | 575 |
| DP004-10-C-F2 | 27,679 | 470 | 0.9966 | 4.35 | 0.0332 | 582 |
| DP004-10-C-F3 | 27,679 | 477 | 0.9968 | 4.3 | 0.0353 | 560 |
| DP004-10-C-M1 | 27,679 | 323 | 0.9965 | 2.48 | 0.1478 | 409 |
| DP004-10-C-M2 | 27,679 | 495 | 0.9962 | 3.88 | 0.0564 | 588 |
| DP004-10-C-M3 | 27,679 | 426 | 0.9974 | 4.38 | 0.0292 | 480 |
| DP004-10-E-F1 | 27,679 | 221 | 0.9974 | 2.67 | 0.1146 | 296 |
| DP004-10-E-F2 | 27,679 | 377 | 0.9971 | 3.64 | 0.0825 | 446 |
| DP004-10-E-F3 | 27,679 | 433 | 0.9951 | 3.6 | 0.0689 | 625 |
| DP004-10-E-M1 | 27,679 | 431 | 0.9945 | 2.6 | 0.193 | 593 |
| DP004-10-E-M2 | 27,679 | 281 | 0.9963 | 3.05 | 0.0871 | 416 |
| DP004-10-E-M3 | 27,679 | 398 | 0.9954 | 3.16 | 0.1119 | 539 |
| DP005-3-C-F1 | 27,679 | 446 | 0.9963 | 3.88 | 0.0574 | 523 |
| DP005-3-C-F2 | 27,679 | 427 | 0.9963 | 3.88 | 0.06 | 556 |
| DP005-3-C-F3 | 27,679 | 408 | 0.9967 | 3.94 | 0.05 | 485 |
| DP005-3-C-M1 | 27,679 | 387 | 0.9966 | 3.61 | 0.082 | 482 |
| DP005-3-C-M2 | 27,679 | 411 | 0.9967 | 3.85 | 0.0641 | 504 |
| DP005-3-C-M3 | 27,679 | 326 | 0.997 | 3.3 | 0.0986 | 407 |
| DP005-3-E-F1 | 27,679 | 445 | 0.9968 | 4.12 | 0.0629 | 522 |
| DP005-3-E-F2 | 27,679 | 428 | 0.9965 | 3.73 | 0.0723 | 512 |
| DP005-3-E-F3 | 27,679 | 492 | 0.996 | 3.77 | 0.0868 | 577 |
| DP005-3-E-M1 | 27,679 | 491 | 0.9962 | 4.58 | 0.0206 | 585 |
| DP005-3-E-M2 | 27,679 | 430 | 0.9961 | 4.06 | 0.0386 | 541 |
| DP005-3-E-M3 | 27,679 | 461 | 0.9961 | 4.19 | 0.0397 | 566 |
| DP005-10-C-F1 | 27,679 | 433 | 0.9964 | 3.9 | 0.0469 | 526 |
| DP005-10-C-F2 | 27,679 | 401 | 0.9953 | 3.77 | 0.0485 | 562 |
| DP005-10-C-F3 | 27,679 | 321 | 0.9964 | 3.68 | 0.0481 | 447 |
| DP005-10-C-M1 | 27,679 | 440 | 0.996 | 3.67 | 0.0921 | 575 |
| DP005-10-C-M2 | 27,679 | 431 | 0.9961 | 3.94 | 0.0654 | 579 |
| DP005-10-C-M3 | 27,679 | 334 | 0.9962 | 3.25 | 0.1164 | 460 |
| DP005-10-E-F1 | 27,679 | 489 | 0.9961 | 4.01 | 0.0568 | 596 |
| DP005-10-E-F2 | 27,679 | 501 | 0.9972 | 4.34 | 0.0455 | 543 |
| DP005-10-E-F3 | 27,679 | 256 | 0.9969 | 3.1 | 0.122 | 426 |
| DP005-10-E-M1 | 27,679 | 419 | 0.9956 | 3.83 | 0.0508 | 608 |
| DP005-10-E-M2 | 27,679 | 398 | 0.9966 | 3.83 | 0.048 | 476 |
| DP005-10-E-M3 | 27,679 | 311 | 0.9967 | 3.17 | 0.0912 | 402 |
| DP006-3-C-F1 | 27,679 | 443 | 0.9965 | 4.24 | 0.0427 | 575 |
| DP006-3-C-F2 | 27,679 | 407 | 0.996 | 3.16 | 0.0954 | 510 |
| DP006-3-C-F3 | 27,679 | 396 | 0.9966 | 3.63 | 0.0641 | 482 |
| DP006-3-C-M1 | 27,679 | 448 | 0.9967 | 3.78 | 0.061 | 520 |
| DP006-3-C-M2 | 27,679 | 538 | 0.9964 | 4.4 | 0.0312 | 617 |
| DP006-3-C-M3 | 27,679 | 366 | 0.9966 | 3.02 | 0.163 | 447 |
| DP006-3-E-F1 | 27,679 | 213 | 0.9983 | 2.85 | 0.1173 | 253 |
| DP006-3-E-F2 | 27,679 | 283 | 0.9979 | 3.49 | 0.0691 | 333 |
| DP006-3-E-F3 | 27,679 | 406 | 0.9972 | 3.99 | 0.0502 | 477 |
| DP006-3-E-M1 | 27,679 | 308 | 0.9971 | 2.58 | 0.284 | 393 |
| DP006-3-E-M2 | 27,679 | 360 | 0.9971 | 3.9 | 0.0439 | 448 |
| DP006-3-E-M3 | 27,679 | 343 | 0.997 | 3.31 | 0.0787 | 407 |
| DP006-10-C-F1 | 27,679 | 442 | 0.9966 | 4.18 | 0.0426 | 543 |
| DP006-10-C-F2 | 27,679 | 528 | 0.9967 | 4.29 | 0.0356 | 587 |
| DP006-10-C-F3 | 27,679 | 407 | 0.9963 | 3.65 | 0.0605 | 497 |
| DP006-10-C-M1 | 27,679 | 458 | 0.9962 | 3.73 | 0.0661 | 563 |
| DP006-10-C-M2 | 27,679 | 395 | 0.9965 | 3 | 0.1053 | 480 |
| DP006-10-C-M3 | 27,679 | 388 | 0.9963 | 3.1 | 0.1478 | 482 |
| DP006-10-E-F1 | 27,679 | 371 | 0.9972 | 3.43 | 0.1024 | 425 |
| DP006-10-E-F2 | 27,679 | 268 | 0.9979 | 3.23 | 0.1012 | 306 |
| DP006-10-E-F3 | 27,679 | 381 | 0.9973 | 3.62 | 0.0782 | 450 |
| DP006-10-E-M1 | 27,679 | 365 | 0.997 | 3.46 | 0.0775 | 442 |
| DP006-10-E-M2 | 27,679 | 389 | 0.9964 | 3.97 | 0.0354 | 522 |
| DP006-10-E-M3 | 27,679 | 408 | 0.9976 | 4.33 | 0.0249 | 458 |
| DP007-3-C-F1 | 27,679 | 465 | 0.9961 | 3.71 | 0.0633 | 581 |
| DP007-3-C-F2 | 27,679 | 469 | 0.9972 | 4.13 | 0.0413 | 520 |
| DP007-3-C-F3 | 27,679 | 301 | 0.9973 | 2.97 | 0.111 | 364 |
| DP007-3-C-M1 | 27,679 | 508 | 0.9958 | 3.56 | 0.0907 | 607 |
| DP007-3-C-M2 | 27,679 | 376 | 0.9964 | 2.95 | 0.1298 | 473 |
| DP007-3-C-M3 | 27,679 | 458 | 0.9964 | 3.28 | 0.1169 | 533 |
| DP007-3-E-F1 | 27,679 | 507 | 0.9958 | 4.12 | 0.0449 | 596 |
| DP007-3-E-F2 | 27,679 | 488 | 0.9956 | 3.75 | 0.0611 | 605 |
| DP007-3-E-F3 | 27,679 | 463 | 0.9954 | 4 | 0.0455 | 592 |
| DP007-3-E-M1 | 27,679 | 552 | 0.9966 | 4.13 | 0.0527 | 625 |
| DP007-3-E-M2 | 27,679 | 427 | 0.9962 | 3.23 | 0.1045 | 515 |
| DP007-3-E-M3 | 27,679 | 528 | 0.996 | 3.94 | 0.0566 | 621 |
| DP007-10-C-F1 | 27,679 | 476 | 0.996 | 3.76 | 0.0589 | 596 |
| DP007-10-C-F2 | 27,679 | 499 | 0.9966 | 4.18 | 0.039 | 585 |
| DP007-10-C-F3 | 27,679 | 354 | 0.9962 | 3.07 | 0.1057 | 451 |
| DP007-10-C-M1 | 27,679 | 531 | 0.9967 | 3.99 | 0.059 | 600 |
| DP007-10-C-M2 | 27,679 | 430 | 0.9964 | 3.28 | 0.1022 | 527 |
| DP007-10-C-M3 | 27,679 | 447 | 0.9967 | 3.29 | 0.1051 | 511 |
| DP007-10-E-F1 | 27,679 | 392 | 0.9957 | 3.61 | 0.0702 | 555 |
| DP007-10-E-F2 | 27,679 | 446 | 0.997 | 4.11 | 0.042 | 525 |
| DP007-10-E-F3 | 27,679 | 502 | 0.9967 | 4.28 | 0.0406 | 582 |
| DP007-10-E-M1 | 27,679 | 474 | 0.9964 | 4.11 | 0.0379 | 554 |
| DP007-10-E-M2 | 27,679 | 500 | 0.9967 | 4.61 | 0.0216 | 585 |
| DP007-10-E-M3 | 27,679 | 451 | 0.9969 | 4.15 | 0.0328 | 520 |
| DP008-3-C-F1 | 27,679 | 533 | 0.9964 | 4.31 | 0.0391 | 601 |
| DP008-3-C-F2 | 27,679 | 547 | 0.9965 | 4.58 | 0.0294 | 633 |
| DP008-3-C-F3 | 27,679 | 447 | 0.9958 | 3.88 | 0.0672 | 575 |
| DP008-3-C-F4 | 27,679 | 500 | 0.9958 | 4 | 0.0493 | 594 |
| DP008-3-C-F5 | 27,679 | 571 | 0.9943 | 4.12 | 0.0442 | 762 |
| DP008-3-E-F1 | 27,679 | 546 | 0.9968 | 4.85 | 0.0174 | 628 |
| DP008-3-E-F2 | 27,679 | 425 | 0.9965 | 4.01 | 0.0493 | 522 |
| DP008-3-E-F3 | 27,679 | 626 | 0.9945 | 4.72 | 0.02 | 786 |
| DP008-3-E-M1 | 27,679 | 548 | 0.9961 | 4.56 | 0.0229 | 679 |
| DP008-3-E-M2 | 27,679 | 449 | 0.9967 | 4.05 | 0.0611 | 551 |
| DP008-10-C-F1 | 27,679 | 533 | 0.9955 | 4.27 | 0.0409 | 686 |
| DP008-10-C-F2 | 27,679 | 575 | 0.9961 | 4.62 | 0.0256 | 663 |
| DP008-10-C-F3 | 27,679 | 420 | 0.9956 | 3.68 | 0.0628 | 547 |
| DP008-10-C-F4 | 27,679 | 514 | 0.9951 | 4.01 | 0.0476 | 641 |
| DP008-10-C-F5 | 27,679 | 531 | 0.9955 | 4.09 | 0.0444 | 650 |
| DP008-10-E-F1 | 27,679 | 520 | 0.9961 | 4.31 | 0.035 | 601 |
| DP008-10-E-F2 | 27,679 | 408 | 0.9964 | 3.29 | 0.1121 | 505 |
| DP008-10-E-F3 | 27,679 | 404 | 0.9959 | 3.37 | 0.0788 | 500 |
| DP008-10-E-M1 | 27,679 | 430 | 0.9961 | 3.53 | 0.0842 | 526 |
| DP008-10-E-M2 | 27,679 | 360 | 0.9962 | 3.57 | 0.0572 | 562 |
| DP009-3-C-F1 | 27,679 | 323 | 0.996 | 3.05 | 0.1154 | 485 |
| DP009-3-C-F2 | 27,679 | 414 | 0.9958 | 3.2 | 0.1084 | 566 |
| DP009-3-C-F3 | 27,679 | 350 | 0.9964 | 2.99 | 0.1148 | 442 |
| DP009-3-C-M1 | 27,679 | 517 | 0.9964 | 3.98 | 0.0585 | 599 |
| DP009-3-C-M2 | 27,679 | 416 | 0.9964 | 3.6 | 0.065 | 508 |
| DP009-3-C-M3 | 27,679 | 464 | 0.9964 | 3.52 | 0.0764 | 551 |
| DP009-3-E-F1 | 27,679 | 368 | 0.9976 | 3.83 | 0.0467 | 418 |
| DP009-3-E-F2 | 27,679 | 439 | 0.997 | 4.15 | 0.032 | 520 |
| DP009-3-E-F3 | 27,679 | 431 | 0.9975 | 4.65 | 0.0196 | 502 |
| DP009-3-E-M1 | 27,679 | 279 | 0.997 | 2.52 | 0.1776 | 339 |
| DP009-3-E-M2 | 27,679 | 433 | 0.9971 | 4.14 | 0.0468 | 501 |
| DP009-3-E-M3 | 27,679 | 330 | 0.9974 | 2.46 | 0.2478 | 403 |
| DP009-10-C-F1 | 27,679 | 366 | 0.9965 | 3.12 | 0.1152 | 432 |
| DP009-10-C-F2 | 27,679 | 389 | 0.9962 | 3 | 0.125 | 471 |
| DP009-10-C-F3 | 27,679 | 392 | 0.9958 | 3.16 | 0.1052 | 518 |
| DP009-10-C-M1 | 27,679 | 452 | 0.9967 | 3.28 | 0.1107 | 537 |
| DP009-10-C-M2 | 27,679 | 426 | 0.9967 | 3.71 | 0.064 | 496 |
| DP009-10-C-M3 | 27,679 | 418 | 0.9965 | 3.26 | 0.0948 | 495 |
| DP009-10-E-F1 | 27,679 | 370 | 0.9971 | 3.8 | 0.0492 | 451 |
| DP009-10-E-F2 | 27,679 | 338 | 0.9969 | 3.45 | 0.0657 | 421 |
| DP009-10-E-F3 | 27,679 | 324 | 0.9978 | 4.03 | 0.0358 | 376 |
| DP009-10-E-M1 | 27,679 | 373 | 0.9963 | 3.48 | 0.0767 | 493 |
| DP009-10-E-M2 | 27,679 | 354 | 0.9971 | 3.47 | 0.08 | 416 |
| DP010-3-C-F2 | 27,679 | 371 | 0.9959 | 3.57 | 0.0568 | 515 |
| DP010-3-C-F3 | 27,679 | 423 | 0.9962 | 3.47 | 0.0739 | 516 |
| DP010-3-C-M1 | 27,679 | 466 | 0.996 | 3.89 | 0.0487 | 571 |
| DP010-3-C-M2 | 27,679 | 494 | 0.997 | 4.1 | 0.0424 | 553 |
| DP010-3-C-M3 | 27,679 | 428 | 0.9962 | 3.39 | 0.0764 | 531 |
| DP010-3-E-F1 | 27,679 | 513 | 0.9941 | 3.58 | 0.0684 | 707 |
| DP010-3-E-F2 | 27,679 | 477 | 0.997 | 4.41 | 0.0355 | 558 |
| DP010-3-E-F3 | 27,679 | 336 | 0.998 | 3.73 | 0.0608 | 374 |
| DP010-3-E-M1 | 27,679 | 398 | 0.997 | 3.39 | 0.084 | 471 |
| DP010-3-E-M2 | 27,679 | 461 | 0.9964 | 3.62 | 0.0676 | 540 |
| DP010-3-E-M3 | 27,679 | 406 | 0.9973 | 3.76 | 0.0511 | 472 |
| DP010-10-C-F1 | 27,679 | 332 | 0.9974 | 3.86 | 0.0391 | 403 |
| DP010-10-C-F2 | 27,679 | 447 | 0.9957 | 3.85 | 0.0474 | 577 |
| DP010-10-C-F3 | 27,679 | 453 | 0.9958 | 3.64 | 0.0596 | 547 |
| DP010-10-C-M1 | 27,679 | 395 | 0.9952 | 3.29 | 0.0853 | 568 |
| DP010-10-C-M2 | 27,679 | 482 | 0.9968 | 4.15 | 0.0416 | 550 |
| DP010-10-C-M3 | 27,679 | 534 | 0.9939 | 3.74 | 0.0673 | 728 |
| DP010-10-E-F1 | 27,679 | 507 | 0.996 | 4.41 | 0.0314 | 618 |
| DP010-10-E-F2 | 27,679 | 458 | 0.995 | 3.52 | 0.0847 | 639 |
| DP010-10-E-F3 | 27,679 | 526 | 0.9965 | 4.6 | 0.0294 | 614 |
| DP010-10-E-M1 | 27,679 | 528 | 0.9968 | 4.73 | 0.0192 | 596 |
| DP010-10-E-M2 | 27,679 | 428 | 0.9969 | 3.66 | 0.0829 | 487 |
| DP010-10-E-M3 | 27,679 | 357 | 0.9967 | 3.45 | 0.0821 | 435 |
| DP016-3-C-F1 | 27,679 | 321 | 0.9973 | 3.75 | 0.0834 | 390 |
| DP016-3-C-F2 | 27,679 | 408 | 0.996 | 3.85 | 0.0433 | 538 |
| DP016-3-C-F3 | 27,679 | 468 | 0.9969 | 4.64 | 0.0209 | 547 |
| DP016-3-C-M1 | 27,679 | 198 | 0.9977 | 2.07 | 0.2373 | 265 |
| DP016-3-C-M2 | 27,679 | 372 | 0.9978 | 4.33 | 0.025 | 424 |
| DP016-3-C-M3 | 27,679 | 450 | 0.9971 | 4.38 | 0.0424 | 529 |
| DP016-3-E-F1 | 27,679 | 357 | 0.9971 | 3.54 | 0.0696 | 426 |
| DP016-3-E-F2 | 27,679 | 387 | 0.997 | 3.38 | 0.106 | 454 |
| DP016-3-E-F3 | 27,679 | 374 | 0.9977 | 3.52 | 0.0706 | 410 |
| DP016-3-E-M1 | 27,679 | 306 | 0.9966 | 3.37 | 0.07 | 428 |
| DP016-3-E-M2 | 27,679 | 287 | 0.9972 | 3.02 | 0.1062 | 368 |
| DP016-3-E-M3 | 27,679 | 340 | 0.9973 | 3.89 | 0.0357 | 402 |
| DP016-10-C-F1 | 27,679 | 281 | 0.9975 | 3.28 | 0.081 | 370 |
| DP016-10-C-F2 | 27,679 | 321 | 0.9978 | 3.88 | 0.0608 | 391 |
| DP016-10-C-F3 | 27,679 | 401 | 0.9969 | 3.87 | 0.0462 | 473 |
| DP016-10-C-M1 | 27,679 | 327 | 0.9966 | 2.31 | 0.3178 | 443 |
| DP016-10-C-M2 | 27,679 | 407 | 0.9974 | 3.65 | 0.0754 | 460 |
| DP016-10-C-M3 | 27,679 | 352 | 0.9979 | 4.1 | 0.0378 | 403 |
| DP016-10-E-F1 | 27,679 | 347 | 0.9969 | 3.32 | 0.0775 | 436 |
| DP016-10-E-F2 | 27,679 | 406 | 0.9975 | 4.28 | 0.0348 | 473 |
| DP016-10-E-F3 | 27,679 | 411 | 0.9974 | 3.82 | 0.0783 | 480 |
| DP016-10-E-M1 | 27,679 | 420 | 0.9973 | 4.56 | 0.0214 | 495 |
| DP016-10-E-M2 | 27,679 | 299 | 0.997 | 3.71 | 0.0704 | 469 |
| DP016-10-E-M3 | 27,679 | 440 | 0.9977 | 4.24 | 0.0403 | 485 |
| DP017-3-C-F1 | 27,679 | 481 | 0.9974 | 4.8 | 0.0162 | 539 |
| DP017-3-C-F2 | 27,679 | 347 | 0.9967 | 3.74 | 0.0496 | 447 |
| DP017-3-C-F3 | 27,679 | 529 | 0.9971 | 4.76 | 0.0182 | 599 |
| DP017-3-C-M1 | 27,679 | 540 | 0.9963 | 4.78 | 0.0169 | 635 |
| DP017-3-C-M2 | 27,679 | 501 | 0.9969 | 4.26 | 0.0425 | 564 |
| DP017-3-C-M3 | 27,679 | 522 | 0.9967 | 4.39 | 0.037 | 603 |
| DP017-3-E-F1 | 27,679 | 347 | 0.9983 | 4.07 | 0.0463 | 377 |
| DP017-3-E-F2 | 27,679 | 400 | 0.9969 | 3.84 | 0.0583 | 498 |
| DP017-3-E-F3 | 27,679 | 464 | 0.9968 | 4.23 | 0.0531 | 555 |
| DP017-3-E-M1 | 27,679 | 408 | 0.9978 | 4.71 | 0.0167 | 469 |
| DP017-3-E-M2 | 27,679 | 292 | 0.9977 | 3.38 | 0.0823 | 349 |
| DP017-3-E-M3 | 27,679 | 393 | 0.9971 | 3.65 | 0.0882 | 470 |
| DP017-10-C-F1 | 27,679 | 487 | 0.9973 | 4.82 | 0.017 | 549 |
| DP017-10-C-F2 | 27,679 | 447 | 0.997 | 4.27 | 0.0312 | 518 |
| DP017-10-C-F3 | 27,679 | 490 | 0.9971 | 4.41 | 0.0258 | 549 |
| DP017-10-C-M1 | 27,679 | 394 | 0.9961 | 3.37 | 0.0829 | 507 |
| DP017-10-C-M2 | 27,679 | 524 | 0.9973 | 4.62 | 0.0324 | 579 |
| DP017-10-C-M3 | 27,679 | 527 | 0.997 | 4.5 | 0.0371 | 595 |
| DP017-10-E-F1 | 27,679 | 327 | 0.9971 | 2.99 | 0.1415 | 395 |
| DP017-10-E-F2 | 27,679 | 370 | 0.9977 | 3.84 | 0.0512 | 419 |
| DP017-10-E-F3 | 27,679 | 293 | 0.9981 | 3.68 | 0.053 | 339 |
| DP017-10-E-M1 | 27,679 | 323 | 0.9978 | 3.86 | 0.0431 | 384 |
| DP017-10-E-M2 | 27,679 | 408 | 0.9977 | 3.99 | 0.0601 | 447 |
| DP017-10-E-M3 | 27,679 | 399 | 0.9973 | 4.21 | 0.0328 | 478 |
| DP018-3-C-F1 | 27,679 | 453 | 0.9976 | 4.41 | 0.0317 | 488 |
| DP018-3-C-F2 | 27,679 | 475 | 0.9967 | 4.37 | 0.0289 | 564 |
| DP018-3-C-F3 | 27,679 | 368 | 0.9963 | 3.38 | 0.0826 | 480 |
| DP018-3-C-M1 | 27,679 | 445 | 0.9967 | 4.03 | 0.0419 | 514 |
| DP018-3-C-M2 | 27,679 | 556 | 0.9969 | 4.66 | 0.0256 | 628 |
| DP018-3-C-M3 | 27,679 | 485 | 0.997 | 4.15 | 0.0582 | 539 |
| DP018-3-E-F1 | 27,679 | 449 | 0.9968 | 4.18 | 0.0391 | 529 |
| DP018-3-E-F2 | 27,679 | 367 | 0.9973 | 4.07 | 0.0421 | 419 |
| DP018-3-E-F3 | 27,679 | 421 | 0.9963 | 4.08 | 0.0344 | 508 |
| DP018-3-E-M1 | 27,679 | 356 | 0.9966 | 3.39 | 0.0796 | 443 |
| DP018-3-E-M2 | 27,679 | 397 | 0.9964 | 3.67 | 0.0579 | 486 |
| DP018-3-E-M3 | 27,679 | 420 | 0.9964 | 4.01 | 0.0412 | 510 |
| DP018-10-C-F1 | 27,679 | 450 | 0.9973 | 4.33 | 0.0382 | 509 |
| DP018-10-C-F2 | 27,679 | 449 | 0.9964 | 4 | 0.0439 | 536 |
| DP018-10-C-F3 | 27,679 | 362 | 0.9966 | 3.34 | 0.0865 | 450 |
| DP018-10-C-M1 | 27,679 | 440 | 0.9962 | 4.03 | 0.0432 | 554 |
| DP018-10-C-M2 | 27,679 | 529 | 0.9968 | 4.39 | 0.0374 | 592 |
| DP018-10-C-M3 | 27,679 | 459 | 0.9969 | 4.1 | 0.0552 | 535 |
| DP018-10-E-F1 | 27,679 | 479 | 0.9965 | 4.32 | 0.0292 | 578 |
| DP018-10-E-F2 | 27,679 | 440 | 0.9972 | 4.53 | 0.0244 | 511 |
| DP018-10-E-F3 | 27,679 | 420 | 0.997 | 4.15 | 0.0379 | 483 |
| DP018-10-E-M1 | 27,679 | 423 | 0.9965 | 4 | 0.0364 | 534 |
| DP018-10-E-M2 | 27,679 | 415 | 0.997 | 3.44 | 0.1404 | 483 |
| DP018-10-E-M3 | 27,679 | 375 | 0.9964 | 3.69 | 0.0608 | 474 |
| DP019-3-C-F1 | 27,679 | 416 | 0.9971 | 3.8 | 0.062 | 478 |
| DP019-3-C-F2 | 27,679 | 421 | 0.9961 | 3.88 | 0.0479 | 547 |
| DP019-3-C-F3 | 27,679 | 415 | 0.9961 | 4.08 | 0.0339 | 556 |
| DP019-3-C-M1 | 27,679 | 448 | 0.997 | 4.28 | 0.0347 | 554 |
| DP019-3-C-M2 | 27,679 | 427 | 0.9974 | 4.24 | 0.031 | 481 |
| DP019-3-C-M3 | 27,679 | 368 | 0.998 | 3.94 | 0.0463 | 416 |
| DP019-3-E-F1 | 27,679 | 495 | 0.9967 | 4.75 | 0.0161 | 580 |
| DP019-3-E-F2 | 27,679 | 387 | 0.9964 | 3.7 | 0.0649 | 490 |
| DP019-3-E-F3 | 27,679 | 467 | 0.9969 | 4.37 | 0.0304 | 548 |
| DP019-3-E-M1 | 27,679 | 451 | 0.9972 | 4.44 | 0.0241 | 519 |
| DP019-3-E-M2 | 27,679 | 431 | 0.9967 | 4.3 | 0.0293 | 516 |
| DP019-3-E-M3 | 27,679 | 324 | 0.9973 | 3.96 | 0.0367 | 406 |
| DP019-10-C-F1 | 27,679 | 332 | 0.9965 | 3.44 | 0.0603 | 455 |
| DP019-10-C-F2 | 27,679 | 317 | 0.9966 | 3.07 | 0.1118 | 421 |
| DP019-10-C-F3 | 27,679 | 279 | 0.9977 | 3.4 | 0.0898 | 363 |
| DP019-10-C-M1 | 27,679 | 397 | 0.9969 | 3.82 | 0.0634 | 493 |
| DP019-10-C-M2 | 27,679 | 397 | 0.9966 | 3.69 | 0.0637 | 466 |
| DP019-10-C-M3 | 27,679 | 365 | 0.9968 | 3.88 | 0.0428 | 461 |
| DP019-10-E-F1 | 27,679 | 433 | 0.9965 | 3.87 | 0.0519 | 521 |
| DP019-10-E-F2 | 27,679 | 424 | 0.9966 | 3.36 | 0.1088 | 505 |
| DP019-10-E-F3 | 27,679 | 448 | 0.9967 | 4.04 | 0.0489 | 533 |
| DP019-10-E-M1 | 27,679 | 301 | 0.997 | 3.36 | 0.0884 | 393 |
| DP019-10-E-M2 | 27,679 | 297 | 0.9977 | 3.23 | 0.1168 | 356 |
| DP019-10-E-M3 | 27,679 | 283 | 0.997 | 2.46 | 0.2373 | 370 |
| DP020-3-C-F1 | 27,679 | 446 | 0.997 | 3.84 | 0.1104 | 527 |
| DP020-3-C-F2 | 27,679 | 495 | 0.9968 | 4.77 | 0.0198 | 595 |
| DP020-3-C-F3 | 27,679 | 455 | 0.9978 | 4.65 | 0.0213 | 509 |
| DP020-3-C-M1 | 27,679 | 496 | 0.9964 | 4.08 | 0.0633 | 601 |
| DP020-3-C-M2 | 27,679 | 462 | 0.9971 | 4.35 | 0.0331 | 526 |
| DP020-3-C-M3 | 27,679 | 450 | 0.9971 | 4.49 | 0.0242 | 522 |
| DP020-3-E-F1 | 27,679 | 436 | 0.9974 | 4.65 | 0.0211 | 497 |
| DP020-3-E-F2 | 27,679 | 403 | 0.9975 | 4.58 | 0.0177 | 470 |
| DP020-3-E-F3 | 27,679 | 423 | 0.9979 | 4.56 | 0.0207 | 482 |
| DP020-3-E-M1 | 27,679 | 464 | 0.9973 | 4.88 | 0.014 | 566 |
| DP020-3-E-M2 | 27,679 | 485 | 0.9974 | 4.71 | 0.0176 | 537 |
| DP020-3-E-M3 | 27,679 | 469 | 0.9975 | 4.83 | 0.0182 | 534 |
| DP020-10-C-F1 | 27,679 | 507 | 0.9973 | 4.79 | 0.0208 | 573 |
| DP020-10-C-F2 | 27,679 | 478 | 0.9966 | 4.18 | 0.0673 | 571 |
| DP020-10-C-F3 | 27,679 | 460 | 0.9974 | 4.85 | 0.0155 | 546 |
| DP020-10-C-M1 | 27,679 | 430 | 0.9971 | 4.08 | 0.0627 | 494 |
| DP020-10-C-M2 | 27,679 | 444 | 0.997 | 4.13 | 0.0494 | 512 |
| DP020-10-C-M3 | 27,679 | 450 | 0.9973 | 4.49 | 0.0238 | 504 |
| DP020-10-E-F1 | 27,679 | 406 | 0.9962 | 3.83 | 0.0522 | 520 |
| DP020-10-E-F2 | 27,679 | 263 | 0.9971 | 2.68 | 0.162 | 397 |
| DP020-10-E-F3 | 27,679 | 420 | 0.997 | 4.41 | 0.0315 | 523 |
| DP020-10-E-M1 | 27,679 | 519 | 0.9966 | 4.78 | 0.0183 | 600 |
| DP020-10-E-M2 | 27,679 | 476 | 0.9972 | 4.54 | 0.0289 | 562 |
| DP020-10-E-M3 | 27,679 | 488 | 0.9971 | 4.66 | 0.0224 | 560 |
| DP021-3-C-F1 | 27,679 | 395 | 0.9963 | 3.51 | 0.0853 | 498 |
| DP021-3-C-F2 | 27,679 | 454 | 0.9965 | 4.14 | 0.0416 | 573 |
| DP021-3-C-F3 | 27,679 | 310 | 0.997 | 3.65 | 0.0508 | 377 |
| DP021-3-C-M1 | 27,679 | 429 | 0.9973 | 4.24 | 0.0327 | 491 |
| DP021-3-C-M2 | 27,679 | 463 | 0.997 | 4.57 | 0.0204 | 536 |
| DP021-3-C-M3 | 27,679 | 461 | 0.9958 | 3.71 | 0.1133 | 567 |
| DP021-3-E-F1 | 27,679 | 387 | 0.9962 | 3.83 | 0.0529 | 488 |
| DP021-3-E-F2 | 27,679 | 341 | 0.9974 | 3.87 | 0.0386 | 387 |
| DP021-3-E-F3 | 27,679 | 389 | 0.9966 | 3.53 | 0.0887 | 496 |
| DP021-3-E-M1 | 27,679 | 451 | 0.9957 | 4.28 | 0.0288 | 589 |
| DP021-3-E-M2 | 27,679 | 301 | 0.9973 | 3.52 | 0.066 | 372 |
| DP021-3-E-M3 | 27,679 | 425 | 0.9958 | 3.63 | 0.0717 | 536 |
| DP021-10-C-F1 | 27,679 | 485 | 0.9966 | 4.51 | 0.0218 | 582 |
| DP021-10-C-F2 | 27,679 | 444 | 0.9965 | 4.35 | 0.0254 | 561 |
| DP021-10-C-F3 | 27,679 | 476 | 0.9971 | 4.33 | 0.0479 | 537 |
| DP021-10-C-M1 | 27,679 | 363 | 0.9973 | 3.82 | 0.0654 | 458 |
| DP021-10-C-M2 | 27,679 | 489 | 0.9965 | 4.49 | 0.0269 | 577 |
| DP021-10-C-M3 | 27,679 | 421 | 0.9971 | 4.21 | 0.0433 | 488 |
| DP021-10-E-F1 | 27,679 | 257 | 0.9979 | 3.36 | 0.0895 | 302 |
| DP021-10-E-F2 | 27,679 | 255 | 0.9972 | 3.33 | 0.0725 | 344 |
| DP021-10-E-F3 | 27,679 | 443 | 0.9967 | 4.2 | 0.0326 | 530 |
| DP021-10-E-M1 | 27,679 | 417 | 0.9975 | 4.12 | 0.0434 | 467 |
| DP021-10-E-M2 | 27,679 | 295 | 0.9976 | 3.93 | 0.0391 | 378 |
| DP025-3-C-F1 | 27,679 | 527 | 0.9963 | 4.48 | 0.0325 | 618 |
| DP025-3-C-F2 | 27,679 | 476 | 0.9952 | 3.92 | 0.0495 | 647 |
| DP025-3-C-F3 | 27,679 | 504 | 0.9969 | 4.82 | 0.0193 | 600 |
| DP025-3-C-M1 | 27,679 | 506 | 0.9972 | 4.56 | 0.029 | 570 |
| DP025-3-C-M2 | 27,679 | 395 | 0.996 | 3.25 | 0.0911 | 502 |
| DP025-3-C-M3 | 27,679 | 517 | 0.9967 | 4.35 | 0.0405 | 606 |
| DP025-3-E-F1 | 27,679 | 395 | 0.9969 | 3.61 | 0.0659 | 471 |
| DP025-3-E-F2 | 27,679 | 446 | 0.9967 | 3.95 | 0.0454 | 514 |
| DP025-3-E-F3 | 27,679 | 500 | 0.9962 | 4.13 | 0.0481 | 587 |
| DP025-3-E-M1 | 27,679 | 532 | 0.997 | 4.73 | 0.0182 | 599 |
| DP025-3-E-M2 | 27,679 | 454 | 0.9978 | 4.46 | 0.0254 | 497 |
| DP025-3-E-M3 | 27,679 | 552 | 0.9969 | 4.89 | 0.0163 | 635 |
| DP025-10-C-F1 | 27,679 | 540 | 0.997 | 4.6 | 0.0258 | 601 |
| DP025-10-C-F2 | 27,679 | 441 | 0.9957 | 3.69 | 0.0617 | 556 |
| DP025-10-C-F3 | 27,679 | 500 | 0.9975 | 4.89 | 0.0172 | 547 |
| DP025-10-C-M1 | 27,679 | 455 | 0.9967 | 3.81 | 0.0666 | 518 |
| DP025-10-C-M2 | 27,679 | 428 | 0.9967 | 3.53 | 0.0754 | 508 |
| DP025-10-C-M3 | 27,679 | 472 | 0.9971 | 4.19 | 0.0382 | 534 |
| DP025-10-E-F1 | 27,679 | 470 | 0.9966 | 3.94 | 0.0761 | 537 |
| DP025-10-E-F2 | 27,679 | 377 | 0.9972 | 3.2 | 0.1451 | 447 |
| DP025-10-E-F3 | 27,679 | 459 | 0.9969 | 3.95 | 0.0477 | 540 |
| DP025-10-E-M1 | 27,679 | 520 | 0.9969 | 4.59 | 0.0249 | 593 |
| DP025-10-E-M2 | 27,679 | 469 | 0.9971 | 4.4 | 0.0251 | 533 |
| DP025-10-E-M3 | 27,679 | 524 | 0.9973 | 4.68 | 0.02 | 579 |
| DP026-3-C-F1 | 27,679 | 311 | 0.9978 | 3.64 | 0.049 | 364 |
| DP026-3-C-F2 | 27,679 | 385 | 0.9977 | 4.26 | 0.0338 | 432 |
| DP026-3-C-F3 | 27,679 | 325 | 0.9977 | 3.9 | 0.0412 | 399 |
| DP026-3-C-M1 | 27,679 | 482 | 0.9966 | 4.56 | 0.0209 | 566 |
| DP026-3-C-M2 | 27,679 | 461 | 0.9967 | 4.21 | 0.0363 | 575 |
| DP026-3-C-M3 | 27,679 | 396 | 0.9968 | 3.99 | 0.0364 | 470 |
| DP026-3-E-F1 | 27,679 | 277 | 0.9974 | 2.94 | 0.131 | 325 |
| DP026-3-E-F2 | 27,679 | 221 | 0.998 | 3.16 | 0.0807 | 265 |
| DP026-3-E-F3 | 27,679 | 317 | 0.9973 | 3.34 | 0.079 | 385 |
| DP026-3-E-M1 | 27,679 | 440 | 0.9977 | 4.64 | 0.0217 | 487 |
| DP026-3-E-M2 | 27,679 | 364 | 0.9974 | 4.1 | 0.0411 | 446 |
| DP026-3-E-M3 | 27,679 | 331 | 0.9978 | 3.98 | 0.0469 | 390 |
| DP026-10-C-F1 | 27,679 | 345 | 0.998 | 4.01 | 0.0373 | 383 |
| DP026-10-C-F2 | 27,679 | 385 | 0.9978 | 4.31 | 0.0299 | 436 |
| DP026-10-C-F3 | 27,679 | 343 | 0.9984 | 4.32 | 0.027 | 381 |
| DP026-10-C-M1 | 27,679 | 468 | 0.9963 | 4.35 | 0.0278 | 573 |
| DP026-10-C-M2 | 27,679 | 447 | 0.9964 | 4.02 | 0.0442 | 535 |
| DP026-10-C-M3 | 27,679 | 418 | 0.9978 | 4.38 | 0.0264 | 459 |
| DP026-10-E-F1 | 27,679 | 390 | 0.9975 | 4.2 | 0.0272 | 452 |
| DP026-10-E-F2 | 27,679 | 332 | 0.9965 | 3.78 | 0.0476 | 485 |
| DP026-10-E-F3 | 27,679 | 397 | 0.9973 | 3.97 | 0.0429 | 460 |
| DP026-10-E-M1 | 27,679 | 412 | 0.9972 | 4.03 | 0.0455 | 477 |
| DP026-10-E-M2 | 27,679 | 320 | 0.9972 | 3.39 | 0.1233 | 399 |
| DP026-10-E-M3 | 27,679 | 297 | 0.9977 | 2.9 | 0.2443 | 362 |
| DP027-3-C-F1 | 27,679 | 499 | 0.9968 | 4.27 | 0.0456 | 574 |
| DP027-3-C-F2 | 27,679 | 537 | 0.9969 | 4.6 | 0.0301 | 620 |
| DP027-3-C-F3 | 27,679 | 500 | 0.9966 | 4.16 | 0.0539 | 593 |
| DP027-3-C-M1 | 27,679 | 486 | 0.9965 | 3.48 | 0.1176 | 562 |
| DP027-3-C-M2 | 27,679 | 511 | 0.9968 | 4.25 | 0.0519 | 575 |
| DP027-3-C-M3 | 27,679 | 371 | 0.996 | 3.39 | 0.0738 | 482 |
| DP027-3-E-F1 | 27,679 | 405 | 0.9977 | 4.1 | 0.0486 | 477 |
| DP027-3-E-F2 | 27,679 | 422 | 0.9973 | 4.37 | 0.0288 | 501 |
| DP027-3-E-F3 | 27,679 | 330 | 0.9981 | 3.94 | 0.0511 | 371 |
| DP027-3-E-M1 | 27,679 | 398 | 0.9977 | 4.13 | 0.039 | 444 |
| DP027-3-E-M2 | 27,679 | 322 | 0.9975 | 2.05 | 0.4134 | 375 |
| DP027-3-E-M3 | 27,679 | 383 | 0.9978 | 3.38 | 0.1052 | 423 |
| DP027-10-C-F1 | 27,679 | 511 | 0.9972 | 4.65 | 0.0269 | 583 |
| DP027-10-C-F2 | 27,679 | 523 | 0.997 | 4.5 | 0.0281 | 583 |
| DP027-10-C-F3 | 27,679 | 480 | 0.9963 | 3.64 | 0.1034 | 566 |
| DP027-10-C-M1 | 27,679 | 484 | 0.9958 | 3.64 | 0.0772 | 601 |
| DP027-10-C-M2 | 27,679 | 504 | 0.9968 | 4.1 | 0.0617 | 581 |
| DP027-10-C-M3 | 27,679 | 470 | 0.9954 | 3.68 | 0.0644 | 613 |
| DP027-10-E-F1 | 27,679 | 289 | 0.9975 | 3.36 | 0.0755 | 343 |
| DP027-10-E-F2 | 27,679 | 341 | 0.997 | 2.77 | 0.1692 | 433 |
| DP027-10-E-F3 | 27,679 | 390 | 0.9973 | 3.76 | 0.0733 | 449 |
| DP027-10-E-M1 | 27,679 | 406 | 0.9979 | 4.41 | 0.0252 | 447 |
| DP027-10-E-M2 | 27,679 | 429 | 0.9979 | 4.26 | 0.0435 | 466 |
| DP027-10-E-M3 | 27,679 | 387 | 0.9974 | 4 | 0.0439 | 456 |
| DP028-3-C-F1 | 27,679 | 255 | 0.9975 | 3.03 | 0.1259 | 338 |
| DP028-3-C-F2 | 27,679 | 306 | 0.9974 | 3.28 | 0.1133 | 405 |
| DP028-3-C-F3 | 27,679 | 336 | 0.998 | 4 | 0.0447 | 385 |
| DP028-3-C-M1 | 27,679 | 356 | 0.997 | 2.98 | 0.1325 | 425 |
| DP028-3-C-M2 | 27,679 | 319 | 0.998 | 3.06 | 0.1506 | 354 |
| DP028-3-C-M3 | 27,679 | 377 | 0.9977 | 3.73 | 0.0615 | 436 |
| DP028-3-E-F1 | 27,679 | 409 | 0.9966 | 4.04 | 0.0377 | 518 |
| DP028-3-E-F2 | 27,679 | 432 | 0.9969 | 4.07 | 0.0511 | 513 |
| DP028-3-E-F3 | 27,679 | 407 | 0.997 | 4.18 | 0.0409 | 494 |
| DP028-3-E-M1 | 27,679 | 487 | 0.9972 | 4.86 | 0.0146 | 551 |
| DP028-3-E-M2 | 27,679 | 371 | 0.9972 | 3.81 | 0.0878 | 439 |
| DP028-3-E-M3 | 27,679 | 520 | 0.9966 | 4.74 | 0.019 | 611 |
| DP028-10-C-F1 | 27,679 | 323 | 0.9982 | 3.99 | 0.0394 | 351 |
| DP028-10-C-F2 | 27,679 | 272 | 0.9977 | 2.89 | 0.1333 | 321 |
| DP028-10-C-F3 | 27,679 | 313 | 0.9974 | 3.46 | 0.0802 | 382 |
| DP028-10-C-M1 | 27,679 | 378 | 0.9975 | 3.62 | 0.0787 | 443 |
| DP028-10-C-M2 | 27,679 | 345 | 0.9978 | 3.98 | 0.0522 | 406 |
| DP028-10-E-F1 | 27,679 | 327 | 0.9977 | 3.45 | 0.0817 | 380 |
| DP028-10-E-F2 | 27,679 | 349 | 0.9977 | 4.15 | 0.0362 | 399 |
| DP028-10-E-F3 | 27,679 | 446 | 0.997 | 4.3 | 0.032 | 533 |
| DP028-10-E-M1 | 27,679 | 326 | 0.9981 | 3.93 | 0.0537 | 389 |
| DP028-10-E-M2 | 27,679 | 417 | 0.9974 | 3.95 | 0.0556 | 488 |
| DP028-10-E-M3 | 27,679 | 318 | 0.998 | 4.03 | 0.0408 | 362 |
| DP029-3-C-F1 | 27,679 | 343 | 0.9974 | 3.65 | 0.0493 | 416 |
| DP029-3-C-F2 | 27,679 | 355 | 0.9977 | 4.29 | 0.0262 | 418 |
| DP029-3-C-F3 | 27,679 | 324 | 0.9983 | 3.93 | 0.0419 | 347 |
| DP029-3-C-M1 | 27,679 | 283 | 0.998 | 4.08 | 0.0319 | 345 |
| DP029-3-C-M2 | 27,679 | 352 | 0.9981 | 4.36 | 0.0263 | 398 |
| DP029-3-C-M3 | 27,679 | 312 | 0.9981 | 4.02 | 0.0418 | 369 |
| DP029-3-E-F1 | 27,679 | 297 | 0.9979 | 3.4 | 0.097 | 358 |
| DP029-3-E-F2 | 27,679 | 360 | 0.9981 | 4 | 0.0814 | 393 |
| DP029-3-E-F3 | 27,679 | 415 | 0.9974 | 3.78 | 0.1005 | 486 |
| DP029-3-E-M1 | 27,679 | 428 | 0.9978 | 4.44 | 0.0356 | 474 |
| DP029-3-E-M2 | 27,679 | 270 | 0.9975 | 1.9 | 0.4637 | 351 |
| DP029-3-E-M3 | 27,679 | 367 | 0.9971 | 3.89 | 0.0593 | 446 |
| DP029-10-C-F1 | 27,679 | 436 | 0.9976 | 4.73 | 0.0174 | 492 |
| DP029-10-C-F2 | 27,679 | 375 | 0.9974 | 4.28 | 0.0292 | 426 |
| DP029-10-C-F3 | 27,679 | 376 | 0.9978 | 4.07 | 0.0375 | 421 |
| DP029-10-C-M1 | 27,679 | 273 | 0.9976 | 2.88 | 0.1259 | 316 |
| DP029-10-C-M2 | 27,679 | 314 | 0.9978 | 3.2 | 0.1125 | 359 |
| DP029-10-C-M3 | 27,679 | 318 | 0.9973 | 3.2 | 0.1069 | 382 |
| DP029-10-E-F1 | 27,679 | 406 | 0.9976 | 4.52 | 0.0222 | 475 |
| DP029-10-E-F2 | 27,679 | 425 | 0.9971 | 4.13 | 0.0466 | 500 |
| DP029-10-E-F3 | 27,679 | 399 | 0.9978 | 4.44 | 0.0275 | 442 |
| DP029-10-E-M1 | 27,679 | 420 | 0.9971 | 4.19 | 0.0294 | 492 |
| DP029-10-E-M2 | 27,679 | 382 | 0.9971 | 4.01 | 0.0383 | 448 |
| DP029-10-E-M3 | 27,679 | 402 | 0.9974 | 4.19 | 0.0344 | 466 |
| DP030-3-C-F1 | 27,679 | 549 | 0.9965 | 4.79 | 0.0189 | 646 |
| DP030-3-C-F2 | 27,679 | 501 | 0.9967 | 4.45 | 0.0406 | 575 |
| DP030-3-C-F3 | 27,679 | 528 | 0.9966 | 4.54 | 0.0325 | 614 |
| DP030-3-C-M1 | 27,679 | 260 | 0.9967 | 2.42 | 0.2337 | 351 |
| DP030-3-C-M2 | 27,679 | 473 | 0.9965 | 3.55 | 0.0954 | 555 |
| DP030-3-C-M3 | 27,679 | 340 | 0.9971 | 3.11 | 0.1002 | 412 |
| DP030-3-E-F1 | 27,679 | 376 | 0.9974 | 4.24 | 0.0359 | 449 |
| DP030-3-E-F2 | 27,679 | 447 | 0.997 | 4.32 | 0.037 | 521 |
| DP030-3-E-F3 | 27,679 | 430 | 0.9968 | 4.22 | 0.0294 | 508 |
| DP030-3-E-M1 | 27,679 | 455 | 0.9975 | 4.44 | 0.0256 | 506 |
| DP030-3-E-M2 | 27,679 | 417 | 0.9972 | 4.39 | 0.0333 | 484 |
| DP030-3-E-M3 | 27,679 | 392 | 0.9974 | 4.21 | 0.0301 | 480 |
| DP030-10-C-F1 | 27,679 | 543 | 0.9969 | 4.73 | 0.0232 | 603 |
| DP030-10-C-F2 | 27,679 | 512 | 0.9965 | 4.51 | 0.037 | 611 |
| DP030-10-C-F3 | 27,679 | 513 | 0.9965 | 4.42 | 0.0377 | 586 |
| DP030-10-C-M1 | 27,679 | 324 | 0.9965 | 2.64 | 0.2072 | 432 |
| DP030-10-C-M2 | 27,679 | 494 | 0.9965 | 3.72 | 0.0874 | 582 |
| DP030-10-C-M3 | 27,679 | 353 | 0.9964 | 3.11 | 0.1022 | 482 |
| DP030-10-E-F1 | 27,679 | 350 | 0.9964 | 3.4 | 0.0806 | 481 |
| DP030-10-E-F2 | 27,679 | 414 | 0.9971 | 3.81 | 0.0652 | 481 |
| DP030-10-E-F3 | 27,679 | 406 | 0.9977 | 4.17 | 0.0334 | 447 |
| DP030-10-E-M1 | 27,679 | 426 | 0.9974 | 4.13 | 0.0432 | 508 |
| DP030-10-E-M2 | 27,679 | 368 | 0.9973 | 3.82 | 0.0621 | 450 |
| DP030-10-E-M3 | 27,679 | 419 | 0.9961 | 3.83 | 0.0482 | 542 |
| DP031-3-C-F1 | 27,679 | 336 | 0.9968 | 3.4 | 0.0801 | 468 |
| DP031-3-C-F2 | 27,679 | 369 | 0.9969 | 3.43 | 0.0793 | 447 |
| DP031-3-C-F3 | 27,679 | 440 | 0.9966 | 3.87 | 0.0693 | 537 |
| DP031-3-C-M1 | 27,679 | 350 | 0.9975 | 3.82 | 0.052 | 423 |
| DP031-3-C-M2 | 27,679 | 477 | 0.9971 | 4.62 | 0.0202 | 544 |
| DP031-3-C-M3 | 27,679 | 392 | 0.9972 | 3.71 | 0.0706 | 469 |
| DP031-3-E-F1 | 27,679 | 348 | 0.998 | 4.28 | 0.0292 | 426 |
| DP031-3-E-F2 | 27,679 | 312 | 0.9972 | 3.55 | 0.0591 | 385 |
| DP031-3-E-F3 | 27,679 | 412 | 0.9978 | 4.63 | 0.0216 | 460 |
| DP031-3-E-M1 | 27,679 | 399 | 0.9969 | 3.76 | 0.0743 | 501 |
| DP031-3-E-M2 | 27,679 | 427 | 0.9974 | 3.91 | 0.0627 | 479 |
| DP031-3-E-M3 | 27,679 | 366 | 0.9968 | 3.51 | 0.1208 | 453 |
| DP031-10-C-F1 | 27,679 | 443 | 0.9975 | 4.4 | 0.0347 | 505 |
| DP031-10-C-F2 | 27,679 | 460 | 0.9972 | 4.52 | 0.0238 | 517 |
| DP031-10-C-F3 | 27,679 | 421 | 0.9978 | 4.15 | 0.0511 | 469 |
| DP031-10-C-M1 | 27,679 | 465 | 0.9966 | 4.32 | 0.031 | 552 |
| DP031-10-C-M2 | 27,679 | 427 | 0.9977 | 4.39 | 0.0232 | 473 |
| DP031-10-C-M3 | 27,679 | 313 | 0.9975 | 2.3 | 0.2867 | 368 |
| DP031-10-E-F1 | 27,679 | 365 | 0.997 | 3.43 | 0.0937 | 428 |
| DP031-10-E-F2 | 27,679 | 411 | 0.9978 | 4.49 | 0.022 | 458 |
| DP031-10-E-F3 | 27,679 | 391 | 0.9979 | 4.44 | 0.0234 | 443 |
| DP031-10-E-M1 | 27,679 | 379 | 0.9964 | 3.18 | 0.1156 | 484 |
| DP032-3-C-F1 | 27,679 | 498 | 0.9964 | 4.27 | 0.0531 | 588 |
| DP032-3-C-F2 | 27,679 | 507 | 0.9974 | 4.41 | 0.0283 | 548 |
| DP032-3-C-F3 | 27,679 | 509 | 0.997 | 4.64 | 0.0243 | 594 |
| DP032-3-C-M1 | 27,679 | 400 | 0.9963 | 3.34 | 0.0899 | 501 |
| DP032-3-C-M2 | 27,679 | 261 | 0.9973 | 2.7 | 0.1668 | 345 |
| DP032-3-C-M3 | 27,679 | 368 | 0.9969 | 3.09 | 0.1146 | 438 |
| DP032-3-E-F1 | 27,679 | 408 | 0.9979 | 4 | 0.0548 | 445 |
| DP032-3-E-F2 | 27,679 | 438 | 0.997 | 4.1 | 0.0447 | 527 |
| DP032-3-E-F3 | 27,679 | 270 | 0.9968 | 2.6 | 0.2098 | 405 |
| DP032-3-E-M1 | 27,679 | 419 | 0.9971 | 4.19 | 0.0363 | 485 |
| DP032-3-E-M2 | 27,679 | 466 | 0.9966 | 4.4 | 0.0258 | 557 |
| DP032-3-E-M3 | 27,679 | 411 | 0.9973 | 4.19 | 0.0347 | 472 |
| DP032-10-C-F1 | 27,679 | 512 | 0.9966 | 4.43 | 0.0436 | 594 |
| DP032-10-C-F2 | 27,679 | 528 | 0.997 | 4.55 | 0.0251 | 594 |
| DP032-10-C-F3 | 27,679 | 509 | 0.9973 | 4.83 | 0.0179 | 579 |
| DP032-10-C-M1 | 27,679 | 275 | 0.9968 | 2.76 | 0.1567 | 384 |
| DP032-10-C-M2 | 27,679 | 355 | 0.9963 | 2.91 | 0.1353 | 460 |
| DP032-10-C-M3 | 27,679 | 450 | 0.9968 | 3.69 | 0.0656 | 530 |
| DP032-10-E-F1 | 27,679 | 339 | 0.9971 | 3.26 | 0.0834 | 402 |
| DP032-10-E-F2 | 27,679 | 410 | 0.9972 | 4.03 | 0.0474 | 477 |
| DP032-10-E-F3 | 27,679 | 337 | 0.9966 | 3.56 | 0.0542 | 428 |
| DP032-10-E-M1 | 27,679 | 395 | 0.9975 | 3.66 | 0.0715 | 444 |
| DP032-10-E-M2 | 27,679 | 257 | 0.9975 | 3.01 | 0.1133 | 326 |
| DP032-10-E-M3 | 27,679 | 325 | 0.9976 | 2.84 | 0.2084 | 383 |
| DP039-3-C-F1 | 27,679 | 496 | 0.9968 | 4.72 | 0.0199 | 581 |
| DP039-3-C-F2 | 27,679 | 483 | 0.9959 | 4.32 | 0.0383 | 624 |
| DP039-3-C-F3 | 27,679 | 419 | 0.9978 | 4.26 | 0.0293 | 455 |
| DP039-3-C-M1 | 27,679 | 473 | 0.997 | 4.68 | 0.019 | 547 |
| DP039-3-C-M2 | 27,679 | 391 | 0.9966 | 3.88 | 0.0446 | 475 |
| DP039-3-C-M3 | 27,679 | 442 | 0.9977 | 4.59 | 0.0225 | 488 |
| DP039-3-E-F1 | 27,679 | 327 | 0.9976 | 3.77 | 0.061 | 388 |
| DP039-3-E-F2 | 27,679 | 440 | 0.9961 | 4.16 | 0.0295 | 566 |
| DP039-3-E-F3 | 27,679 | 454 | 0.9974 | 4.68 | 0.0172 | 508 |
| DP039-3-E-M1 | 27,679 | 450 | 0.9967 | 4.32 | 0.0262 | 529 |
| DP039-3-E-M2 | 27,679 | 403 | 0.9967 | 3.77 | 0.0509 | 480 |
| DP039-3-E-M3 | 27,679 | 466 | 0.9973 | 4.73 | 0.0185 | 532 |
| DP039-10-C-F1 | 27,679 | 485 | 0.9961 | 4.32 | 0.0335 | 590 |
| DP039-10-C-F2 | 27,679 | 474 | 0.9963 | 4.39 | 0.0297 | 617 |
| DP039-10-C-F3 | 27,679 | 575 | 0.9955 | 4.15 | 0.0401 | 669 |
| DP039-10-C-M1 | 27,679 | 456 | 0.997 | 4.62 | 0.0206 | 548 |
| DP039-10-C-M2 | 27,679 | 417 | 0.9971 | 4.4 | 0.0249 | 510 |
| DP039-10-C-M3 | 27,679 | 442 | 0.9971 | 4.65 | 0.0208 | 538 |
| DP039-10-E-F1 | 27,679 | 308 | 0.9968 | 3.49 | 0.0688 | 430 |
| DP039-10-E-F2 | 27,679 | 389 | 0.9962 | 3.94 | 0.0441 | 525 |
| DP039-10-E-F3 | 27,679 | 464 | 0.9966 | 4.77 | 0.0154 | 563 |
| DP039-10-E-M1 | 27,679 | 500 | 0.9968 | 4.77 | 0.018 | 600 |
| DP039-10-E-M2 | 27,679 | 503 | 0.9974 | 4.68 | 0.025 | 555 |
| DP039-10-E-M3 | 27,679 | 410 | 0.9976 | 4.54 | 0.0244 | 477 |
| DP040-3-C-F1 | 27,679 | 506 | 0.9959 | 4.17 | 0.0432 | 635 |
| DP040-3-C-F2 | 27,679 | 455 | 0.9959 | 4.08 | 0.0385 | 560 |
| DP040-3-C-F3 | 27,679 | 538 | 0.996 | 4.65 | 0.0254 | 673 |
| DP040-3-C-M1 | 27,679 | 487 | 0.9962 | 4.55 | 0.0227 | 620 |
| DP040-3-C-M2 | 27,679 | 477 | 0.9964 | 4.28 | 0.0348 | 576 |
| DP040-3-C-M3 | 27,679 | 498 | 0.9962 | 4.28 | 0.0494 | 612 |
| DP040-3-E-F1 | 27,679 | 481 | 0.9974 | 4.5 | 0.0307 | 537 |
| DP040-3-E-F2 | 27,679 | 399 | 0.9957 | 3.54 | 0.0724 | 537 |
| DP040-3-E-F3 | 27,679 | 456 | 0.9976 | 4.91 | 0.0133 | 509 |
| DP040-3-E-M1 | 27,679 | 473 | 0.9963 | 4.23 | 0.0391 | 590 |
| DP040-3-E-M2 | 27,679 | 419 | 0.9972 | 3.71 | 0.098 | 489 |
| DP040-3-E-M3 | 27,679 | 500 | 0.9961 | 4.23 | 0.0483 | 623 |
| DP040-10-C-F1 | 27,679 | 478 | 0.9968 | 4.28 | 0.045 | 563 |
| DP040-10-C-F2 | 27,679 | 469 | 0.9966 | 4.72 | 0.0158 | 578 |
| DP040-10-C-F3 | 27,679 | 499 | 0.9967 | 4.7 | 0.0199 | 575 |
| DP040-10-C-M1 | 27,679 | 504 | 0.9962 | 4.36 | 0.0301 | 595 |
| DP040-10-C-M2 | 27,679 | 434 | 0.9961 | 3.96 | 0.0405 | 541 |
| DP040-10-C-M3 | 27,679 | 513 | 0.9969 | 4.74 | 0.0182 | 586 |
| DP040-10-E-F1 | 27,679 | 480 | 0.9963 | 4.74 | 0.0168 | 618 |
| DP040-10-E-F2 | 27,679 | 547 | 0.9959 | 4.62 | 0.023 | 654 |
| DP040-10-E-F3 | 27,679 | 556 | 0.9936 | 3.77 | 0.0641 | 737 |
| DP040-10-E-M1 | 27,679 | 499 | 0.9953 | 4.1 | 0.0479 | 624 |
| DP040-10-E-M2 | 27,679 | 527 | 0.9947 | 4.19 | 0.0348 | 703 |
| DP040-10-E-M3 | 27,679 | 564 | 0.9965 | 4.98 | 0.0128 | 655 |
| DP041-3-C-F1 | 27,679 | 480 | 0.9963 | 3.68 | 0.1161 | 603 |
| DP041-3-C-F2 | 27,679 | 505 | 0.9969 | 3.86 | 0.0986 | 571 |
| DP041-3-C-F3 | 27,679 | 293 | 0.9973 | 2.79 | 0.1954 | 377 |
| DP041-3-C-M1 | 27,679 | 398 | 0.9963 | 3.4 | 0.0741 | 480 |
| DP041-3-C-M2 | 27,679 | 418 | 0.9958 | 3.08 | 0.1149 | 533 |
| DP041-3-C-M3 | 27,679 | 444 | 0.9965 | 3.49 | 0.0803 | 518 |
| DP041-3-E-F1 | 27,679 | 403 | 0.9973 | 3.81 | 0.0806 | 473 |
| DP041-3-E-F2 | 27,679 | 353 | 0.9967 | 3.77 | 0.0753 | 471 |
| DP041-3-E-F3 | 27,679 | 501 | 0.9968 | 4.54 | 0.0229 | 576 |
| DP041-3-E-M1 | 27,679 | 459 | 0.998 | 4.75 | 0.0181 | 497 |
| DP041-3-E-M2 | 27,679 | 457 | 0.9979 | 4.7 | 0.023 | 496 |
| DP041-3-E-M3 | 27,679 | 415 | 0.9973 | 4.48 | 0.0235 | 490 |
| DP041-10-C-M1 | 27,679 | 431 | 0.9961 | 3.4 | 0.0814 | 532 |
| DP041-10-C-M2 | 27,679 | 408 | 0.9957 | 3.16 | 0.1066 | 560 |
| DP041-10-C-M3 | 27,679 | 418 | 0.9966 | 3.53 | 0.0706 | 499 |
| DP041-10-E-F1 | 27,679 | 440 | 0.9973 | 4.43 | 0.0262 | 519 |
| DP041-10-E-F2 | 27,679 | 281 | 0.9983 | 3.68 | 0.0548 | 323 |
| DP041-10-E-F3 | 27,679 | 437 | 0.9964 | 3.83 | 0.06 | 534 |
| DP041-10-E-M1 | 27,679 | 391 | 0.9967 | 3.62 | 0.0698 | 489 |
| DP041-10-E-M2 | 27,679 | 440 | 0.9973 | 4.45 | 0.0277 | 521 |
| DP041-10-E-M3 | 27,679 | 355 | 0.998 | 4.38 | 0.0263 | 410 |
| DP042-3-C-F1 | 27,679 | 503 | 0.9969 | 4.27 | 0.0517 | 566 |
| DP042-3-C-F2 | 27,679 | 518 | 0.9969 | 4.37 | 0.0492 | 607 |
| DP042-3-C-F3 | 27,679 | 503 | 0.9959 | 3.46 | 0.14 | 599 |
| DP042-3-C-M1 | 27,679 | 460 | 0.9966 | 3.54 | 0.0691 | 527 |
| DP042-3-C-M2 | 27,679 | 460 | 0.996 | 3.4 | 0.1035 | 569 |
| DP042-3-C-M3 | 27,679 | 429 | 0.997 | 3.73 | 0.0588 | 493 |
| DP042-3-E-F1 | 27,679 | 381 | 0.9975 | 4.45 | 0.0222 | 462 |
| DP042-3-E-F2 | 27,679 | 462 | 0.9968 | 4.29 | 0.0416 | 537 |
| DP042-3-E-F3 | 27,679 | 361 | 0.9972 | 2.85 | 0.2379 | 459 |
| DP042-3-E-M1 | 27,679 | 383 | 0.9969 | 3.3 | 0.153 | 472 |
| DP042-3-E-M2 | 27,679 | 406 | 0.9974 | 3.66 | 0.1247 | 471 |
| DP042-3-E-M3 | 27,679 | 409 | 0.9971 | 3.96 | 0.051 | 479 |
| DP042-10-C-M1 | 27,679 | 450 | 0.9963 | 3.65 | 0.0676 | 560 |
| DP042-10-C-M2 | 27,679 | 482 | 0.9969 | 3.97 | 0.0562 | 535 |
| DP042-10-C-M3 | 27,679 | 403 | 0.9964 | 3.53 | 0.0668 | 495 |
| DP042-10-E-F1 | 27,679 | 367 | 0.9974 | 4.29 | 0.025 | 461 |
| DP042-10-E-F2 | 27,679 | 428 | 0.9976 | 4.05 | 0.0529 | 482 |
| DP042-10-E-F3 | 27,679 | 320 | 0.9963 | 3.49 | 0.0621 | 476 |
| DP042-10-E-M1 | 27,679 | 404 | 0.9969 | 4.07 | 0.0355 | 505 |
| DP042-10-E-M2 | 27,679 | 372 | 0.9969 | 3.34 | 0.1291 | 450 |
| DP042-10-E-M3 | 27,679 | 436 | 0.997 | 4.43 | 0.0226 | 523 |
| DP043-3-C-F1 | 27,679 | 417 | 0.9969 | 3.85 | 0.0759 | 488 |
| DP043-3-C-F2 | 27,679 | 389 | 0.9976 | 4.26 | 0.0299 | 444 |
| DP043-3-C-F3 | 27,679 | 462 | 0.9971 | 4.27 | 0.0401 | 516 |
| DP043-3-C-M1 | 27,679 | 492 | 0.9967 | 4.47 | 0.0241 | 594 |
| DP043-3-C-M2 | 27,679 | 458 | 0.9966 | 4.13 | 0.0447 | 579 |
| DP043-3-C-M3 | 27,679 | 456 | 0.9966 | 3.93 | 0.0519 | 525 |
| DP043-3-E-F1 | 27,679 | 452 | 0.997 | 4.28 | 0.0341 | 524 |
| DP043-3-E-F2 | 27,679 | 363 | 0.997 | 3.63 | 0.0578 | 439 |
| DP043-3-E-F3 | 27,679 | 413 | 0.9958 | 3.62 | 0.0818 | 539 |
| DP043-3-E-M1 | 27,679 | 381 | 0.9968 | 3.74 | 0.0751 | 452 |
| DP043-3-E-M2 | 27,679 | 472 | 0.9969 | 4.29 | 0.0403 | 533 |
| DP043-3-E-M3 | 27,679 | 471 | 0.9959 | 3.75 | 0.0945 | 575 |
| DP043-10-C-F1 | 27,679 | 443 | 0.9966 | 4.1 | 0.0504 | 542 |
| DP043-10-C-F2 | 27,679 | 396 | 0.9965 | 4.11 | 0.0341 | 534 |
| DP043-10-C-F3 | 27,679 | 431 | 0.9973 | 4.17 | 0.0413 | 497 |
| DP043-10-C-M1 | 27,679 | 473 | 0.9964 | 4.26 | 0.0375 | 583 |
| DP043-10-C-M2 | 27,679 | 450 | 0.9971 | 4.2 | 0.0401 | 518 |
| DP043-10-C-M3 | 27,679 | 430 | 0.9969 | 3.84 | 0.0602 | 503 |
| DP043-10-E-F1 | 27,679 | 511 | 0.9969 | 4.12 | 0.0559 | 574 |
| DP043-10-E-F2 | 27,679 | 440 | 0.997 | 4.17 | 0.0382 | 535 |
| DP043-10-E-F3 | 27,679 | 497 | 0.9966 | 4.59 | 0.0273 | 615 |
| DP043-10-E-M1 | 27,679 | 404 | 0.997 | 3.88 | 0.0648 | 480 |
| DP043-10-E-M2 | 27,679 | 442 | 0.9966 | 4.11 | 0.0397 | 531 |
| DP043-10-E-M3 | 27,679 | 428 | 0.9967 | 3.93 | 0.0536 | 519 |
| DP044-3-C-F1 | 27,679 | 493 | 0.9971 | 4.38 | 0.0503 | 557 |
| DP044-3-C-F2 | 27,679 | 512 | 0.997 | 4.04 | 0.091 | 577 |
| DP044-3-C-F3 | 27,679 | 536 | 0.9968 | 4.42 | 0.0501 | 616 |
| DP044-3-C-M1 | 27,679 | 380 | 0.9968 | 3.55 | 0.0639 | 457 |
| DP044-3-C-M2 | 27,679 | 389 | 0.9964 | 3.28 | 0.0846 | 481 |
| DP044-3-C-M3 | 27,679 | 386 | 0.9973 | 3.59 | 0.0671 | 443 |
| DP044-3-E-F1 | 27,679 | 433 | 0.9991 | 4.85 | 0.0185 | 447 |
| DP044-3-E-F2 | 27,679 | 426 | 0.9974 | 4.01 | 0.0629 | 477 |
| DP044-3-E-F3 | 27,679 | 434 | 0.9971 | 4.41 | 0.024 | 498 |
| DP044-3-E-M1 | 27,679 | 413 | 0.997 | 3.77 | 0.0657 | 485 |
| DP044-3-E-M2 | 27,679 | 434 | 0.9977 | 4.51 | 0.0242 | 481 |
| DP044-3-E-M3 | 27,679 | 441 | 0.998 | 4.57 | 0.0295 | 472 |
| DP044-10-C-M1 | 27,679 | 448 | 0.9969 | 3.91 | 0.0516 | 526 |
| DP044-10-C-M2 | 27,679 | 426 | 0.9964 | 3.37 | 0.0872 | 507 |
| DP044-10-C-M3 | 27,679 | 404 | 0.9968 | 3.63 | 0.0609 | 489 |
| DP044-10-E-F1 | 27,679 | 503 | 0.9972 | 4.84 | 0.0194 | 582 |
| DP044-10-E-F2 | 27,679 | 458 | 0.9974 | 4.25 | 0.042 | 514 |
| DP044-10-E-F3 | 27,679 | 465 | 0.9965 | 4.23 | 0.0387 | 551 |
| DP044-10-E-M1 | 27,679 | 382 | 0.9973 | 3.81 | 0.066 | 452 |
| DP044-10-E-M2 | 27,679 | 448 | 0.9977 | 4.34 | 0.0365 | 489 |
| DP044-10-E-M3 | 27,679 | 507 | 0.9971 | 4.6 | 0.0246 | 580 |
| DP046-3-C-F1 | 27,679 | 512 | 0.9968 | 4.55 | 0.024 | 584 |
| DP046-3-C-F2 | 27,679 | 539 | 0.9961 | 4.18 | 0.0614 | 634 |
| DP046-3-C-M1 | 27,679 | 431 | 0.9965 | 3.58 | 0.0895 | 521 |
| DP046-3-C-M2 | 27,679 | 420 | 0.9967 | 3.53 | 0.0908 | 501 |
| DP046-3-C-M3 | 27,679 | 482 | 0.9955 | 4.02 | 0.0458 | 632 |
| DP046-3-E-F1 | 27,679 | 383 | 0.9975 | 3.84 | 0.0489 | 429 |
| DP046-3-E-F2 | 27,679 | 376 | 0.9965 | 3 | 0.1305 | 467 |
| DP046-3-E-F3 | 27,679 | 460 | 0.9973 | 4.63 | 0.0195 | 519 |
| DP046-3-E-M1 | 27,679 | 372 | 0.9979 | 3.81 | 0.0924 | 421 |
| DP046-3-E-M2 | 27,679 | 280 | 0.9973 | 2.76 | 0.1603 | 353 |
| DP046-3-E-M3 | 27,679 | 330 | 0.997 | 3.48 | 0.0634 | 420 |
| DP046-10-C-F2 | 27,679 | 374 | 0.9967 | 2.52 | 0.2376 | 440 |
| DP046-10-C-F3 | 27,679 | 500 | 0.9963 | 3.36 | 0.1749 | 597 |
| DP046-10-C-M1 | 27,679 | 433 | 0.9969 | 3.77 | 0.078 | 498 |
| DP046-10-C-M2 | 27,679 | 423 | 0.9966 | 3.57 | 0.0879 | 506 |
| DP046-10-C-M3 | 27,679 | 488 | 0.9963 | 4.02 | 0.0523 | 578 |
| DP046-10-E-F1 | 27,679 | 390 | 0.997 | 3.76 | 0.0495 | 480 |
| DP046-10-E-F2 | 27,679 | 405 | 0.9975 | 3.69 | 0.0669 | 444 |
| DP046-10-E-F3 | 27,679 | 396 | 0.997 | 3.72 | 0.0586 | 471 |
| DP046-10-E-M1 | 27,679 | 444 | 0.9979 | 4.78 | 0.0158 | 507 |
| DP046-10-E-M2 | 27,679 | 443 | 0.9978 | 4.67 | 0.0184 | 482 |
| DP046-10-E-M3 | 27,679 | 437 | 0.997 | 4.55 | 0.0224 | 538 |
| DP047-3-C-F1 | 27,679 | 431 | 0.9968 | 3.52 | 0.0933 | 499 |
| DP047-3-C-F2 | 27,679 | 400 | 0.9964 | 3.29 | 0.1115 | 486 |
| DP047-3-C-F3 | 27,679 | 420 | 0.9962 | 3.46 | 0.1072 | 517 |
| DP047-3-C-M1 | 27,679 | 390 | 0.9961 | 3.41 | 0.0829 | 490 |
| DP047-3-C-M2 | 27,679 | 349 | 0.9964 | 2.95 | 0.1311 | 478 |
| DP047-3-C-M3 | 27,679 | 385 | 0.9967 | 3.05 | 0.1194 | 461 |
| DP047-3-E-F1 | 27,679 | 469 | 0.9977 | 4.62 | 0.0256 | 513 |
| DP047-3-E-F2 | 27,679 | 391 | 0.9972 | 3.93 | 0.0755 | 475 |
| DP047-3-E-F3 | 27,679 | 447 | 0.9973 | 4.49 | 0.0277 | 506 |
| DP047-3-E-M1 | 27,679 | 455 | 0.9975 | 4.51 | 0.026 | 500 |
| DP047-3-E-M2 | 27,679 | 375 | 0.9974 | 3.86 | 0.0512 | 419 |
| DP047-3-E-M3 | 27,679 | 442 | 0.9966 | 4.12 | 0.0367 | 523 |
| DP047-10-C-F1 | 27,679 | 298 | 0.9971 | 2.75 | 0.1964 | 386 |
| DP047-10-C-F2 | 27,679 | 504 | 0.9969 | 4.1 | 0.0753 | 573 |
| DP047-10-C-F3 | 27,679 | 507 | 0.9962 | 3.84 | 0.1 | 598 |
| DP047-10-C-M1 | 27,679 | 472 | 0.9957 | 3.63 | 0.0774 | 566 |
| DP047-10-C-M2 | 27,679 | 396 | 0.9961 | 3.07 | 0.1214 | 496 |
| DP047-10-C-M3 | 27,679 | 465 | 0.9966 | 3.58 | 0.0858 | 540 |
| DP047-10-E-F1 | 27,679 | 450 | 0.9977 | 4.23 | 0.0554 | 492 |
| DP047-10-E-F2 | 27,679 | 452 | 0.9976 | 4.69 | 0.0183 | 507 |
| DP047-10-E-F3 | 27,679 | 503 | 0.9976 | 4.76 | 0.0229 | 547 |
| DP047-10-E-M1 | 27,679 | 459 | 0.9977 | 4.45 | 0.0285 | 510 |
| DP047-10-E-M2 | 27,679 | 478 | 0.9969 | 4.14 | 0.0437 | 551 |
| DP047-10-E-M3 | 27,679 | 494 | 0.9967 | 4.79 | 0.0163 | 589 |
| DP048-3-C-F1 | 27,679 | 503 | 0.9975 | 4.85 | 0.015 | 548 |
| DP048-3-C-F2 | 27,679 | 435 | 0.9967 | 3.63 | 0.0734 | 522 |
| DP048-3-C-F3 | 27,679 | 418 | 0.9963 | 3.2 | 0.1189 | 499 |
| DP048-3-C-M1 | 27,679 | 397 | 0.9964 | 3.39 | 0.0849 | 489 |
| DP048-3-C-M2 | 27,679 | 503 | 0.9958 | 4.02 | 0.0473 | 639 |
| DP048-3-C-M3 | 27,679 | 415 | 0.997 | 4.04 | 0.0408 | 496 |
| DP048-3-E-F1 | 27,679 | 296 | 0.9975 | 3.33 | 0.0754 | 352 |
| DP048-3-E-F2 | 27,679 | 311 | 0.9983 | 4.19 | 0.0325 | 346 |
| DP048-3-E-F3 | 27,679 | 402 | 0.9968 | 3.78 | 0.0709 | 511 |
| DP048-3-E-M1 | 27,679 | 397 | 0.9977 | 4.35 | 0.0343 | 464 |
| DP048-3-E-M2 | 27,679 | 344 | 0.9982 | 4.49 | 0.0222 | 383 |
| DP048-3-E-M3 | 27,679 | 369 | 0.9974 | 3.46 | 0.1144 | 423 |
| DP048-10-C-F1 | 27,679 | 493 | 0.9969 | 3.45 | 0.1554 | 559 |
| DP048-10-C-F2 | 27,679 | 477 | 0.9969 | 4.06 | 0.0612 | 562 |
| DP048-10-C-F3 | 27,679 | 496 | 0.997 | 3.96 | 0.0795 | 561 |
| DP048-10-C-M1 | 27,679 | 441 | 0.9965 | 3.58 | 0.0813 | 536 |
| DP048-10-C-M2 | 27,679 | 491 | 0.9956 | 3.71 | 0.0667 | 603 |
| DP048-10-C-M3 | 27,679 | 460 | 0.997 | 4.32 | 0.0298 | 543 |
| DP048-10-E-F1 | 27,679 | 438 | 0.9969 | 4.53 | 0.021 | 548 |
| DP048-10-E-F2 | 27,679 | 431 | 0.9979 | 4.6 | 0.0215 | 471 |
| DP048-10-E-F3 | 27,679 | 364 | 0.998 | 4.48 | 0.0191 | 415 |
| DP048-10-E-M1 | 27,679 | 421 | 0.9966 | 4.47 | 0.0195 | 545 |
| DP048-10-E-M2 | 27,679 | 436 | 0.9967 | 4.42 | 0.0267 | 557 |
| DP049-3-C-F1 | 27,679 | 347 | 0.9973 | 3.37 | 0.1008 | 429 |
| DP049-3-C-F2 | 27,679 | 384 | 0.9969 | 3.48 | 0.0906 | 488 |
| DP049-3-C-F3 | 27,679 | 421 | 0.9977 | 4.17 | 0.0367 | 462 |
| DP049-3-C-M1 | 27,679 | 363 | 0.9974 | 3.32 | 0.1256 | 414 |
| DP049-3-C-M2 | 27,679 | 338 | 0.9974 | 3.62 | 0.0805 | 389 |
| DP049-3-C-M3 | 27,679 | 410 | 0.9973 | 3.84 | 0.0647 | 481 |
| DP049-3-E-F1 | 27,679 | 435 | 0.9975 | 4.19 | 0.0537 | 492 |
| DP049-3-E-F2 | 27,679 | 417 | 0.9978 | 4.47 | 0.0228 | 467 |
| DP049-3-E-F3 | 27,679 | 288 | 0.9975 | 3.47 | 0.0726 | 383 |
| DP049-3-E-M1 | 27,679 | 390 | 0.9973 | 3.81 | 0.0541 | 456 |
| DP049-3-E-M2 | 27,679 | 382 | 0.9974 | 3.51 | 0.0969 | 441 |
| DP049-3-E-M3 | 27,679 | 276 | 0.9973 | 3.25 | 0.0776 | 349 |
| DP049-10-C-F1 | 27,679 | 428 | 0.9973 | 4.49 | 0.0346 | 494 |
| DP049-10-C-F2 | 27,679 | 327 | 0.9968 | 3.19 | 0.0891 | 412 |
| DP049-10-C-F3 | 27,679 | 439 | 0.9975 | 4.38 | 0.0315 | 506 |
| DP049-10-C-M1 | 27,679 | 365 | 0.9971 | 3.66 | 0.0695 | 433 |
| DP049-10-C-M2 | 27,679 | 365 | 0.9968 | 3.83 | 0.0495 | 468 |
| DP049-10-C-M3 | 27,679 | 439 | 0.9969 | 4.05 | 0.0465 | 520 |
| DP049-10-E-F1 | 27,679 | 322 | 0.9977 | 3.9 | 0.0507 | 387 |
| DP049-10-E-F2 | 27,679 | 417 | 0.9969 | 4.16 | 0.0373 | 498 |
| DP049-10-E-F3 | 27,679 | 388 | 0.9975 | 4 | 0.0454 | 435 |
| DP049-10-E-M1 | 27,679 | 378 | 0.9973 | 3.35 | 0.0995 | 455 |
| DP049-10-E-M2 | 27,679 | 364 | 0.997 | 2.96 | 0.1614 | 451 |
| DP049-10-E-M3 | 27,679 | 351 | 0.9976 | 3.28 | 0.0989 | 406 |
| DP050-3-C-F1 | 27,679 | 372 | 0.9967 | 3.14 | 0.1084 | 453 |
| DP050-3-C-F2 | 27,679 | 481 | 0.9968 | 3.88 | 0.0557 | 536 |
| DP050-3-C-F3 | 27,679 | 386 | 0.9964 | 3.04 | 0.124 | 467 |
| DP050-3-C-M1 | 27,679 | 477 | 0.9969 | 4.25 | 0.0356 | 546 |
| DP050-3-C-M2 | 27,679 | 548 | 0.9966 | 4.77 | 0.0189 | 658 |
| DP050-3-C-M3 | 27,679 | 358 | 0.9966 | 3.53 | 0.0626 | 486 |
| DP050-3-E-F1 | 27,679 | 448 | 0.9973 | 4.25 | 0.0532 | 537 |
| DP050-3-E-F2 | 27,679 | 443 | 0.9961 | 3.44 | 0.0862 | 561 |
| DP050-3-E-F3 | 27,679 | 493 | 0.9971 | 4.55 | 0.0282 | 553 |
| DP050-3-E-M1 | 27,679 | 460 | 0.9969 | 4.1 | 0.0413 | 531 |
| DP050-3-E-M2 | 27,679 | 448 | 0.9971 | 4.57 | 0.0264 | 525 |
| DP050-3-E-M3 | 27,679 | 444 | 0.9974 | 4.51 | 0.0225 | 502 |
| DP050-10-C-F1 | 27,679 | 527 | 0.9973 | 4.5 | 0.0354 | 587 |
| DP050-10-C-F2 | 27,679 | 522 | 0.997 | 4.77 | 0.0184 | 594 |
| DP050-10-C-F3 | 27,679 | 517 | 0.9972 | 4.69 | 0.0225 | 592 |
| DP050-10-C-M1 | 27,679 | 502 | 0.9966 | 4.39 | 0.0303 | 568 |
| DP050-10-C-M2 | 27,679 | 545 | 0.9962 | 4.76 | 0.0183 | 673 |
| DP050-10-C-M3 | 27,679 | 442 | 0.9971 | 4.01 | 0.0427 | 502 |
| DP050-10-E-F1 | 27,679 | 470 | 0.9953 | 2.87 | 0.1739 | 587 |
| DP050-10-E-F2 | 27,679 | 477 | 0.9946 | 3.02 | 0.1447 | 652 |
| DP050-10-E-F3 | 27,679 | 429 | 0.9963 | 3.28 | 0.1022 | 532 |
| DP050-10-E-M1 | 27,679 | 441 | 0.9971 | 4.25 | 0.047 | 520 |
| DP050-10-E-M2 | 27,679 | 475 | 0.9983 | 4.88 | 0.016 | 499 |
| DP050-10-E-M3 | 27,679 | 527 | 0.996 | 4.56 | 0.0247 | 636 |
| DP051-3-C-F1 | 27,679 | 387 | 0.9963 | 3.26 | 0.0962 | 484 |
| DP051-3-C-F2 | 27,679 | 428 | 0.9958 | 3.52 | 0.0714 | 559 |
| DP051-3-C-F3 | 27,679 | 383 | 0.9969 | 3.18 | 0.1233 | 449 |
| DP051-3-C-M1 | 27,679 | 375 | 0.9961 | 3.31 | 0.0902 | 491 |
| DP051-3-C-M2 | 27,679 | 421 | 0.9959 | 3.4 | 0.0854 | 536 |
| DP051-3-C-M3 | 27,679 | 405 | 0.9964 | 3.36 | 0.0863 | 490 |
| DP051-3-E-F1 | 27,679 | 499 | 0.9968 | 4.19 | 0.0472 | 559 |
| DP051-3-E-F2 | 27,679 | 454 | 0.9965 | 4.2 | 0.0348 | 535 |
| DP051-3-E-F3 | 27,679 | 505 | 0.997 | 4.64 | 0.0245 | 563 |
| DP051-3-E-M1 | 27,679 | 493 | 0.9969 | 4.03 | 0.0506 | 559 |
| DP051-3-E-M2 | 27,679 | 457 | 0.9965 | 3.82 | 0.0567 | 537 |
| DP051-3-E-M3 | 27,679 | 502 | 0.9958 | 3.89 | 0.0622 | 613 |
| DP051-10-C-F1 | 27,679 | 418 | 0.9967 | 3.39 | 0.1066 | 520 |
| DP051-10-C-F2 | 27,679 | 452 | 0.9972 | 4 | 0.0513 | 533 |
| DP051-10-C-F3 | 27,679 | 373 | 0.9967 | 2.9 | 0.169 | 449 |
| DP051-10-C-M1 | 27,679 | 412 | 0.9955 | 3.26 | 0.0983 | 584 |
| DP051-10-C-M2 | 27,679 | 407 | 0.9961 | 3.12 | 0.108 | 516 |
| DP051-10-C-M3 | 27,679 | 490 | 0.9962 | 3.91 | 0.0534 | 606 |
| DP051-10-E-F1 | 27,679 | 420 | 0.9969 | 3.89 | 0.0497 | 498 |
| DP051-10-E-F2 | 27,679 | 445 | 0.9968 | 3.82 | 0.055 | 509 |
| DP051-10-E-F3 | 27,679 | 440 | 0.9967 | 3.77 | 0.0542 | 524 |
| DP051-10-E-M1 | 27,679 | 468 | 0.997 | 3.86 | 0.0549 | 549 |
| DP051-10-E-M2 | 27,679 | 467 | 0.9964 | 3.98 | 0.0454 | 554 |
| DP051-10-E-M3 | 27,679 | 395 | 0.9964 | 3.34 | 0.0905 | 470 |
| DP052-3-C-F1 | 27,679 | 389 | 0.9968 | 3.21 | 0.1135 | 459 |
| DP052-3-C-F2 | 27,679 | 391 | 0.9966 | 3.08 | 0.1109 | 475 |
| DP052-3-C-F3 | 27,679 | 447 | 0.9969 | 3.99 | 0.0597 | 536 |
| DP052-3-C-M1 | 27,679 | 530 | 0.996 | 4.39 | 0.0307 | 657 |
| DP052-3-C-M2 | 27,679 | 465 | 0.9963 | 3.6 | 0.1215 | 607 |
| DP052-3-C-M3 | 27,679 | 373 | 0.9974 | 3.66 | 0.085 | 431 |
| DP052-3-E-F1 | 27,679 | 296 | 0.9971 | 3.19 | 0.0955 | 398 |
| DP052-3-E-F2 | 27,679 | 353 | 0.9975 | 3.55 | 0.0877 | 429 |
| DP052-3-E-F3 | 27,679 | 363 | 0.9975 | 4.14 | 0.0438 | 449 |
| DP052-3-E-M1 | 27,679 | 231 | 0.9982 | 2.86 | 0.1189 | 271 |
| DP052-3-E-M2 | 27,679 | 337 | 0.9971 | 3.26 | 0.0863 | 399 |
| DP052-3-E-M3 | 27,679 | 312 | 0.998 | 3.92 | 0.0494 | 369 |
| DP052-10-C-F1 | 27,679 | 459 | 0.9966 | 3.52 | 0.1326 | 545 |
| DP052-10-C-F2 | 27,679 | 426 | 0.997 | 3.26 | 0.1171 | 481 |
| DP052-10-C-F3 | 27,679 | 406 | 0.996 | 3.16 | 0.1353 | 511 |
| DP052-10-C-M1 | 27,679 | 513 | 0.9969 | 4.16 | 0.049 | 583 |
| DP052-10-C-M2 | 27,679 | 514 | 0.9969 | 4.36 | 0.0468 | 606 |
| DP052-10-C-M3 | 27,679 | 366 | 0.9974 | 3.63 | 0.0922 | 441 |
| DP052-10-E-F1 | 27,679 | 331 | 0.9966 | 3.02 | 0.1148 | 443 |
| DP052-10-E-F2 | 27,679 | 392 | 0.9972 | 4.44 | 0.0222 | 509 |
| DP052-10-E-F3 | 27,679 | 298 | 0.9964 | 2.97 | 0.129 | 451 |
| DP052-10-E-M1 | 27,679 | 281 | 0.9979 | 3.47 | 0.0737 | 336 |
| DP052-10-E-M2 | 27,679 | 278 | 0.997 | 3.19 | 0.0852 | 373 |
| DP052-10-E-M3 | 27,679 | 336 | 0.9961 | 2.8 | 0.2097 | 485 |
| DP053-3-C-F1 | 27,679 | 445 | 0.9959 | 2.99 | 0.1925 | 558 |
| DP053-3-C-F2 | 27,679 | 414 | 0.9967 | 3.73 | 0.0676 | 492 |
| DP053-3-C-F3 | 27,679 | 344 | 0.9963 | 2.47 | 0.2711 | 456 |
| DP053-3-C-M1 | 27,679 | 523 | 0.9971 | 4.51 | 0.0385 | 587 |
| DP053-3-C-M2 | 27,679 | 531 | 0.9973 | 4.62 | 0.0243 | 583 |
| DP053-3-C-M3 | 27,679 | 515 | 0.9964 | 4.19 | 0.0429 | 602 |
| DP053-3-E-F1 | 27,679 | 319 | 0.9965 | 2.93 | 0.1268 | 427 |
| DP053-3-E-F2 | 27,679 | 347 | 0.9978 | 3.49 | 0.0919 | 393 |
| DP053-3-E-F3 | 27,679 | 423 | 0.9964 | 3.59 | 0.0861 | 510 |
| DP053-3-E-M1 | 27,679 | 359 | 0.9967 | 3.36 | 0.074 | 456 |
| DP053-3-E-M2 | 27,679 | 333 | 0.9973 | 3.23 | 0.0846 | 395 |
| DP053-3-E-M3 | 27,679 | 351 | 0.997 | 3.4 | 0.0754 | 425 |
| DP053-10-C-F1 | 27,679 | 374 | 0.9967 | 3.04 | 0.172 | 446 |
| DP053-10-C-F2 | 27,679 | 494 | 0.9972 | 4.67 | 0.0203 | 582 |
| DP053-10-C-F3 | 27,679 | 430 | 0.9964 | 3.05 | 0.1317 | 508 |
| DP053-10-C-M1 | 27,679 | 523 | 0.9969 | 4.43 | 0.0416 | 603 |
| DP053-10-C-M2 | 27,679 | 436 | 0.9964 | 3.67 | 0.0809 | 531 |
| DP053-10-C-M3 | 27,679 | 474 | 0.9957 | 3.45 | 0.1287 | 604 |
| DP053-10-E-F1 | 27,679 | 352 | 0.9971 | 3.57 | 0.0696 | 437 |
| DP053-10-E-F2 | 27,679 | 373 | 0.9975 | 4.1 | 0.0436 | 461 |
| DP053-10-E-F3 | 27,679 | 359 | 0.9974 | 4.09 | 0.0404 | 430 |
| DP053-10-E-M1 | 27,679 | 368 | 0.9974 | 4.14 | 0.0396 | 443 |
| DP053-10-E-M2 | 27,679 | 317 | 0.9977 | 3.25 | 0.0995 | 373 |
| DP053-10-E-M3 | 27,679 | 369 | 0.9974 | 4.39 | 0.0238 | 446 |
| DP054-3-C-F1 | 27,679 | 359 | 0.9965 | 3.51 | 0.0879 | 467 |
| DP054-3-C-F2 | 27,679 | 377 | 0.9967 | 2.81 | 0.2161 | 454 |
| DP054-3-C-F3 | 27,679 | 486 | 0.9973 | 4.33 | 0.0487 | 538 |
| DP054-3-C-M1 | 27,679 | 358 | 0.9965 | 2.66 | 0.2568 | 457 |
| DP054-3-C-M2 | 27,679 | 421 | 0.9966 | 3.24 | 0.088 | 485 |
| DP054-3-C-M3 | 27,679 | 398 | 0.9964 | 3.09 | 0.1486 | 490 |
| DP054-3-E-F1 | 27,679 | 270 | 0.9975 | 2.89 | 0.1196 | 332 |
| DP054-3-E-F2 | 27,679 | 309 | 0.9977 | 3.31 | 0.077 | 372 |
| DP054-3-E-F3 | 27,679 | 314 | 0.9974 | 3.37 | 0.0645 | 370 |
| DP054-3-E-M1 | 27,679 | 429 | 0.9962 | 3.57 | 0.0726 | 515 |
| DP054-3-E-M2 | 27,679 | 415 | 0.9962 | 3.68 | 0.0612 | 531 |
| DP054-3-E-M3 | 27,679 | 394 | 0.9955 | 3.46 | 0.0696 | 535 |
| DP054-10-C-F1 | 27,679 | 340 | 0.9971 | 2.87 | 0.156 | 396 |
| DP054-10-C-F2 | 27,679 | 371 | 0.9969 | 3.1 | 0.1172 | 440 |
| DP054-10-C-F3 | 27,679 | 458 | 0.9967 | 3.65 | 0.0684 | 525 |
| DP054-10-C-M1 | 27,679 | 416 | 0.9957 | 3.08 | 0.1824 | 580 |
| DP054-10-C-M2 | 27,679 | 375 | 0.9962 | 2.89 | 0.1158 | 484 |
| DP054-10-C-M3 | 27,679 | 340 | 0.9969 | 2.81 | 0.1731 | 427 |
| DP054-10-E-F1 | 27,679 | 524 | 0.9961 | 4.38 | 0.0317 | 633 |
| DP054-10-E-F2 | 27,679 | 499 | 0.9956 | 3.97 | 0.0481 | 629 |
| DP054-10-E-F3 | 27,679 | 439 | 0.9965 | 4.01 | 0.0413 | 530 |
| DP054-10-E-M1 | 27,679 | 408 | 0.997 | 4.27 | 0.0292 | 473 |
| DP054-10-E-M2 | 27,679 | 354 | 0.9973 | 3.67 | 0.0632 | 431 |
| DP054-10-E-M3 | 27,679 | 409 | 0.997 | 3.78 | 0.0679 | 486 |
| DP055-3-C-F1 | 27,679 | 474 | 0.9961 | 3.4 | 0.1354 | 575 |
| DP055-3-C-F2 | 27,679 | 511 | 0.9969 | 3.9 | 0.0768 | 573 |
| DP055-3-C-F3 | 27,679 | 432 | 0.9975 | 4.07 | 0.0539 | 490 |
| DP055-3-C-M1 | 27,679 | 459 | 0.9962 | 3.74 | 0.0739 | 549 |
| DP055-3-C-M2 | 27,679 | 340 | 0.9969 | 2.97 | 0.1492 | 412 |
| DP055-3-C-M3 | 27,679 | 394 | 0.9957 | 3.2 | 0.0953 | 535 |
| DP055-3-E-F1 | 27,679 | 345 | 0.9976 | 3.73 | 0.0584 | 398 |
| DP055-3-E-F2 | 27,679 | 380 | 0.9967 | 3.58 | 0.0648 | 465 |
| DP055-3-E-F3 | 27,679 | 436 | 0.9975 | 4.76 | 0.0165 | 509 |
| DP055-3-E-M1 | 27,679 | 400 | 0.9974 | 4.54 | 0.0218 | 482 |
| DP055-3-E-M2 | 27,679 | 387 | 0.9965 | 3.7 | 0.0596 | 504 |
| DP055-3-E-M3 | 27,679 | 396 | 0.9976 | 4.41 | 0.0338 | 481 |
| DP055-10-C-F1 | 27,679 | 358 | 0.997 | 2.91 | 0.1357 | 427 |
| DP055-10-C-F2 | 27,679 | 362 | 0.997 | 2.98 | 0.1382 | 424 |
| DP055-10-C-F3 | 27,679 | 398 | 0.9968 | 3.22 | 0.1091 | 471 |
| DP055-10-C-M1 | 27,679 | 411 | 0.9967 | 3.11 | 0.1509 | 480 |
| DP055-10-C-M2 | 27,679 | 419 | 0.9959 | 3.4 | 0.1043 | 541 |
| DP055-10-C-M3 | 27,679 | 504 | 0.9964 | 3.58 | 0.0966 | 584 |
| DP055-10-E-F1 | 27,679 | 256 | 0.9978 | 3.62 | 0.0517 | 317 |
| DP055-10-E-F2 | 27,679 | 362 | 0.9971 | 3.56 | 0.0723 | 426 |
| DP055-10-E-F3 | 27,679 | 408 | 0.9969 | 4.25 | 0.0288 | 493 |
| DP055-10-E-M1 | 27,679 | 449 | 0.9969 | 4.47 | 0.027 | 560 |
| DP055-10-E-M2 | 27,679 | 413 | 0.9969 | 3.56 | 0.0918 | 485 |
| DP055-10-E-M3 | 27,679 | 362 | 0.9971 | 3.7 | 0.0669 | 439 |
| DP056-3-C-F1 | 27,679 | 549 | 0.9961 | 4.25 | 0.0527 | 648 |
| DP056-3-C-F2 | 27,679 | 337 | 0.9967 | 3.33 | 0.0778 | 424 |
| DP056-3-C-F3 | 27,679 | 528 | 0.9967 | 4.15 | 0.0604 | 607 |
| DP056-3-C-M1 | 27,679 | 482 | 0.9961 | 3.76 | 0.0674 | 593 |
| DP056-3-C-M2 | 27,679 | 494 | 0.9959 | 3.78 | 0.0647 | 611 |
| DP056-3-C-M3 | 27,679 | 520 | 0.9953 | 3.91 | 0.0568 | 660 |
| DP056-3-E-F1 | 27,679 | 328 | 0.9982 | 3.95 | 0.048 | 368 |
| DP056-3-E-F2 | 27,679 | 294 | 0.9966 | 2.82 | 0.1358 | 447 |
| DP056-3-E-F3 | 27,679 | 325 | 0.9983 | 3.95 | 0.0464 | 359 |
| DP056-3-E-M1 | 27,679 | 460 | 0.9962 | 3.69 | 0.0693 | 558 |
| DP056-3-E-M2 | 27,679 | 518 | 0.9965 | 3.84 | 0.1143 | 602 |
| DP056-3-E-M3 | 27,679 | 520 | 0.9967 | 3.69 | 0.1448 | 588 |
| DP056-10-C-F1 | 27,679 | 343 | 0.9971 | 3.76 | 0.0481 | 410 |
| DP056-10-C-F2 | 27,679 | 449 | 0.9967 | 3.79 | 0.0604 | 521 |
| DP056-10-C-F3 | 27,679 | 531 | 0.9962 | 4.55 | 0.0245 | 627 |
| DP056-10-C-M1 | 27,679 | 381 | 0.9971 | 3.76 | 0.0522 | 442 |
| DP056-10-C-M2 | 27,679 | 313 | 0.9971 | 3.61 | 0.0579 | 386 |
| DP056-10-C-M3 | 27,679 | 327 | 0.9969 | 3.57 | 0.0573 | 403 |
| DP056-10-E-F1 | 27,679 | 326 | 0.9969 | 3.25 | 0.086 | 402 |
| DP056-10-E-F2 | 27,679 | 413 | 0.9973 | 4.5 | 0.0256 | 494 |
| DP056-10-E-F3 | 27,679 | 363 | 0.9973 | 3.92 | 0.0444 | 429 |
| DP056-10-E-M1 | 27,679 | 422 | 0.9967 | 3.88 | 0.0522 | 504 |
| DP056-10-E-M2 | 27,679 | 494 | 0.9948 | 3.76 | 0.06 | 666 |
| DP056-10-E-M3 | 27,679 | 477 | 0.9957 | 3.79 | 0.0548 | 610 |
| DP057-3-C-F1 | 27,679 | 307 | 0.9969 | 2.89 | 0.1059 | 388 |
| DP057-3-C-F2 | 27,679 | 424 | 0.9956 | 3.09 | 0.1213 | 578 |
| DP057-3-C-F3 | 27,679 | 336 | 0.9964 | 2.59 | 0.1745 | 441 |
| DP057-3-C-M1 | 27,679 | 484 | 0.9959 | 3.41 | 0.1295 | 599 |
| DP057-3-C-M2 | 27,679 | 278 | 0.9966 | 2.17 | 0.2043 | 377 |
| DP057-3-C-M3 | 27,679 | 471 | 0.9961 | 3.54 | 0.0957 | 580 |
| DP057-3-E-F1 | 27,679 | 407 | 0.9961 | 3.27 | 0.0826 | 500 |
| DP057-3-E-F2 | 27,679 | 333 | 0.997 | 2.54 | 0.2494 | 433 |
| DP057-3-E-F3 | 27,679 | 269 | 0.997 | 2.61 | 0.1395 | 361 |
| DP057-3-E-M1 | 27,679 | 312 | 0.9965 | 3.38 | 0.0719 | 441 |
| DP057-3-E-M2 | 27,679 | 246 | 0.9974 | 2.97 | 0.111 | 319 |
| DP057-3-E-M3 | 27,679 | 388 | 0.9963 | 3.23 | 0.0954 | 485 |
| DP057-10-C-F1 | 27,679 | 523 | 0.9968 | 4.9 | 0.0156 | 626 |
| DP057-10-C-F2 | 27,679 | 385 | 0.9962 | 2.56 | 0.2914 | 503 |
| DP057-10-C-F3 | 27,679 | 349 | 0.9969 | 3.42 | 0.0746 | 425 |
| DP057-10-C-M1 | 27,679 | 430 | 0.996 | 3.42 | 0.0817 | 532 |
| DP057-10-C-M2 | 27,679 | 499 | 0.997 | 4.32 | 0.037 | 562 |
| DP057-10-C-M3 | 27,679 | 517 | 0.9966 | 4.52 | 0.0257 | 594 |
| DP057-10-E-F1 | 27,679 | 327 | 0.9972 | 3.52 | 0.0637 | 387 |
| DP057-10-E-F2 | 27,679 | 182 | 0.998 | 2.77 | 0.1163 | 244 |
| DP057-10-E-F3 | 27,679 | 330 | 0.9969 | 2.95 | 0.1326 | 417 |
| DP057-10-E-M1 | 27,679 | 339 | 0.9965 | 3.63 | 0.0492 | 445 |
| DP057-10-E-M2 | 27,679 | 410 | 0.9965 | 4.04 | 0.0351 | 482 |
| DP057-10-E-M3 | 27,679 | 439 | 0.9959 | 3.99 | 0.0396 | 543 |
| DP059-3-C-F1 | 27,679 | 415 | 0.9961 | 2.91 | 0.1968 | 511 |
| DP059-3-C-F2 | 27,679 | 358 | 0.9965 | 2.22 | 0.3721 | 436 |
| DP059-3-C-F3 | 27,679 | 173 | 0.9976 | 1.54 | 0.4441 | 262 |
| DP059-3-C-M1 | 27,679 | 430 | 0.9961 | 3.2 | 0.0948 | 531 |
| DP059-3-C-M2 | 27,679 | 525 | 0.9969 | 3.88 | 0.0729 | 584 |
| DP059-3-C-M3 | 27,679 | 450 | 0.9962 | 3.41 | 0.1056 | 564 |
| DP059-3-E-F1 | 27,679 | 336 | 0.9975 | 3.39 | 0.1058 | 407 |
| DP059-3-E-F2 | 27,679 | 275 | 0.9977 | 3.09 | 0.0973 | 326 |
| DP059-3-E-F3 | 27,679 | 271 | 0.9984 | 3.63 | 0.0531 | 299 |
| DP059-3-E-M1 | 27,679 | 408 | 0.9966 | 3.72 | 0.0614 | 520 |
| DP059-3-E-M2 | 27,679 | 405 | 0.9974 | 3.87 | 0.0526 | 461 |
| DP059-3-E-M3 | 27,679 | 396 | 0.9972 | 4.09 | 0.0403 | 469 |
| DP059-10-C-F1 | 27,679 | 414 | 0.9957 | 2.45 | 0.285 | 546 |
| DP059-10-C-F2 | 27,679 | 408 | 0.9961 | 2.82 | 0.2267 | 493 |
| DP059-10-C-F3 | 27,679 | 234 | 0.9975 | 2.37 | 0.1785 | 307 |
| DP059-10-C-M1 | 27,679 | 452 | 0.9966 | 3.38 | 0.1095 | 518 |
| DP059-10-C-M2 | 27,679 | 414 | 0.996 | 3.29 | 0.0886 | 519 |
| DP059-10-C-M3 | 27,679 | 528 | 0.9966 | 4.44 | 0.0384 | 617 |
| DP059-10-E-F1 | 27,679 | 290 | 0.9969 | 3.29 | 0.0823 | 384 |
| DP059-10-E-F2 | 27,679 | 301 | 0.9969 | 3.51 | 0.058 | 422 |
| DP059-10-E-F3 | 27,679 | 295 | 0.9981 | 3.89 | 0.0376 | 331 |
| DP059-10-E-M1 | 27,679 | 321 | 0.9976 | 3.13 | 0.108 | 382 |
| DP059-10-E-M2 | 27,679 | 267 | 0.9975 | 2.77 | 0.1594 | 322 |
| DP059-10-E-M3 | 27,679 | 294 | 0.9974 | 2.58 | 0.2151 | 351 |
| DP060-3-C-F1 | 27,679 | 301 | 0.9966 | 2.58 | 0.2003 | 398 |
| DP060-3-C-F2 | 27,679 | 201 | 0.9975 | 1.99 | 0.3221 | 287 |
| DP060-3-C-F3 | 27,679 | 215 | 0.9977 | 2.24 | 0.3069 | 287 |
| DP060-3-C-M1 | 27,679 | 428 | 0.9962 | 3.94 | 0.045 | 522 |
| DP060-3-C-M2 | 27,679 | 479 | 0.9961 | 3.64 | 0.0853 | 564 |
| DP060-3-C-M3 | 27,679 | 420 | 0.9963 | 2.79 | 0.1965 | 514 |
| DP060-3-E-F1 | 27,679 | 364 | 0.9976 | 3.72 | 0.06 | 419 |
| DP060-3-E-F2 | 27,679 | 303 | 0.9976 | 3.8 | 0.0441 | 364 |
| DP060-3-E-F3 | 27,679 | 393 | 0.9972 | 3.96 | 0.0499 | 455 |
| DP060-3-E-M1 | 27,679 | 281 | 0.9971 | 3.32 | 0.0731 | 353 |
| DP060-3-E-M2 | 27,679 | 306 | 0.9969 | 3.76 | 0.046 | 447 |
| DP060-3-E-M3 | 27,679 | 374 | 0.9967 | 3.76 | 0.051 | 450 |
| DP060-10-C-F1 | 27,679 | 224 | 0.9973 | 2.35 | 0.2276 | 341 |
| DP060-10-C-F2 | 27,679 | 190 | 0.9978 | 1.94 | 0.3019 | 245 |
| DP060-10-C-F3 | 27,679 | 311 | 0.9967 | 2.51 | 0.2539 | 404 |
| DP060-10-C-M1 | 27,679 | 482 | 0.9964 | 3.7 | 0.0814 | 562 |
| DP060-10-C-M2 | 27,679 | 488 | 0.9963 | 4.12 | 0.0471 | 579 |
| DP060-10-C-M3 | 27,679 | 494 | 0.9964 | 3.53 | 0.1261 | 573 |
| DP060-10-E-F1 | 27,679 | 404 | 0.9972 | 4.15 | 0.0598 | 477 |
| DP060-10-E-F2 | 27,679 | 387 | 0.9975 | 3.78 | 0.0994 | 441 |
| DP060-10-E-F3 | 27,679 | 329 | 0.9974 | 3.57 | 0.0686 | 395 |
| DP060-10-E-M1 | 27,679 | 451 | 0.9969 | 4.24 | 0.036 | 512 |
| DP060-10-E-M2 | 27,679 | 565 | 0.994 | 4.5 | 0.0218 | 782 |
| DP060-10-E-M3 | 27,679 | 457 | 0.997 | 4.14 | 0.0414 | 517 |
| DP061-3-C-F1 | 27,679 | 192 | 0.9977 | 1.67 | 0.4342 | 270 |
| DP061-3-C-F2 | 27,679 | 424 | 0.996 | 2.84 | 0.2492 | 541 |
| DP061-3-C-F3 | 27,679 | 216 | 0.9977 | 2.12 | 0.3463 | 288 |
| DP061-3-C-M1 | 27,679 | 519 | 0.9958 | 4.22 | 0.0456 | 630 |
| DP061-3-C-M2 | 27,679 | 457 | 0.9963 | 3.37 | 0.1272 | 558 |
| DP061-3-C-M3 | 27,679 | 483 | 0.996 | 3.26 | 0.1513 | 592 |
| DP061-3-E-F1 | 27,679 | 437 | 0.9965 | 4.09 | 0.037 | 546 |
| DP061-3-E-F2 | 27,679 | 438 | 0.9974 | 4.58 | 0.0222 | 509 |
| DP061-3-E-F3 | 27,679 | 439 | 0.9974 | 4.49 | 0.0401 | 512 |
| DP061-3-E-M1 | 27,679 | 400 | 0.9967 | 3.65 | 0.0723 | 470 |
| DP061-3-E-M2 | 27,679 | 423 | 0.9971 | 3.55 | 0.1333 | 496 |
| DP061-3-E-M3 | 27,679 | 368 | 0.997 | 3.88 | 0.0443 | 437 |
| DP061-10-C-F1 | 27,679 | 246 | 0.9974 | 1.88 | 0.3618 | 315 |
| DP061-10-C-F2 | 27,679 | 246 | 0.9972 | 1.78 | 0.4488 | 332 |
| DP061-10-C-F3 | 27,679 | 272 | 0.9964 | 2.51 | 0.1416 | 369 |
| DP061-10-C-M1 | 27,679 | 467 | 0.9969 | 3.42 | 0.1411 | 535 |
| DP061-10-C-M2 | 27,679 | 388 | 0.9962 | 2.7 | 0.2042 | 493 |
| DP061-10-C-M3 | 27,679 | 517 | 0.9965 | 3.74 | 0.0941 | 589 |
| DP061-10-E-F1 | 27,679 | 444 | 0.9969 | 4.7 | 0.019 | 552 |
| DP061-10-E-F2 | 27,679 | 461 | 0.9972 | 4.41 | 0.0393 | 534 |
| DP061-10-E-F3 | 27,679 | 468 | 0.9971 | 4.67 | 0.0177 | 536 |
| DP061-10-E-M1 | 27,679 | 514 | 0.9966 | 4.72 | 0.0184 | 603 |
| DP061-10-E-M2 | 27,679 | 492 | 0.9964 | 3.93 | 0.0598 | 585 |
| DP061-10-E-M3 | 27,679 | 515 | 0.9964 | 4.04 | 0.0818 | 596 |
| DP062-3-C-F1 | 27,679 | 508 | 0.9965 | 4.49 | 0.0285 | 598 |
| DP062-3-C-F2 | 27,679 | 453 | 0.9972 | 4.33 | 0.0348 | 510 |
| DP062-3-C-F3 | 27,679 | 498 | 0.9962 | 4.44 | 0.0337 | 599 |
| DP062-3-C-F4 | 27,679 | 367 | 0.9966 | 3.89 | 0.0447 | 483 |
| DP062-3-C-F5 | 27,679 | 303 | 0.9975 | 3.55 | 0.0663 | 366 |
| DP062-3-E-F1 | 27,679 | 438 | 0.9968 | 4.52 | 0.0224 | 518 |
| DP062-3-E-F2 | 27,679 | 373 | 0.9977 | 4.12 | 0.047 | 436 |
| DP062-3-E-F3 | 27,679 | 418 | 0.997 | 4.3 | 0.0307 | 489 |
| DP062-3-E-M1 | 27,679 | 375 | 0.9966 | 3.68 | 0.0689 | 479 |
| DP062-3-E-M2 | 27,679 | 369 | 0.9973 | 4.05 | 0.0365 | 437 |
| DP062-10-C-F1 | 27,679 | 495 | 0.996 | 4.41 | 0.0304 | 631 |
| DP062-10-C-F2 | 27,679 | 436 | 0.9969 | 4.09 | 0.0454 | 502 |
| DP062-10-C-F3 | 27,679 | 469 | 0.9965 | 4.18 | 0.0426 | 580 |
| DP062-10-C-F4 | 27,679 | 367 | 0.9971 | 3.89 | 0.0459 | 444 |
| DP062-10-C-F5 | 27,679 | 293 | 0.9976 | 3.38 | 0.0806 | 358 |
| DP062-10-E-F1 | 27,679 | 328 | 0.9975 | 3.44 | 0.0746 | 388 |
| DP062-10-E-F2 | 27,679 | 303 | 0.9973 | 3.27 | 0.0824 | 378 |
| DP062-10-E-F3 | 27,679 | 321 | 0.9974 | 3.45 | 0.0798 | 375 |
| DP062-10-E-M1 | 27,679 | 354 | 0.9972 | 3.72 | 0.0526 | 416 |
| DP062-10-E-M2 | 27,679 | 312 | 0.9974 | 3.16 | 0.1079 | 385 |
| DP063-3-C-F1 | 27,679 | 267 | 0.9971 | 2.48 | 0.2945 | 380 |
| DP063-3-C-F2 | 27,679 | 166 | 0.9979 | 1.62 | 0.4352 | 241 |
| DP063-3-C-F3 | 27,679 | 193 | 0.9978 | 1.82 | 0.4008 | 297 |
| DP063-3-C-M1 | 27,679 | 533 | 0.9964 | 3.65 | 0.1135 | 618 |
| DP063-3-C-M2 | 27,679 | 500 | 0.9963 | 3.31 | 0.169 | 578 |
| DP063-3-C-M3 | 27,679 | 488 | 0.9966 | 3.56 | 0.1196 | 555 |
| DP063-3-E-F1 | 27,679 | 376 | 0.997 | 3.62 | 0.0698 | 455 |
| DP063-3-E-F2 | 27,679 | 279 | 0.9975 | 3.2 | 0.1041 | 352 |
| DP063-3-E-F3 | 27,679 | 221 | 0.998 | 2.62 | 0.1313 | 288 |
| DP063-3-E-M1 | 27,679 | 447 | 0.9969 | 4.13 | 0.0468 | 521 |
| DP063-3-E-M2 | 27,679 | 425 | 0.9966 | 3 | 0.2567 | 513 |
| DP063-3-E-M3 | 27,679 | 438 | 0.9966 | 3.9 | 0.0482 | 517 |
| DP063-10-C-F1 | 27,679 | 349 | 0.9964 | 2.79 | 0.2561 | 482 |
| DP063-10-C-F2 | 27,679 | 120 | 0.9988 | 1.52 | 0.4168 | 147 |
| DP063-10-C-F3 | 27,679 | 168 | 0.9977 | 1.72 | 0.4082 | 298 |
| DP063-10-C-M1 | 27,679 | 492 | 0.9966 | 3.57 | 0.1185 | 556 |
| DP063-10-C-M2 | 27,679 | 518 | 0.9963 | 3.68 | 0.1161 | 612 |
| DP063-10-C-M3 | 27,679 | 373 | 0.9958 | 2.54 | 0.275 | 516 |
| DP063-10-E-F1 | 27,679 | 420 | 0.9974 | 4.3 | 0.0356 | 482 |
| DP063-10-E-F2 | 27,679 | 260 | 0.9973 | 3.41 | 0.0768 | 353 |
| DP063-10-E-F3 | 27,679 | 319 | 0.9978 | 3.87 | 0.0465 | 357 |
| DP063-10-E-M1 | 27,679 | 361 | 0.9973 | 3.8 | 0.074 | 438 |
| DP063-10-E-M2 | 27,679 | 447 | 0.9971 | 4.23 | 0.0391 | 521 |
| DP063-10-E-M3 | 27,679 | 451 | 0.9965 | 3.36 | 0.1572 | 539 |
| DP064-3-C-F1 | 27,679 | 457 | 0.9972 | 4.12 | 0.0511 | 516 |
| DP064-3-C-F2 | 27,679 | 514 | 0.9971 | 4.38 | 0.0283 | 563 |
| DP064-3-C-F3 | 27,679 | 406 | 0.9964 | 3.22 | 0.1016 | 486 |
| DP064-3-C-M1 | 27,679 | 501 | 0.9958 | 3.46 | 0.1262 | 624 |
| DP064-3-C-M2 | 27,679 | 481 | 0.996 | 3.24 | 0.1299 | 578 |
| DP064-3-C-M3 | 27,679 | 501 | 0.9963 | 3.66 | 0.1218 | 598 |
| DP064-3-E-F1 | 27,679 | 462 | 0.9964 | 4.07 | 0.0421 | 532 |
| DP064-3-E-F2 | 27,679 | 514 | 0.9964 | 4.38 | 0.0354 | 604 |
| DP064-3-E-F3 | 27,679 | 465 | 0.9963 | 4.03 | 0.0477 | 574 |
| DP064-3-E-M1 | 27,679 | 424 | 0.9953 | 3.66 | 0.0601 | 588 |
| DP064-3-E-M2 | 27,679 | 499 | 0.9961 | 4.28 | 0.0331 | 606 |
| DP064-3-E-M3 | 27,679 | 495 | 0.9961 | 3.97 | 0.0481 | 583 |
| DP064-10-C-F1 | 27,679 | 437 | 0.9973 | 3.6 | 0.0889 | 482 |
| DP064-10-C-F2 | 27,679 | 468 | 0.9964 | 3.81 | 0.0513 | 549 |
| DP064-10-C-F3 | 27,679 | 295 | 0.9969 | 2.76 | 0.1442 | 374 |
| DP064-10-C-M1 | 27,679 | 466 | 0.9955 | 3.37 | 0.1254 | 575 |
| DP064-10-C-M2 | 27,679 | 448 | 0.9962 | 3.16 | 0.1511 | 555 |
| DP064-10-C-M3 | 27,679 | 403 | 0.996 | 3.12 | 0.126 | 500 |
| DP064-10-E-F1 | 27,679 | 518 | 0.9963 | 4.29 | 0.0515 | 607 |
| DP064-10-E-F2 | 27,679 | 430 | 0.9973 | 3.74 | 0.0849 | 503 |
| DP064-10-E-F3 | 27,679 | 525 | 0.9966 | 4.4 | 0.0356 | 613 |
| DP064-10-E-M1 | 27,679 | 510 | 0.9968 | 4.63 | 0.0261 | 603 |
| DP064-10-E-M2 | 27,679 | 549 | 0.9964 | 4.28 | 0.0447 | 632 |
| DP064-10-E-M3 | 27,679 | 509 | 0.9958 | 4.05 | 0.0561 | 628 |
| DP065-3-C-F1 | 27,679 | 259 | 0.9973 | 2.57 | 0.1625 | 346 |
| DP065-3-C-F2 | 27,679 | 319 | 0.9974 | 3.3 | 0.0982 | 429 |
| DP065-3-C-F3 | 27,679 | 301 | 0.9972 | 3.08 | 0.1402 | 408 |
| DP065-3-C-M1 | 27,679 | 348 | 0.997 | 3.03 | 0.1564 | 451 |
| DP065-3-C-M2 | 27,679 | 264 | 0.997 | 2.84 | 0.1413 | 356 |
| DP065-3-C-M3 | 27,679 | 225 | 0.9973 | 2.91 | 0.0954 | 320 |
| DP065-3-E-F1 | 27,679 | 256 | 0.9969 | 2.9 | 0.1224 | 350 |
| DP065-3-E-F2 | 27,679 | 251 | 0.9972 | 2.35 | 0.1951 | 345 |
| DP065-3-E-F3 | 27,679 | 308 | 0.9971 | 3.27 | 0.0845 | 375 |
| DP065-3-E-M1 | 27,679 | 354 | 0.9966 | 3.56 | 0.0572 | 440 |
| DP065-3-E-M2 | 27,679 | 284 | 0.997 | 2.73 | 0.1509 | 379 |
| DP065-3-E-M3 | 27,679 | 339 | 0.9956 | 3.11 | 0.0933 | 492 |
| DP065-10-C-F1 | 27,679 | 359 | 0.9973 | 3.63 | 0.0952 | 432 |
| DP065-10-C-F2 | 27,679 | 352 | 0.9972 | 4.22 | 0.0267 | 425 |
| DP065-10-C-F3 | 27,679 | 346 | 0.9971 | 4.17 | 0.0261 | 442 |
| DP065-10-C-M1 | 27,679 | 364 | 0.996 | 3.46 | 0.0711 | 502 |
| DP065-10-C-M2 | 27,679 | 333 | 0.9965 | 3.39 | 0.0892 | 441 |
| DP065-10-C-M3 | 27,679 | 399 | 0.997 | 4.11 | 0.0374 | 502 |
| DP065-10-E-F1 | 27,679 | 247 | 0.9975 | 3.32 | 0.0719 | 312 |
| DP065-10-E-F2 | 27,679 | 262 | 0.9971 | 3.18 | 0.0837 | 337 |
| DP065-10-E-F3 | 27,679 | 262 | 0.997 | 2.96 | 0.1777 | 384 |
| DP065-10-E-M1 | 27,679 | 338 | 0.9967 | 3.47 | 0.0664 | 431 |
| DP065-10-E-M2 | 27,679 | 247 | 0.9973 | 2.94 | 0.1208 | 342 |
| DP065-10-E-M3 | 27,679 | 195 | 0.9978 | 2.13 | 0.2393 | 248 |
| DP066-3-C-F1 | 27,679 | 514 | 0.9961 | 4.64 | 0.0225 | 629 |
| DP066-3-C-F2 | 27,679 | 447 | 0.9971 | 4.54 | 0.02 | 522 |
| DP066-3-C-F3 | 27,679 | 472 | 0.9974 | 4.61 | 0.0218 | 519 |
| DP066-3-C-M1 | 27,679 | 458 | 0.9973 | 4.52 | 0.0225 | 540 |
| DP066-3-C-M2 | 27,679 | 460 | 0.9971 | 4.24 | 0.0561 | 541 |
| DP066-3-C-M3 | 27,679 | 521 | 0.9972 | 4.66 | 0.0285 | 594 |
| DP066-3-E-F1 | 27,679 | 425 | 0.9977 | 4.42 | 0.026 | 479 |
| DP066-3-E-F2 | 27,679 | 377 | 0.9967 | 3.7 | 0.0607 | 491 |
| DP066-3-E-F3 | 27,679 | 441 | 0.9964 | 3.93 | 0.046 | 556 |
| DP066-3-E-M1 | 27,679 | 358 | 0.9973 | 3.85 | 0.0526 | 426 |
| DP066-3-E-M2 | 27,679 | 357 | 0.9969 | 3.52 | 0.0889 | 438 |
| DP066-3-E-M3 | 27,679 | 357 | 0.998 | 4.44 | 0.0221 | 405 |
| DP066-10-C-F1 | 27,679 | 460 | 0.9974 | 4.62 | 0.0209 | 524 |
| DP066-10-C-F2 | 27,679 | 425 | 0.9976 | 4.39 | 0.0238 | 468 |
| DP066-10-C-F3 | 27,679 | 447 | 0.9977 | 4.57 | 0.0199 | 490 |
| DP066-10-C-M1 | 27,679 | 476 | 0.9966 | 4.63 | 0.0185 | 585 |
| DP066-10-C-M2 | 27,679 | 458 | 0.9969 | 4.41 | 0.0288 | 525 |
| DP066-10-C-M3 | 27,679 | 522 | 0.9971 | 4.65 | 0.0286 | 589 |
| DP066-10-E-F1 | 27,679 | 407 | 0.9968 | 3.64 | 0.0752 | 503 |
| DP066-10-E-F2 | 27,679 | 345 | 0.9973 | 3.43 | 0.0758 | 422 |
| DP066-10-E-F3 | 27,679 | 395 | 0.9971 | 3.51 | 0.0816 | 457 |
| DP066-10-E-M1 | 27,679 | 449 | 0.9975 | 4.62 | 0.0197 | 499 |
| DP066-10-E-M2 | 27,679 | 434 | 0.9977 | 4.62 | 0.0201 | 481 |
| DP066-10-E-M3 | 27,679 | 443 | 0.997 | 3.84 | 0.0706 | 513 |
| DP067-3-C-F1 | 27,679 | 364 | 0.997 | 3.41 | 0.0821 | 430 |
| DP067-3-C-F2 | 27,679 | 390 | 0.9975 | 3.84 | 0.0598 | 457 |
| DP067-3-C-F3 | 27,679 | 429 | 0.997 | 3.81 | 0.0636 | 518 |
| DP067-3-C-M1 | 27,679 | 381 | 0.9961 | 3.48 | 0.067 | 518 |
| DP067-3-E-F1 | 27,679 | 438 | 0.9976 | 4.56 | 0.0218 | 491 |
| DP067-3-E-F2 | 27,679 | 439 | 0.9975 | 4.78 | 0.0164 | 510 |
| DP067-3-E-F3 | 27,679 | 419 | 0.9976 | 4.04 | 0.0628 | 468 |
| DP067-3-E-M1 | 27,679 | 394 | 0.9971 | 3.81 | 0.0626 | 469 |
| DP067-3-E-M2 | 27,679 | 405 | 0.9973 | 3.67 | 0.0803 | 468 |
| DP067-3-E-M3 | 27,679 | 393 | 0.9969 | 2.7 | 0.3153 | 456 |
| DP067-10-C-F1 | 27,679 | 374 | 0.9969 | 3.63 | 0.0582 | 463 |
| DP067-10-C-F2 | 27,679 | 400 | 0.9977 | 4.13 | 0.041 | 467 |
| DP067-10-C-F3 | 27,679 | 443 | 0.998 | 4.73 | 0.0177 | 498 |
| DP067-10-C-M1 | 27,679 | 400 | 0.997 | 3.73 | 0.0598 | 462 |
| DP067-10-E-F1 | 27,679 | 398 | 0.9968 | 3.89 | 0.0418 | 501 |
| DP067-10-E-F2 | 27,679 | 424 | 0.9969 | 3.92 | 0.0487 | 502 |
| DP067-10-E-F3 | 27,679 | 417 | 0.9969 | 3.65 | 0.0766 | 482 |
| DP067-10-E-M1 | 27,679 | 462 | 0.9974 | 4.39 | 0.0369 | 508 |
| DP067-10-E-M2 | 27,679 | 441 | 0.9973 | 4.03 | 0.053 | 491 |
| DP067-10-E-M3 | 27,679 | 500 | 0.9967 | 4.74 | 0.0181 | 600 |
| DP068-3-C-F1 | 27,679 | 440 | 0.9969 | 3.51 | 0.0855 | 501 |
| DP068-3-C-F2 | 27,679 | 343 | 0.9965 | 3.13 | 0.0944 | 449 |
| DP068-3-C-F3 | 27,679 | 346 | 0.9967 | 3.36 | 0.071 | 426 |
| DP068-3-C-M1 | 27,679 | 516 | 0.9966 | 3.59 | 0.1441 | 583 |
| DP068-3-C-M2 | 27,679 | 465 | 0.9962 | 3.18 | 0.1819 | 559 |
| DP068-3-C-M3 | 27,679 | 416 | 0.9965 | 3.25 | 0.1725 | 485 |
| DP068-3-E-F1 | 27,679 | 371 | 0.9961 | 3.69 | 0.0637 | 541 |
| DP068-3-E-F2 | 27,679 | 366 | 0.9961 | 3.44 | 0.0851 | 469 |
| DP068-3-E-F3 | 27,679 | 364 | 0.9966 | 3.64 | 0.0665 | 488 |
| DP068-3-E-M1 | 27,679 | 447 | 0.9961 | 3.62 | 0.0794 | 533 |
| DP068-3-E-M2 | 27,679 | 480 | 0.9963 | 3.87 | 0.0727 | 585 |
| DP068-3-E-M3 | 27,679 | 416 | 0.9957 | 3.31 | 0.104 | 551 |
| DP068-10-C-F1 | 27,679 | 376 | 0.9963 | 3.16 | 0.1011 | 470 |
| DP068-10-C-F2 | 27,679 | 435 | 0.9958 | 3.49 | 0.069 | 529 |
| DP068-10-C-F3 | 27,679 | 344 | 0.997 | 3.42 | 0.07 | 427 |
| DP068-10-C-M1 | 27,679 | 499 | 0.9959 | 3.44 | 0.1356 | 597 |
| DP068-10-C-M2 | 27,679 | 505 | 0.9961 | 3.5 | 0.1536 | 610 |
| DP068-10-C-M3 | 27,679 | 307 | 0.9963 | 3.05 | 0.1124 | 454 |
| DP068-10-E-F1 | 27,679 | 484 | 0.9966 | 4.29 | 0.0468 | 561 |
| DP068-10-E-F2 | 27,679 | 391 | 0.997 | 3.83 | 0.0621 | 465 |
| DP068-10-E-F3 | 27,679 | 427 | 0.996 | 3.78 | 0.0584 | 538 |
| DP068-10-E-M1 | 27,679 | 448 | 0.9962 | 3.82 | 0.0639 | 527 |
| DP068-10-E-M2 | 27,679 | 454 | 0.9953 | 3.86 | 0.0534 | 609 |
| DP069-3-C-F1 | 27,679 | 337 | 0.9964 | 3.71 | 0.0465 | 450 |
| DP069-3-C-F2 | 27,679 | 326 | 0.9965 | 3.6 | 0.0564 | 454 |
| DP069-3-C-F3 | 27,679 | 387 | 0.9969 | 3.53 | 0.078 | 463 |
| DP069-3-C-M1 | 27,679 | 441 | 0.9972 | 4.32 | 0.0337 | 500 |
| DP069-3-C-M2 | 27,679 | 338 | 0.9973 | 3.62 | 0.069 | 452 |
| DP069-3-C-M3 | 27,679 | 439 | 0.9968 | 4.21 | 0.0493 | 517 |
| DP069-3-E-F1 | 27,679 | 476 | 0.9973 | 4.48 | 0.0289 | 533 |
| DP069-3-E-F2 | 27,679 | 347 | 0.9968 | 2.89 | 0.1422 | 425 |
| DP069-3-E-F3 | 27,679 | 308 | 0.9977 | 3.2 | 0.1026 | 366 |
| DP069-3-E-M1 | 27,679 | 434 | 0.9964 | 3.83 | 0.0516 | 524 |
| DP069-3-E-M2 | 27,679 | 467 | 0.9965 | 3.9 | 0.0605 | 570 |
| DP069-3-E-M3 | 27,679 | 476 | 0.9968 | 4.12 | 0.0386 | 569 |
| DP069-10-C-F1 | 27,679 | 374 | 0.9967 | 3.94 | 0.0372 | 458 |
| DP069-10-C-F2 | 27,679 | 459 | 0.9976 | 4.54 | 0.0249 | 528 |
| DP069-10-C-F3 | 27,679 | 466 | 0.997 | 4.29 | 0.0357 | 547 |
| DP069-10-C-M1 | 27,679 | 449 | 0.9975 | 4.43 | 0.0322 | 497 |
| DP069-10-C-M2 | 27,679 | 344 | 0.9979 | 3.74 | 0.0646 | 399 |
| DP069-10-C-M3 | 27,679 | 414 | 0.9968 | 3.95 | 0.0564 | 495 |
| DP069-10-E-F1 | 27,679 | 276 | 0.9976 | 3 | 0.1192 | 330 |
| DP069-10-E-F2 | 27,679 | 409 | 0.9971 | 3.35 | 0.144 | 488 |
| DP069-10-E-F3 | 27,679 | 459 | 0.997 | 4.28 | 0.0399 | 553 |
| DP069-10-E-M1 | 27,679 | 477 | 0.9967 | 4.33 | 0.0327 | 577 |
| DP069-10-E-M2 | 27,679 | 453 | 0.9972 | 4.07 | 0.044 | 508 |
| DP069-10-E-M3 | 27,679 | 481 | 0.9966 | 4.09 | 0.0436 | 554 |
| DP070-3-C-F1 | 27,679 | 402 | 0.9966 | 3.34 | 0.1245 | 488 |
| DP070-3-C-F2 | 27,679 | 476 | 0.9965 | 3.74 | 0.087 | 558 |
| DP070-3-C-F3 | 27,679 | 437 | 0.9967 | 3.7 | 0.0628 | 506 |
| DP070-3-C-M1 | 27,679 | 448 | 0.9968 | 2.89 | 0.2392 | 505 |
| DP070-3-C-M2 | 27,679 | 452 | 0.9961 | 3.44 | 0.1416 | 563 |
| DP070-3-C-M3 | 27,679 | 435 | 0.9959 | 3.03 | 0.1939 | 561 |
| DP070-3-E-F1 | 27,679 | 405 | 0.998 | 4.63 | 0.0183 | 441 |
| DP070-3-E-F2 | 27,679 | 192 | 0.998 | 3.06 | 0.0784 | 247 |
| DP070-3-E-F3 | 27,679 | 315 | 0.9983 | 4.39 | 0.0238 | 353 |
| DP070-3-E-M1 | 27,679 | 338 | 0.9972 | 3.49 | 0.061 | 413 |
| DP070-3-E-M2 | 27,679 | 316 | 0.998 | 4.16 | 0.0297 | 373 |
| DP070-3-E-M3 | 27,679 | 375 | 0.9965 | 3.54 | 0.0679 | 466 |
| DP070-10-C-F1 | 27,679 | 421 | 0.9962 | 3.38 | 0.1316 | 537 |
| DP070-10-C-F2 | 27,679 | 458 | 0.9964 | 3.39 | 0.1169 | 553 |
| DP070-10-C-F3 | 27,679 | 380 | 0.996 | 3.28 | 0.077 | 480 |
| DP070-10-C-M1 | 27,679 | 457 | 0.9967 | 3.6 | 0.1202 | 524 |
| DP070-10-C-M2 | 27,679 | 462 | 0.9964 | 3.31 | 0.1509 | 536 |
| DP070-10-C-M3 | 27,679 | 387 | 0.9963 | 2.74 | 0.1321 | 473 |
| DP070-10-E-F1 | 27,679 | 436 | 0.9965 | 4.15 | 0.037 | 544 |
| DP070-10-E-F2 | 27,679 | 356 | 0.9975 | 4.06 | 0.0373 | 427 |
| DP070-10-E-F3 | 27,679 | 410 | 0.997 | 4.36 | 0.0258 | 505 |
| DP070-10-E-M1 | 27,679 | 345 | 0.9983 | 4.07 | 0.0371 | 373 |
| DP070-10-E-M2 | 27,679 | 497 | 0.997 | 4.57 | 0.0239 | 565 |
| DP070-10-E-M3 | 27,679 | 414 | 0.9967 | 4.1 | 0.0413 | 499 |
| DP071-3-C-F1 | 27,679 | 456 | 0.9964 | 3.9 | 0.0465 | 539 |
| DP071-3-C-F2 | 27,679 | 449 | 0.9964 | 3.85 | 0.0535 | 548 |
| DP071-3-C-F3 | 27,679 | 438 | 0.9966 | 3.47 | 0.0993 | 503 |
| DP071-3-C-M1 | 27,679 | 488 | 0.9965 | 3.99 | 0.0594 | 572 |
| DP071-3-C-M2 | 27,679 | 403 | 0.9962 | 3.15 | 0.1703 | 501 |
| DP071-3-C-M3 | 27,679 | 513 | 0.996 | 3.54 | 0.1278 | 654 |
| DP071-3-E-F1 | 27,679 | 422 | 0.9969 | 4.04 | 0.0434 | 507 |
| DP071-3-E-F2 | 27,679 | 498 | 0.9966 | 4.57 | 0.0229 | 595 |
| DP071-3-E-F3 | 27,679 | 468 | 0.997 | 4.23 | 0.0352 | 527 |
| DP071-3-E-M1 | 27,679 | 528 | 0.9966 | 4.68 | 0.0224 | 616 |
| DP071-3-E-M2 | 27,679 | 421 | 0.9977 | 4.52 | 0.024 | 466 |
| DP071-3-E-M3 | 27,679 | 466 | 0.9971 | 4.42 | 0.0274 | 530 |
| DP071-10-C-F1 | 27,679 | 384 | 0.9966 | 3.44 | 0.0647 | 466 |
| DP071-10-C-F2 | 27,679 | 397 | 0.9966 | 3.49 | 0.0673 | 474 |
| DP071-10-C-F3 | 27,679 | 305 | 0.9971 | 2.88 | 0.1548 | 370 |
| DP071-10-C-M1 | 27,679 | 419 | 0.9963 | 3.4 | 0.1175 | 518 |
| DP071-10-C-M2 | 27,679 | 469 | 0.9956 | 3.33 | 0.1608 | 617 |
| DP071-10-C-M3 | 27,679 | 502 | 0.9964 | 3.68 | 0.1095 | 582 |
| DP071-10-E-F1 | 27,679 | 354 | 0.997 | 2.88 | 0.2114 | 428 |
| DP071-10-E-F2 | 27,679 | 506 | 0.9966 | 4.35 | 0.0338 | 583 |
| DP071-10-E-F3 | 27,679 | 496 | 0.996 | 3.91 | 0.0491 | 595 |
| DP071-10-E-M1 | 27,679 | 408 | 0.9963 | 3.56 | 0.0812 | 533 |
| DP071-10-E-M2 | 27,679 | 290 | 0.9972 | 3.01 | 0.0989 | 352 |
| DP071-10-E-M3 | 27,679 | 485 | 0.9963 | 4.07 | 0.0431 | 571 |
| DP072-3-C-F1 | 27,679 | 488 | 0.9963 | 3.86 | 0.0643 | 582 |
| DP072-3-C-F2 | 27,679 | 395 | 0.9965 | 3.26 | 0.1105 | 480 |
| DP072-3-C-F3 | 27,679 | 237 | 0.9974 | 2.26 | 0.2376 | 304 |
| DP072-3-C-M1 | 27,679 | 496 | 0.9963 | 3.26 | 0.1848 | 571 |
| DP072-3-C-M2 | 27,679 | 483 | 0.9963 | 3.47 | 0.1357 | 608 |
| DP072-3-C-M3 | 27,679 | 503 | 0.9973 | 4.03 | 0.0774 | 554 |
| DP072-3-E-F1 | 27,679 | 394 | 0.9971 | 4.05 | 0.0476 | 497 |
| DP072-3-E-F2 | 27,679 | 426 | 0.9974 | 4.15 | 0.0588 | 497 |
| DP072-3-E-F3 | 27,679 | 437 | 0.9973 | 4.23 | 0.0352 | 494 |
| DP072-3-E-M1 | 27,679 | 453 | 0.9965 | 3.86 | 0.056 | 552 |
| DP072-3-E-M2 | 27,679 | 446 | 0.9969 | 4.27 | 0.0389 | 517 |
| DP072-3-E-M3 | 27,679 | 419 | 0.9972 | 4.17 | 0.0334 | 502 |
| DP072-10-C-F1 | 27,679 | 473 | 0.997 | 3.7 | 0.0847 | 536 |
| DP072-10-C-F2 | 27,679 | 398 | 0.9969 | 3.21 | 0.123 | 461 |
| DP072-10-C-F3 | 27,679 | 336 | 0.9962 | 2.54 | 0.2091 | 454 |
| DP072-10-C-M1 | 27,679 | 504 | 0.9964 | 3.45 | 0.1669 | 603 |
| DP072-10-C-M2 | 27,679 | 467 | 0.996 | 3.3 | 0.1546 | 597 |
| DP072-10-C-M3 | 27,679 | 471 | 0.9962 | 3.62 | 0.0962 | 576 |
| DP072-10-E-F1 | 27,679 | 415 | 0.9964 | 3.84 | 0.0615 | 516 |
| DP072-10-E-F2 | 27,679 | 428 | 0.9962 | 3.77 | 0.0607 | 547 |
| DP072-10-E-F3 | 27,679 | 934 | 0.9886 | 4.75 | 0.0219 | 1261 |
| DP072-10-E-M1 | 27,679 | 401 | 0.996 | 3.55 | 0.0886 | 521 |
| DP072-10-E-M2 | 27,679 | 477 | 0.9961 | 3.7 | 0.0871 | 600 |
| DP072-10-E-M3 | 27,679 | 447 | 0.9971 | 3.84 | 0.0733 | 496 |
| DP073-3-C-F1 | 27,679 | 530 | 0.9974 | 4.87 | 0.0144 | 591 |
| DP073-3-C-F2 | 27,679 | 511 | 0.9962 | 4.28 | 0.0328 | 625 |
| DP073-3-C-F3 | 27,679 | 529 | 0.9973 | 4.84 | 0.016 | 588 |
| DP073-3-C-M1 | 27,679 | 377 | 0.9965 | 2.73 | 0.2436 | 467 |
| DP073-3-C-M2 | 27,679 | 497 | 0.9966 | 3.73 | 0.1149 | 581 |
| DP073-3-C-M3 | 27,679 | 347 | 0.9964 | 2.87 | 0.1481 | 462 |
| DP073-3-E-F1 | 27,679 | 429 | 0.997 | 4.16 | 0.034 | 496 |
| DP073-3-E-F2 | 27,679 | 399 | 0.9964 | 3.5 | 0.0831 | 487 |
| DP073-3-E-F3 | 27,679 | 407 | 0.9971 | 4.5 | 0.0219 | 516 |
| DP073-3-E-M1 | 27,679 | 399 | 0.9969 | 3.76 | 0.0578 | 475 |
| DP073-3-E-M2 | 27,679 | 370 | 0.9974 | 3.37 | 0.1365 | 435 |
| DP073-3-E-M3 | 27,679 | 381 | 0.9974 | 3.59 | 0.0704 | 438 |
| DP073-10-C-F1 | 27,679 | 528 | 0.9973 | 4.71 | 0.0193 | 596 |
| DP073-10-C-F2 | 27,679 | 489 | 0.9963 | 4.15 | 0.0346 | 580 |
| DP073-10-C-F3 | 27,679 | 534 | 0.9973 | 4.77 | 0.0179 | 593 |
| DP073-10-C-M1 | 27,679 | 374 | 0.9962 | 2.61 | 0.2625 | 475 |
| DP073-10-C-M2 | 27,679 | 499 | 0.9965 | 3.67 | 0.1283 | 578 |
| DP073-10-C-M3 | 27,679 | 402 | 0.996 | 2.9 | 0.1523 | 497 |
| DP073-10-E-F1 | 27,679 | 379 | 0.9972 | 3.7 | 0.059 | 443 |
| DP073-10-E-F2 | 27,679 | 382 | 0.9963 | 3.79 | 0.0513 | 494 |
| DP073-10-E-F3 | 27,679 | 412 | 0.9976 | 3.99 | 0.0445 | 460 |
| DP073-10-E-M1 | 27,679 | 395 | 0.9976 | 3.97 | 0.0501 | 449 |
| DP073-10-E-M2 | 27,679 | 296 | 0.9974 | 3.06 | 0.095 | 357 |
| DP073-10-E-M3 | 27,679 | 396 | 0.9969 | 4.19 | 0.0335 | 501 |
| DP074-3-C-F1 | 27,679 | 201 | 0.9985 | 2.42 | 0.1866 | 224 |
| DP074-3-C-F2 | 27,679 | 290 | 0.9967 | 2.32 | 0.2667 | 407 |
| DP074-3-C-F3 | 27,679 | 293 | 0.9969 | 2.6 | 0.1952 | 387 |
| DP074-3-C-M1 | 27,679 | 278 | 0.997 | 2.53 | 0.2027 | 347 |
| DP074-3-C-M2 | 27,679 | 407 | 0.9959 | 2.99 | 0.1764 | 522 |
| DP074-3-C-M3 | 27,679 | 497 | 0.997 | 3.83 | 0.1063 | 564 |
| DP074-3-E-F1 | 27,679 | 414 | 0.9976 | 4.08 | 0.0428 | 461 |
| DP074-3-E-F2 | 27,679 | 409 | 0.9975 | 3.95 | 0.061 | 459 |
| DP074-3-E-F3 | 27,679 | 405 | 0.9973 | 4.18 | 0.04 | 464 |
| DP074-3-E-M1 | 27,679 | 241 | 0.9978 | 3.22 | 0.0867 | 300 |
| DP074-3-E-M2 | 27,679 | 253 | 0.998 | 3.21 | 0.0776 | 296 |
| DP074-3-E-M3 | 27,679 | 260 | 0.9974 | 3.25 | 0.0733 | 354 |
| DP074-10-C-F1 | 27,679 | 212 | 0.9977 | 2.09 | 0.2424 | 263 |
| DP074-10-C-F2 | 27,679 | 278 | 0.997 | 2.01 | 0.3224 | 353 |
| DP074-10-C-F3 | 27,679 | 229 | 0.9978 | 2.49 | 0.1918 | 286 |
| DP074-10-C-M1 | 27,679 | 513 | 0.9965 | 3.74 | 0.1051 | 590 |
| DP074-10-C-M2 | 27,679 | 451 | 0.9965 | 3.44 | 0.1324 | 548 |
| DP074-10-C-M3 | 27,679 | 401 | 0.9962 | 2.57 | 0.3057 | 493 |
| DP074-10-E-F1 | 27,679 | 443 | 0.997 | 4.06 | 0.0567 | 519 |
| DP074-10-E-F2 | 27,679 | 380 | 0.9978 | 4.23 | 0.0299 | 427 |
| DP074-10-E-F3 | 27,679 | 386 | 0.9975 | 3.81 | 0.0582 | 428 |
| DP074-10-E-M1 | 27,679 | 349 | 0.9973 | 3.46 | 0.1 | 418 |
| DP074-10-E-M2 | 27,679 | 407 | 0.997 | 3.78 | 0.0733 | 470 |
| DP074-10-E-M3 | 27,679 | 420 | 0.9969 | 4.1 | 0.0414 | 505 |
| DP075-3-C-F1 | 27,679 | 232 | 0.9975 | 2.93 | 0.1154 | 315 |
| DP075-3-C-F2 | 27,679 | 462 | 0.9956 | 2.96 | 0.219 | 592 |
| DP075-3-C-F3 | 27,679 | 295 | 0.9965 | 2.08 | 0.3352 | 401 |
| DP075-3-C-M1 | 27,679 | 390 | 0.9963 | 3.01 | 0.17 | 481 |
| DP075-3-C-M2 | 27,679 | 495 | 0.9964 | 3.47 | 0.1391 | 568 |
| DP075-3-C-M3 | 27,679 | 478 | 0.9954 | 3.43 | 0.1139 | 619 |
| DP075-3-E-F1 | 27,679 | 446 | 0.9963 | 4.07 | 0.0478 | 525 |
| DP075-3-E-F2 | 27,679 | 355 | 0.9971 | 3.5 | 0.0846 | 432 |
| DP075-3-E-F3 | 27,679 | 430 | 0.996 | 3.76 | 0.06 | 543 |
| DP075-3-E-M1 | 27,679 | 337 | 0.996 | 3.08 | 0.1037 | 478 |
| DP075-3-E-M2 | 27,679 | 309 | 0.9974 | 3.1 | 0.1044 | 378 |
| DP075-3-E-M3 | 27,679 | 337 | 0.9975 | 3.94 | 0.0441 | 406 |
| DP075-10-C-F1 | 27,679 | 265 | 0.997 | 2.82 | 0.1378 | 357 |
| DP075-10-C-F2 | 27,679 | 331 | 0.9962 | 2.56 | 0.263 | 464 |
| DP075-10-C-F3 | 27,679 | 308 | 0.9962 | 2.17 | 0.3015 | 432 |
| DP075-10-C-M1 | 27,679 | 398 | 0.996 | 2.79 | 0.2037 | 520 |
| DP075-10-C-M2 | 27,679 | 461 | 0.9959 | 3.1 | 0.1911 | 587 |
| DP075-10-C-M3 | 27,679 | 443 | 0.996 | 3.17 | 0.1635 | 554 |
| DP075-10-E-F1 | 27,679 | 417 | 0.9969 | 4.18 | 0.0377 | 498 |
| DP075-10-E-F2 | 27,679 | 414 | 0.9972 | 3.91 | 0.0579 | 486 |
| DP075-10-E-F3 | 27,679 | 353 | 0.9961 | 3.11 | 0.1068 | 462 |
| DP075-10-E-M1 | 27,679 | 391 | 0.9969 | 3.68 | 0.079 | 482 |
| DP075-10-E-M2 | 27,679 | 382 | 0.9974 | 3.87 | 0.0494 | 443 |
| DP075-10-E-M3 | 27,679 | 416 | 0.9973 | 4 | 0.0439 | 474 |
| DP076-3-C-F1 | 27,679 | 341 | 0.997 | 3.47 | 0.0682 | 409 |
| DP076-3-C-F2 | 27,679 | 399 | 0.998 | 4.65 | 0.0221 | 439 |
| DP076-3-C-F3 | 27,679 | 399 | 0.9977 | 3.88 | 0.0511 | 452 |
| DP076-3-C-M1 | 27,679 | 317 | 0.9974 | 3.27 | 0.0947 | 386 |
| DP076-3-C-M2 | 27,679 | 392 | 0.9974 | 3.5 | 0.0765 | 435 |
| DP076-3-C-M3 | 27,679 | 395 | 0.9969 | 3.94 | 0.0459 | 491 |
| DP076-3-E-F1 | 27,679 | 407 | 0.9978 | 4.22 | 0.0397 | 451 |
| DP076-3-E-F2 | 27,679 | 457 | 0.9975 | 4.7 | 0.0204 | 503 |
| DP076-3-E-F3 | 27,679 | 437 | 0.9974 | 4.42 | 0.0304 | 504 |
| DP076-3-E-M1 | 27,679 | 329 | 0.9972 | 3.1 | 0.1628 | 394 |
| DP076-3-E-M2 | 27,679 | 392 | 0.9972 | 3.46 | 0.1092 | 454 |
| DP076-3-E-M3 | 27,679 | 295 | 0.9978 | 3.01 | 0.1295 | 342 |
| DP076-10-C-F1 | 27,679 | 373 | 0.9973 | 4 | 0.043 | 437 |
| DP076-10-C-F2 | 27,679 | 407 | 0.9975 | 3.87 | 0.0577 | 508 |
| DP076-10-C-F3 | 27,679 | 332 | 0.997 | 3.49 | 0.0689 | 405 |
| DP076-10-C-M1 | 27,679 | 423 | 0.9973 | 4.35 | 0.0269 | 488 |
| DP076-10-C-M2 | 27,679 | 424 | 0.9965 | 3.67 | 0.0746 | 512 |
| DP076-10-C-M3 | 27,679 | 450 | 0.9973 | 4.37 | 0.0367 | 509 |
| DP076-10-E-F1 | 27,679 | 378 | 0.9971 | 3.55 | 0.0811 | 441 |
| DP076-10-E-F2 | 27,679 | 435 | 0.9978 | 4.4 | 0.0287 | 487 |
| DP076-10-E-F3 | 27,679 | 401 | 0.9979 | 4.29 | 0.0317 | 447 |
| DP076-10-E-M1 | 27,679 | 437 | 0.9978 | 4.3 | 0.0333 | 484 |
| DP076-10-E-M2 | 27,679 | 376 | 0.9974 | 3.94 | 0.0438 | 451 |
| DP076-10-E-M3 | 27,679 | 438 | 0.9975 | 4.3 | 0.0391 | 490 |
| DP077-3-C-F1 | 27,679 | 394 | 0.996 | 3.06 | 0.1469 | 546 |
| DP077-3-C-F2 | 27,679 | 436 | 0.9963 | 3.76 | 0.0667 | 550 |
| DP077-3-C-F3 | 27,679 | 489 | 0.9964 | 3.59 | 0.1219 | 588 |
| DP077-3-C-M1 | 27,679 | 488 | 0.9966 | 4.02 | 0.0623 | 552 |
| DP077-3-C-M2 | 27,679 | 485 | 0.9963 | 3.97 | 0.0544 | 581 |
| DP077-3-C-M3 | 27,679 | 500 | 0.9966 | 3.8 | 0.092 | 565 |
| DP077-3-E-F1 | 27,679 | 496 | 0.996 | 4.81 | 0.0151 | 663 |
| DP077-3-E-F2 | 27,679 | 286 | 0.9972 | 3.4 | 0.0733 | 367 |
| DP077-3-E-F3 | 27,679 | 516 | 0.9965 | 4.67 | 0.0195 | 601 |
| DP077-3-E-M1 | 27,679 | 476 | 0.9968 | 4.76 | 0.0169 | 565 |
| DP077-3-E-M2 | 27,679 | 439 | 0.9971 | 4.52 | 0.0221 | 524 |
| DP077-3-E-M3 | 27,679 | 470 | 0.9958 | 4.28 | 0.0407 | 612 |
| DP077-10-C-F1 | 27,679 | 396 | 0.9959 | 2.95 | 0.1552 | 491 |
| DP077-10-C-F2 | 27,679 | 496 | 0.9962 | 3.92 | 0.0648 | 579 |
| DP077-10-C-F3 | 27,679 | 504 | 0.9963 | 3.58 | 0.1362 | 596 |
| DP077-10-C-M1 | 27,679 | 364 | 0.9961 | 3.2 | 0.1288 | 487 |
| DP077-10-C-M2 | 27,679 | 466 | 0.9957 | 3.55 | 0.1058 | 587 |
| DP077-10-C-M3 | 27,679 | 488 | 0.9958 | 3.4 | 0.1499 | 591 |
| DP077-10-E-F1 | 27,679 | 411 | 0.9969 | 3.67 | 0.0927 | 475 |
| DP077-10-E-F2 | 27,679 | 406 | 0.9974 | 4.46 | 0.0267 | 492 |
| DP077-10-E-F3 | 27,679 | 349 | 0.9965 | 3.41 | 0.0772 | 463 |
| DP077-10-E-M1 | 27,679 | 450 | 0.997 | 4.57 | 0.0259 | 542 |
| DP077-10-E-M2 | 27,679 | 286 | 0.9977 | 3.48 | 0.0758 | 361 |
| DP077-10-E-M3 | 27,679 | 499 | 0.9962 | 4.31 | 0.047 | 595 |
| DP078-3-C-F1 | 27,679 | 291 | 0.9975 | 3.06 | 0.111 | 408 |
| DP078-3-C-F2 | 27,679 | 352 | 0.997 | 3.1 | 0.1063 | 444 |
| DP078-3-C-F3 | 27,679 | 336 | 0.9974 | 3.28 | 0.0931 | 407 |
| DP078-3-C-M1 | 27,679 | 317 | 0.9977 | 3.77 | 0.0581 | 415 |
| DP078-3-C-M2 | 27,679 | 353 | 0.9972 | 4 | 0.0515 | 439 |
| DP078-3-C-M3 | 27,679 | 360 | 0.9971 | 3.77 | 0.0692 | 459 |
| DP078-3-E-F1 | 27,679 | 323 | 0.9971 | 3.6 | 0.0607 | 433 |
| DP078-3-E-F2 | 27,679 | 309 | 0.9973 | 3.09 | 0.1122 | 382 |
| DP078-3-E-F3 | 27,679 | 301 | 0.997 | 2.92 | 0.119 | 407 |
| DP078-3-E-M1 | 27,679 | 341 | 0.9971 | 3.45 | 0.0731 | 416 |
| DP078-3-E-M2 | 27,679 | 327 | 0.9974 | 3.53 | 0.0663 | 407 |
| DP078-3-E-M3 | 27,679 | 360 | 0.9972 | 3.84 | 0.0463 | 435 |
| DP078-10-C-F1 | 27,679 | 289 | 0.9974 | 2.84 | 0.1395 | 342 |
| DP078-10-C-F2 | 27,679 | 301 | 0.9966 | 2.7 | 0.1629 | 390 |
| DP078-10-C-M1 | 27,679 | 298 | 0.9976 | 2.69 | 0.2303 | 369 |
| DP078-10-C-M2 | 27,679 | 374 | 0.9974 | 3.56 | 0.1274 | 451 |
| DP078-10-C-M3 | 27,679 | 343 | 0.9975 | 3.33 | 0.1655 | 419 |
| DP078-10-E-F1 | 27,679 | 318 | 0.9979 | 3.76 | 0.05 | 356 |
| DP078-10-E-F2 | 27,679 | 369 | 0.998 | 4.28 | 0.041 | 410 |
| DP078-10-E-F3 | 27,679 | 287 | 0.9979 | 3.1 | 0.1241 | 331 |
| DP078-10-E-M1 | 27,679 | 406 | 0.9964 | 4.23 | 0.0298 | 568 |
| DP078-10-E-M2 | 27,679 | 325 | 0.9975 | 3.42 | 0.0916 | 392 |
| DP081-3-C-F1 | 27,679 | 518 | 0.9967 | 4.96 | 0.0147 | 643 |
| DP081-3-C-F2 | 27,679 | 517 | 0.9961 | 4.39 | 0.0322 | 618 |
| DP081-3-C-F3 | 27,679 | 560 | 0.9965 | 4.71 | 0.0243 | 653 |
| DP081-3-C-M1 | 27,679 | 446 | 0.9961 | 3.81 | 0.0567 | 538 |
| DP081-3-C-M2 | 27,679 | 482 | 0.9966 | 3.94 | 0.0513 | 552 |
| DP081-3-C-M3 | 27,679 | 512 | 0.9958 | 4.07 | 0.0435 | 623 |
| DP081-3-E-F1 | 27,679 | 465 | 0.9971 | 4.54 | 0.0264 | 522 |
| DP081-3-E-F2 | 27,679 | 516 | 0.9958 | 4.3 | 0.042 | 633 |
| DP081-3-E-F3 | 27,679 | 438 | 0.996 | 3.25 | 0.1158 | 565 |
| DP081-3-E-M1 | 27,679 | 424 | 0.995 | 3.17 | 0.085 | 574 |
| DP081-3-E-M2 | 27,679 | 371 | 0.9961 | 2.9 | 0.1352 | 453 |
| DP081-3-E-M3 | 27,679 | 438 | 0.9959 | 3.5 | 0.0674 | 531 |
| DP081-10-C-F1 | 27,679 | 343 | 0.9962 | 3.42 | 0.0768 | 519 |
| DP081-10-C-F2 | 27,679 | 448 | 0.9969 | 4.09 | 0.0423 | 514 |
| DP081-10-C-F3 | 27,679 | 521 | 0.9963 | 4.52 | 0.0245 | 626 |
| DP081-10-C-M1 | 27,679 | 360 | 0.9963 | 3.48 | 0.0706 | 467 |
| DP081-10-C-M2 | 27,679 | 424 | 0.9953 | 3.69 | 0.0519 | 571 |
| DP081-10-C-M3 | 27,679 | 467 | 0.9962 | 3.95 | 0.049 | 560 |
| DP081-10-E-F1 | 27,679 | 347 | 0.9965 | 3.44 | 0.0761 | 448 |
| DP081-10-E-F2 | 27,679 | 404 | 0.9965 | 3.72 | 0.0579 | 497 |
| DP081-10-E-F3 | 27,679 | 460 | 0.9962 | 4.27 | 0.0357 | 565 |
| DP081-10-E-M1 | 27,679 | 547 | 0.9965 | 4.45 | 0.0268 | 619 |
| DP081-10-E-M2 | 27,679 | 436 | 0.9949 | 3.17 | 0.0999 | 607 |
| DP081-10-E-M3 | 27,679 | 496 | 0.9955 | 3.9 | 0.0515 | 605 |
| DP082-3-C-F1 | 27,679 | 475 | 0.9974 | 4.62 | 0.0214 | 535 |
| DP082-3-C-F2 | 27,679 | 495 | 0.9968 | 4.69 | 0.0181 | 568 |
| DP082-3-C-F3 | 27,679 | 438 | 0.9966 | 4 | 0.044 | 516 |
| DP082-3-C-M1 | 27,679 | 752 | 0.9925 | 4.18 | 0.0444 | 933 |
| DP082-3-C-M2 | 27,679 | 429 | 0.9972 | 4.11 | 0.0377 | 493 |
| DP082-3-C-M3 | 27,679 | 425 | 0.9961 | 3.66 | 0.071 | 545 |
| DP082-3-E-F1 | 27,679 | 469 | 0.9964 | 4.37 | 0.0356 | 566 |
| DP082-3-E-F2 | 27,679 | 452 | 0.9963 | 4.1 | 0.0527 | 566 |
| DP082-3-E-F3 | 27,679 | 400 | 0.9974 | 4.42 | 0.0312 | 475 |
| DP082-3-E-M1 | 27,679 | 473 | 0.9965 | 4.24 | 0.0355 | 566 |
| DP082-3-E-M2 | 27,679 | 457 | 0.9968 | 4.43 | 0.0259 | 535 |
| DP082-3-E-M3 | 27,679 | 418 | 0.9966 | 4.02 | 0.0464 | 507 |
| DP082-10-C-F1 | 27,679 | 414 | 0.9969 | 4.06 | 0.0413 | 492 |
| DP082-10-C-F2 | 27,679 | 497 | 0.9961 | 4.63 | 0.02 | 587 |
| DP082-10-C-F3 | 27,679 | 425 | 0.9964 | 4.06 | 0.0394 | 520 |
| DP082-10-C-M1 | 27,679 | 450 | 0.997 | 4.06 | 0.0629 | 540 |
| DP082-10-C-M2 | 27,679 | 429 | 0.9976 | 4.4 | 0.0302 | 479 |
| DP082-10-C-M3 | 27,679 | 435 | 0.9961 | 3.96 | 0.0485 | 553 |
| DP082-10-E-F1 | 27,679 | 459 | 0.9967 | 4.45 | 0.0279 | 559 |
| DP082-10-E-F2 | 27,679 | 460 | 0.9971 | 4.51 | 0.0258 | 539 |
| DP082-10-E-F3 | 27,679 | 437 | 0.9976 | 4.54 | 0.0208 | 482 |
| DP082-10-E-M1 | 27,679 | 440 | 0.9965 | 3.94 | 0.0753 | 528 |
| DP082-10-E-M2 | 27,679 | 467 | 0.9964 | 4.05 | 0.0515 | 552 |
| DP082-10-E-M3 | 27,679 | 382 | 0.9973 | 3.47 | 0.117 | 440 |
| DP083-3-C-F1 | 27,679 | 343 | 0.998 | 3.83 | 0.0566 | 388 |
| DP083-3-C-F2 | 27,679 | 418 | 0.9973 | 4.17 | 0.0444 | 511 |
| DP083-3-C-F3 | 27,679 | 326 | 0.9975 | 3.7 | 0.0521 | 386 |
| DP083-3-C-M1 | 27,679 | 201 | 0.9979 | 2.68 | 0.1688 | 287 |
| DP083-3-C-M2 | 27,679 | 262 | 0.9971 | 3.14 | 0.0816 | 375 |
| DP083-3-C-M3 | 27,679 | 387 | 0.9974 | 3.32 | 0.1263 | 433 |
| DP083-3-E-F1 | 27,679 | 298 | 0.9977 | 3.07 | 0.1085 | 353 |
| DP083-3-E-F2 | 27,679 | 280 | 0.9969 | 2.81 | 0.1332 | 458 |
| DP083-3-E-F3 | 27,679 | 331 | 0.9971 | 3.23 | 0.0963 | 408 |
| DP083-3-E-M1 | 27,679 | 164 | 0.9986 | 2.75 | 0.1121 | 208 |
| DP083-3-E-M2 | 27,679 | 314 | 0.9973 | 3.56 | 0.0625 | 396 |
| DP083-3-E-M3 | 27,679 | 328 | 0.9971 | 3.63 | 0.062 | 416 |
| DP083-10-C-F1 | 27,679 | 342 | 0.9974 | 2.89 | 0.1686 | 403 |
| DP083-10-C-F2 | 27,679 | 302 | 0.998 | 2.41 | 0.2518 | 341 |
| DP083-10-C-F3 | 27,679 | 337 | 0.9973 | 3.29 | 0.1046 | 400 |
| DP083-10-C-M1 | 27,679 | 293 | 0.9972 | 3.01 | 0.1384 | 390 |
| DP083-10-C-M2 | 27,679 | 269 | 0.9975 | 3.02 | 0.1107 | 342 |
| DP083-10-C-M3 | 27,679 | 279 | 0.9976 | 3.31 | 0.1009 | 358 |
| DP083-10-E-F1 | 27,679 | 303 | 0.997 | 2.42 | 0.2208 | 380 |
| DP083-10-E-F2 | 27,679 | 378 | 0.9979 | 4.11 | 0.0555 | 421 |
| DP083-10-E-F3 | 27,679 | 346 | 0.9973 | 3.96 | 0.047 | 419 |
| DP083-10-E-M1 | 27,679 | 385 | 0.9966 | 3.54 | 0.0754 | 476 |
| DP083-10-E-M2 | 27,679 | 296 | 0.9966 | 2.83 | 0.1525 | 442 |
| DP083-10-E-M3 | 27,679 | 241 | 0.9978 | 3.03 | 0.0984 | 292 |
| DP085-3-C-F1 | 27,679 | 494 | 0.9978 | 4.57 | 0.0306 | 534 |
| DP085-3-C-F2 | 27,679 | 319 | 0.9971 | 3.5 | 0.056 | 388 |
| DP085-3-C-F3 | 27,679 | 306 | 0.997 | 3.15 | 0.1007 | 432 |
| DP085-3-C-M1 | 27,679 | 263 | 0.9977 | 3.08 | 0.104 | 338 |
| DP085-3-C-M2 | 27,679 | 263 | 0.9969 | 2.37 | 0.2766 | 370 |
| DP085-3-C-M3 | 27,679 | 375 | 0.9968 | 3.4 | 0.1134 | 453 |
| DP085-3-E-F1 | 27,679 | 315 | 0.9978 | 3.39 | 0.0964 | 370 |
| DP085-3-E-F2 | 27,679 | 367 | 0.9978 | 3.97 | 0.047 | 422 |
| DP085-3-E-F3 | 27,679 | 397 | 0.9978 | 4.34 | 0.0315 | 453 |
| DP085-3-E-M1 | 27,679 | 263 | 0.9973 | 3.14 | 0.081 | 353 |
| DP085-3-E-M2 | 27,679 | 381 | 0.9974 | 3.42 | 0.1542 | 446 |
| DP085-3-E-M3 | 27,679 | 293 | 0.9969 | 2.15 | 0.27 | 397 |
| DP085-10-C-F1 | 27,679 | 362 | 0.9979 | 3.76 | 0.0753 | 404 |
| DP085-10-C-F2 | 27,679 | 399 | 0.9971 | 3.75 | 0.0591 | 456 |
| DP085-10-C-F3 | 27,679 | 343 | 0.9973 | 3.76 | 0.0427 | 401 |
| DP085-10-C-M1 | 27,679 | 408 | 0.9974 | 4.28 | 0.0326 | 467 |
| DP085-10-C-M2 | 27,679 | 403 | 0.9978 | 4.34 | 0.0265 | 454 |
| DP085-10-C-M3 | 27,679 | 369 | 0.9979 | 3.91 | 0.0604 | 411 |
| DP085-10-E-F1 | 27,679 | 367 | 0.9973 | 2.93 | 0.1769 | 433 |
| DP085-10-E-F2 | 27,679 | 361 | 0.9967 | 3.73 | 0.0569 | 456 |
| DP085-10-E-F3 | 27,679 | 280 | 0.9973 | 3.03 | 0.1026 | 353 |
| DP085-10-E-M1 | 27,679 | 447 | 0.997 | 4.4 | 0.0291 | 536 |
| DP085-10-E-M2 | 27,679 | 417 | 0.9977 | 4.45 | 0.0226 | 459 |
| DP085-10-E-M3 | 27,679 | 422 | 0.9976 | 4.52 | 0.0214 | 480 |
| DP086-3-C-F1 | 27,679 | 505 | 0.9971 | 4.65 | 0.0197 | 562 |
| DP086-3-C-F2 | 27,679 | 481 | 0.9967 | 4.4 | 0.0285 | 547 |
| DP086-3-C-F3 | 27,679 | 508 | 0.9958 | 4.03 | 0.0735 | 639 |
| DP086-3-C-M1 | 27,679 | 444 | 0.9966 | 4.08 | 0.0375 | 543 |
| DP086-3-C-M2 | 27,679 | 484 | 0.9973 | 4.52 | 0.0354 | 546 |
| DP086-3-C-M3 | 27,679 | 405 | 0.9973 | 3.98 | 0.0396 | 459 |
| DP086-3-E-F1 | 27,679 | 417 | 0.997 | 4.01 | 0.0672 | 491 |
| DP086-3-E-F2 | 27,679 | 463 | 0.9972 | 4.42 | 0.0366 | 526 |
| DP086-3-E-F3 | 27,679 | 423 | 0.9975 | 4.2 | 0.034 | 486 |
| DP086-3-E-M1 | 27,679 | 432 | 0.9975 | 4.36 | 0.0328 | 489 |
| DP086-3-E-M2 | 27,679 | 435 | 0.9973 | 4.54 | 0.02 | 501 |
| DP086-3-E-M3 | 27,679 | 476 | 0.9974 | 4.61 | 0.0222 | 515 |
| DP086-10-C-F1 | 27,679 | 461 | 0.9973 | 4.63 | 0.0188 | 517 |
| DP086-10-C-F2 | 27,679 | 424 | 0.998 | 4.71 | 0.0162 | 466 |
| DP086-10-C-F3 | 27,679 | 421 | 0.9971 | 3.93 | 0.0608 | 489 |
| DP086-10-C-M1 | 27,679 | 399 | 0.996 | 3.9 | 0.0415 | 547 |
| DP086-10-C-M2 | 27,679 | 501 | 0.9977 | 4.62 | 0.0312 | 546 |
| DP086-10-C-M3 | 27,679 | 496 | 0.997 | 4.58 | 0.0229 | 575 |
| DP086-10-E-F1 | 27,679 | 501 | 0.996 | 3.89 | 0.0585 | 608 |
| DP086-10-E-F2 | 27,679 | 466 | 0.9974 | 4.24 | 0.0523 | 524 |
| DP086-10-E-F3 | 27,679 | 572 | 0.9981 | 4.72 | 0.0204 | 610 |
| DP086-10-E-M1 | 27,679 | 396 | 0.9977 | 3.82 | 0.0853 | 435 |
| DP086-10-E-M2 | 27,679 | 432 | 0.9965 | 3.86 | 0.0776 | 520 |
| DP086-10-E-M3 | 27,679 | 455 | 0.9964 | 4.1 | 0.0458 | 573 |
| DP087-3-C-F1 | 27,679 | 439 | 0.9974 | 4.71 | 0.0163 | 512 |
| DP087-3-C-F2 | 27,679 | 332 | 0.9983 | 4.19 | 0.0372 | 363 |
| DP087-3-C-F3 | 27,679 | 444 | 0.9974 | 4.5 | 0.0273 | 503 |
| DP087-3-C-M1 | 27,679 | 407 | 0.997 | 3.88 | 0.0571 | 484 |
| DP087-3-E-F1 | 27,679 | 268 | 0.9971 | 3.46 | 0.0561 | 384 |
| DP087-3-E-F2 | 27,679 | 420 | 0.998 | 4.49 | 0.0263 | 454 |
| DP087-3-E-F3 | 27,679 | 333 | 0.9977 | 3.95 | 0.0419 | 380 |
| DP087-3-E-M1 | 27,679 | 347 | 0.9959 | 3.42 | 0.0757 | 493 |
| DP087-3-E-M2 | 27,679 | 339 | 0.9965 | 3.18 | 0.105 | 467 |
| DP087-3-E-M3 | 27,679 | 267 | 0.9972 | 3.26 | 0.0795 | 351 |
| DP087-10-C-F1 | 27,679 | 457 | 0.9969 | 4.59 | 0.0199 | 540 |
| DP087-10-C-F2 | 27,679 | 304 | 0.9987 | 3.96 | 0.0501 | 320 |
| DP087-10-C-F3 | 27,679 | 425 | 0.9975 | 4.2 | 0.0435 | 485 |
| DP087-10-C-M1 | 27,679 | 418 | 0.9967 | 3.68 | 0.1013 | 495 |
| DP087-10-C-M2 | 27,679 | 416 | 0.9973 | 3.83 | 0.0618 | 482 |
| DP087-10-C-M3 | 27,679 | 409 | 0.9968 | 3.43 | 0.0918 | 505 |
| DP087-10-E-F1 | 27,679 | 435 | 0.9971 | 4.21 | 0.0404 | 503 |
| DP087-10-E-F2 | 27,679 | 453 | 0.9972 | 4.69 | 0.0173 | 551 |
| DP087-10-E-F3 | 27,679 | 407 | 0.9972 | 4.44 | 0.0234 | 486 |
| DP087-10-E-M1 | 27,679 | 404 | 0.9971 | 4.02 | 0.0505 | 490 |
| DP087-10-E-M2 | 27,679 | 425 | 0.9968 | 4.02 | 0.0466 | 523 |
| DP087-10-E-M3 | 27,679 | 418 | 0.9978 | 4.6 | 0.0195 | 467 |
| DP088-3-C-F1 | 27,679 | 390 | 0.9977 | 4.05 | 0.0437 | 473 |
| DP088-3-C-F2 | 27,679 | 398 | 0.9977 | 4.05 | 0.0503 | 467 |
| DP088-3-C-F3 | 27,679 | 468 | 0.9974 | 4.43 | 0.0288 | 520 |
| DP088-3-C-M1 | 27,679 | 347 | 0.9974 | 3.47 | 0.0766 | 405 |
| DP088-3-C-M2 | 27,679 | 432 | 0.997 | 3.89 | 0.0537 | 505 |
| DP088-3-C-M3 | 27,679 | 449 | 0.9967 | 3.93 | 0.0525 | 554 |
| DP088-3-E-F1 | 27,679 | 478 | 0.9969 | 4.76 | 0.0166 | 574 |
| DP088-3-E-F2 | 27,679 | 349 | 0.9972 | 4.15 | 0.0309 | 435 |
| DP088-3-E-F3 | 27,679 | 447 | 0.9977 | 4.77 | 0.0147 | 498 |
| DP088-3-E-M1 | 27,679 | 453 | 0.9972 | 4.25 | 0.0397 | 509 |
| DP088-3-E-M2 | 27,679 | 380 | 0.9964 | 3.35 | 0.106 | 459 |
| DP088-3-E-M3 | 27,679 | 473 | 0.9974 | 4.53 | 0.0315 | 531 |
| DP088-10-C-F1 | 27,679 | 343 | 0.9977 | 3.73 | 0.0555 | 393 |
| DP088-10-C-F2 | 27,679 | 368 | 0.9972 | 3.88 | 0.0495 | 436 |
| DP088-10-C-F3 | 27,679 | 443 | 0.9969 | 3.9 | 0.0477 | 522 |
| DP088-10-C-M1 | 27,679 | 327 | 0.9972 | 3.44 | 0.0803 | 397 |
| DP088-10-C-M2 | 27,679 | 417 | 0.997 | 3.77 | 0.0623 | 521 |
| DP088-10-C-M3 | 27,679 | 388 | 0.9973 | 3.62 | 0.0745 | 444 |
| DP088-10-E-F1 | 27,679 | 445 | 0.9977 | 4.63 | 0.0215 | 503 |
| DP088-10-E-F2 | 27,679 | 414 | 0.9979 | 4.34 | 0.0269 | 457 |
| DP088-10-E-F3 | 27,679 | 299 | 0.9977 | 3.74 | 0.0442 | 386 |
| DP088-10-E-M1 | 27,679 | 413 | 0.9965 | 3.65 | 0.0693 | 512 |
| DP088-10-E-M2 | 27,679 | 265 | 0.9978 | 3.34 | 0.0692 | 330 |
| DP088-10-E-M3 | 27,679 | 388 | 0.9964 | 3.39 | 0.0744 | 487 |
| DP089-3-C-F1 | 27,679 | 388 | 0.9963 | 3.35 | 0.1023 | 464 |
| DP089-3-C-F2 | 27,679 | 384 | 0.997 | 3.59 | 0.0595 | 487 |
| DP089-3-C-F3 | 27,679 | 423 | 0.9954 | 3.43 | 0.0982 | 589 |
| DP089-3-C-M1 | 27,679 | 369 | 0.9979 | 4.05 | 0.0523 | 416 |
| DP089-3-C-M2 | 27,679 | 402 | 0.9973 | 3.73 | 0.0777 | 475 |
| DP089-3-C-M3 | 27,679 | 387 | 0.9972 | 4.29 | 0.0268 | 478 |
| DP089-3-E-M1 | 27,679 | 500 | 0.9955 | 3.85 | 0.0525 | 629 |
| DP089-3-E-M2 | 27,679 | 506 | 0.996 | 3.79 | 0.0596 | 587 |
| DP089-3-E-M3 | 27,679 | 446 | 0.9964 | 4.4 | 0.0272 | 581 |
| DP089-10-C-F1 | 27,679 | 391 | 0.9952 | 3.44 | 0.0841 | 585 |
| DP089-10-C-F2 | 27,679 | 340 | 0.9974 | 3.23 | 0.1146 | 392 |
| DP089-10-C-F3 | 27,679 | 290 | 0.9972 | 3.46 | 0.0729 | 378 |
| DP089-10-C-M1 | 27,679 | 423 | 0.9964 | 3.89 | 0.0546 | 535 |
| DP089-10-C-M2 | 27,679 | 355 | 0.9965 | 3.14 | 0.1241 | 446 |
| DP089-10-C-M3 | 27,679 | 391 | 0.997 | 3.88 | 0.0441 | 466 |
| DP089-10-E-F1 | 27,679 | 453 | 0.9963 | 3.83 | 0.0697 | 573 |
| DP089-10-E-F2 | 27,679 | 491 | 0.9964 | 4.46 | 0.0315 | 634 |
| DP089-10-E-F3 | 27,679 | 543 | 0.9967 | 4.76 | 0.0192 | 616 |
| DP089-10-E-M1 | 27,679 | 402 | 0.9967 | 4.04 | 0.0464 | 476 |
| DP089-10-E-M2 | 27,679 | 416 | 0.9968 | 3.66 | 0.0856 | 501 |
| DP089-10-E-M3 | 27,679 | 466 | 0.9948 | 3.54 | 0.0781 | 638 |
| DP090-3-C-F1 | 27,679 | 359 | 0.9966 | 3.43 | 0.0713 | 460 |
| DP090-3-C-F2 | 27,679 | 407 | 0.9971 | 3.87 | 0.0538 | 473 |
| DP090-3-C-F3 | 27,679 | 352 | 0.9967 | 3.47 | 0.0677 | 431 |
| DP090-3-C-M1 | 27,679 | 427 | 0.9965 | 3.6 | 0.0695 | 506 |
| DP090-3-C-M2 | 27,679 | 316 | 0.9977 | 3.48 | 0.0783 | 371 |
| DP090-3-C-M3 | 27,679 | 379 | 0.9971 | 3.26 | 0.1018 | 441 |
| DP090-3-E-F1 | 27,679 | 302 | 0.998 | 3.48 | 0.1068 | 347 |
| DP090-3-E-F2 | 27,679 | 473 | 0.9975 | 4.69 | 0.0237 | 542 |
| DP090-3-E-F3 | 27,679 | 465 | 0.9967 | 4.3 | 0.039 | 579 |
| DP090-3-E-M1 | 27,679 | 290 | 0.9967 | 2.77 | 0.1523 | 428 |
| DP090-3-E-M2 | 27,679 | 403 | 0.9976 | 4.24 | 0.0333 | 464 |
| DP090-3-E-M3 | 27,679 | 440 | 0.9973 | 4.05 | 0.0524 | 508 |
| DP090-10-C-F1 | 27,679 | 372 | 0.9975 | 3.6 | 0.0667 | 418 |
| DP090-10-C-F2 | 27,679 | 407 | 0.996 | 3.56 | 0.0693 | 534 |
| DP090-10-C-F3 | 27,679 | 309 | 0.9968 | 3.23 | 0.0849 | 405 |
| DP090-10-C-M1 | 27,679 | 307 | 0.9965 | 3.12 | 0.0941 | 408 |
| DP090-10-C-M2 | 27,679 | 291 | 0.9976 | 3.15 | 0.0972 | 343 |
| DP090-10-C-M3 | 27,679 | 365 | 0.9969 | 3.19 | 0.1042 | 452 |
| DP090-10-E-F1 | 27,679 | 379 | 0.9966 | 3.48 | 0.0715 | 468 |
| DP090-10-E-F2 | 27,679 | 409 | 0.9967 | 3.59 | 0.0745 | 492 |
| DP090-10-E-F3 | 27,679 | 254 | 0.9976 | 3.24 | 0.0727 | 328 |
| DP090-10-E-M1 | 27,679 | 374 | 0.9963 | 3.68 | 0.0486 | 475 |
| DP090-10-E-M2 | 27,679 | 380 | 0.997 | 3.69 | 0.052 | 455 |
| DP090-10-E-M3 | 27,679 | 406 | 0.9965 | 3.29 | 0.1005 | 519 |
| DP091-3-C-F1 | 27,679 | 436 | 0.9959 | 3.01 | 0.1875 | 558 |
| DP091-3-C-F2 | 27,679 | 458 | 0.9965 | 3.46 | 0.1063 | 557 |
| DP091-3-C-F3 | 27,679 | 420 | 0.9962 | 3.22 | 0.1314 | 548 |
| DP091-3-C-M1 | 27,679 | 419 | 0.9965 | 3.45 | 0.1097 | 527 |
| DP091-3-C-M2 | 27,679 | 336 | 0.9967 | 2.49 | 0.2472 | 409 |
| DP091-3-C-M3 | 27,679 | 518 | 0.9965 | 4.26 | 0.0482 | 590 |
| DP091-3-E-F1 | 27,679 | 370 | 0.9972 | 3.53 | 0.1246 | 434 |
| DP091-3-E-F2 | 27,679 | 309 | 0.9976 | 3.55 | 0.0831 | 363 |
| DP091-3-E-F3 | 27,679 | 334 | 0.9978 | 4.22 | 0.0305 | 411 |
| DP091-3-E-M1 | 27,679 | 429 | 0.9977 | 4.32 | 0.035 | 487 |
| DP091-3-E-M2 | 27,679 | 407 | 0.9977 | 4.3 | 0.0291 | 455 |
| DP091-3-E-M3 | 27,679 | 409 | 0.9975 | 4.42 | 0.026 | 459 |
| DP091-10-C-F1 | 27,679 | 342 | 0.9964 | 2.56 | 0.2481 | 445 |
| DP091-10-C-F2 | 27,679 | 456 | 0.9969 | 3.43 | 0.1165 | 526 |
| DP091-10-C-F3 | 27,679 | 392 | 0.9964 | 2.79 | 0.1874 | 466 |
| DP091-10-C-M1 | 27,679 | 349 | 0.9954 | 2.35 | 0.2799 | 505 |
| DP091-10-C-M2 | 27,679 | 368 | 0.996 | 2.5 | 0.244 | 479 |
| DP091-10-C-M3 | 27,679 | 494 | 0.9965 | 3.74 | 0.0976 | 577 |
| DP091-10-E-F1 | 27,679 | 345 | 0.9968 | 3.79 | 0.0498 | 482 |
| DP091-10-E-F2 | 27,679 | 321 | 0.9965 | 3.21 | 0.0964 | 418 |
| DP091-10-E-F3 | 27,679 | 370 | 0.9982 | 4.22 | 0.0312 | 397 |
| DP091-10-E-M1 | 27,679 | 410 | 0.9978 | 3.91 | 0.07 | 450 |
| DP091-10-E-M2 | 27,679 | 427 | 0.9976 | 4.38 | 0.0279 | 494 |
| DP091-10-E-M3 | 27,679 | 404 | 0.9971 | 4.12 | 0.0397 | 479 |
| DP092-3-C-F1 | 27,679 | 306 | 0.9979 | 3.25 | 0.1133 | 340 |
| DP092-3-C-F2 | 27,679 | 368 | 0.9982 | 4.32 | 0.0383 | 419 |
| DP092-3-C-F3 | 27,679 | 372 | 0.9976 | 3.85 | 0.0556 | 427 |
| DP092-3-C-M1 | 27,679 | 193 | 0.998 | 3.03 | 0.0773 | 271 |
| DP092-3-C-M2 | 27,679 | 218 | 0.9983 | 3.13 | 0.077 | 255 |
| DP092-3-C-M3 | 27,679 | 266 | 0.9977 | 2.72 | 0.1531 | 336 |
| DP092-3-E-F1 | 27,679 | 186 | 0.9978 | 1.71 | 0.2992 | 251 |
| DP092-3-E-F2 | 27,679 | 205 | 0.9987 | 2.86 | 0.1125 | 233 |
| DP092-3-E-F3 | 27,679 | 295 | 0.9974 | 3.03 | 0.0971 | 375 |
| DP092-3-E-M1 | 27,679 | 289 | 0.9971 | 2.8 | 0.1469 | 405 |
| DP092-3-E-M2 | 27,679 | 139 | 0.9983 | 2 | 0.2271 | 181 |
| DP092-3-E-M3 | 27,679 | 144 | 0.9982 | 1.73 | 0.3576 | 228 |
| DP092-10-C-F1 | 27,679 | 378 | 0.9975 | 3.83 | 0.0626 | 447 |
| DP092-10-C-F2 | 27,679 | 362 | 0.9983 | 4.21 | 0.0332 | 392 |
| DP092-10-C-F3 | 27,679 | 376 | 0.9981 | 3.93 | 0.0639 | 420 |
| DP092-10-C-M1 | 27,679 | 254 | 0.9983 | 3.16 | 0.0886 | 301 |
| DP092-10-C-M2 | 27,679 | 279 | 0.9974 | 3.14 | 0.0853 | 354 |
| DP092-10-C-M3 | 27,679 | 231 | 0.998 | 2.44 | 0.2031 | 270 |
| DP092-10-E-F1 | 27,679 | 331 | 0.9977 | 3.29 | 0.1024 | 380 |
| DP092-10-E-F2 | 27,679 | 265 | 0.9979 | 3.23 | 0.0822 | 339 |
| DP092-10-E-F3 | 27,679 | 283 | 0.998 | 3.15 | 0.0991 | 321 |
| DP092-10-E-M1 | 27,679 | 330 | 0.9977 | 3 | 0.2006 | 386 |
| DP092-10-E-M2 | 27,679 | 237 | 0.9971 | 2.43 | 0.1957 | 322 |
| DP092-10-E-M3 | 27,679 | 269 | 0.9979 | 2.74 | 0.153 | 326 |
| DP093-3-C-F1 | 27,679 | 520 | 0.9958 | 3.82 | 0.0734 | 611 |
| DP093-3-C-F2 | 27,679 | 392 | 0.9959 | 3.41 | 0.0815 | 532 |
| DP093-3-C-F3 | 27,679 | 393 | 0.9961 | 3.39 | 0.0822 | 502 |
| DP093-3-C-M1 | 27,679 | 299 | 0.9977 | 3.45 | 0.0793 | 360 |
| DP093-3-C-M2 | 27,679 | 279 | 0.9971 | 3.18 | 0.1037 | 404 |
| DP093-3-C-M3 | 27,679 | 401 | 0.9966 | 3.64 | 0.0708 | 496 |
| DP093-3-E-F1 | 27,679 | 318 | 0.9975 | 3.04 | 0.1127 | 370 |
| DP093-3-E-F2 | 27,679 | 356 | 0.9971 | 3.52 | 0.0756 | 461 |
| DP093-3-E-F3 | 27,679 | 378 | 0.9971 | 3.59 | 0.0785 | 457 |
| DP093-3-E-M1 | 27,679 | 376 | 0.997 | 4.06 | 0.044 | 477 |
| DP093-3-E-M2 | 27,679 | 395 | 0.9969 | 4.03 | 0.0405 | 486 |
| DP093-3-E-M3 | 27,679 | 363 | 0.9969 | 3.58 | 0.0776 | 444 |
| DP093-10-C-F1 | 27,679 | 375 | 0.9966 | 3.39 | 0.0966 | 519 |
| DP093-10-C-F2 | 27,679 | 249 | 0.9978 | 3.28 | 0.1059 | 329 |
| DP093-10-C-F3 | 27,679 | 447 | 0.9961 | 3.61 | 0.0779 | 537 |
| DP093-10-C-M1 | 27,679 | 363 | 0.9967 | 3.24 | 0.1081 | 454 |
| DP093-10-C-M2 | 27,679 | 299 | 0.9983 | 3.84 | 0.0468 | 329 |
| DP093-10-C-M3 | 27,679 | 273 | 0.9981 | 3.68 | 0.048 | 313 |
| DP093-10-E-F1 | 27,679 | 350 | 0.9975 | 2.82 | 0.1901 | 397 |
| DP093-10-E-F2 | 27,679 | 407 | 0.9978 | 4.6 | 0.0219 | 459 |
| DP093-10-E-F3 | 27,679 | 287 | 0.997 | 3.07 | 0.0983 | 368 |
| DP093-10-E-M1 | 27,679 | 304 | 0.9979 | 2.78 | 0.2042 | 342 |
| DP093-10-E-M2 | 27,679 | 415 | 0.9973 | 4.52 | 0.026 | 513 |
| DP093-10-E-M3 | 27,679 | 364 | 0.9971 | 4.22 | 0.0335 | 518 |
| DP094-3-C-F1 | 27,679 | 243 | 0.997 | 2.46 | 0.1864 | 344 |
| DP094-3-C-F2 | 27,679 | 215 | 0.9973 | 1.94 | 0.3797 | 290 |
| DP094-3-C-F3 | 27,679 | 208 | 0.9976 | 2.37 | 0.1766 | 277 |
| DP094-3-C-M1 | 27,679 | 285 | 0.9971 | 2.77 | 0.1616 | 378 |
| DP094-3-C-M2 | 27,679 | 290 | 0.9972 | 1.76 | 0.4449 | 365 |
| DP094-3-C-M3 | 27,679 | 315 | 0.997 | 2.84 | 0.1985 | 407 |
| DP094-3-E-F1 | 27,679 | 289 | 0.997 | 2.57 | 0.1608 | 384 |
| DP094-3-E-F2 | 27,679 | 234 | 0.9966 | 2.26 | 0.1999 | 338 |
| DP094-3-E-F3 | 27,679 | 346 | 0.9977 | 3.34 | 0.1094 | 390 |
| DP094-3-E-M1 | 27,679 | 268 | 0.9976 | 2.48 | 0.2032 | 323 |
| DP094-3-E-M2 | 27,679 | 309 | 0.9973 | 2.83 | 0.1165 | 365 |
| DP094-3-E-M3 | 27,679 | 308 | 0.9977 | 3.17 | 0.0833 | 360 |
| DP094-10-C-F1 | 27,679 | 435 | 0.9975 | 4.76 | 0.0164 | 488 |
| DP094-10-C-F2 | 27,679 | 474 | 0.9958 | 4.05 | 0.0518 | 593 |
| DP094-10-C-F3 | 27,679 | 457 | 0.9968 | 4.44 | 0.0308 | 566 |
| DP094-10-C-M1 | 27,679 | 308 | 0.9975 | 3.76 | 0.0463 | 363 |
| DP094-10-C-M2 | 27,679 | 420 | 0.9971 | 4.14 | 0.0464 | 495 |
| DP094-10-C-M3 | 27,679 | 292 | 0.9975 | 3.64 | 0.0564 | 354 |
| DP094-10-E-F1 | 27,679 | 347 | 0.9967 | 2.99 | 0.1179 | 429 |
| DP094-10-E-F2 | 27,679 | 259 | 0.9964 | 2.6 | 0.1366 | 397 |
| DP094-10-E-F3 | 27,679 | 219 | 0.9974 | 2.38 | 0.1985 | 308 |
| DP094-10-E-M1 | 27,679 | 222 | 0.9976 | 2.64 | 0.1354 | 282 |
| DP094-10-E-M2 | 27,679 | 342 | 0.9971 | 3.77 | 0.0516 | 437 |
| DP094-10-E-M3 | 27,679 | 412 | 0.9964 | 4.31 | 0.0271 | 535 |
| DP095-3-C-F1 | 27,679 | 406 | 0.996 | 3.08 | 0.1159 | 515 |
| DP095-3-C-F2 | 27,679 | 330 | 0.9967 | 3.18 | 0.0805 | 425 |
| DP095-3-C-F3 | 27,679 | 452 | 0.9968 | 3.71 | 0.061 | 522 |
| DP095-3-C-M1 | 27,679 | 498 | 0.9964 | 4.32 | 0.0316 | 593 |
| DP095-3-C-M2 | 27,679 | 478 | 0.9965 | 3.77 | 0.0829 | 556 |
| DP095-3-C-M3 | 27,679 | 497 | 0.9967 | 4.21 | 0.0471 | 572 |
| DP095-3-E-F1 | 27,679 | 390 | 0.9968 | 3.95 | 0.0406 | 499 |
| DP095-3-E-F2 | 27,679 | 421 | 0.9979 | 4.51 | 0.0217 | 463 |
| DP095-3-E-F3 | 27,679 | 389 | 0.997 | 3.76 | 0.0669 | 474 |
| DP095-3-E-M1 | 27,679 | 421 | 0.9977 | 4.12 | 0.0366 | 469 |
| DP095-3-E-M2 | 27,679 | 293 | 0.9979 | 3.66 | 0.0535 | 343 |
| DP095-3-E-M3 | 27,679 | 244 | 0.9971 | 3 | 0.1121 | 356 |
| DP095-10-C-F1 | 27,679 | 443 | 0.997 | 3.59 | 0.079 | 517 |
| DP095-10-C-F2 | 27,679 | 312 | 0.9969 | 2.96 | 0.0956 | 393 |
| DP095-10-C-F3 | 27,679 | 457 | 0.9971 | 3.89 | 0.0496 | 516 |
| DP095-10-C-M1 | 27,679 | 438 | 0.9964 | 3.74 | 0.064 | 513 |
| DP095-10-C-M2 | 27,679 | 485 | 0.9967 | 3.47 | 0.1452 | 567 |
| DP095-10-C-M3 | 27,679 | 460 | 0.9965 | 4.16 | 0.0393 | 557 |
| DP095-10-E-F1 | 27,679 | 333 | 0.9979 | 4.16 | 0.0335 | 392 |
| DP095-10-E-F2 | 27,679 | 359 | 0.9968 | 3.36 | 0.0765 | 432 |
| DP095-10-E-F3 | 27,679 | 381 | 0.9973 | 3.7 | 0.0603 | 447 |
| DP095-10-E-M1 | 27,679 | 428 | 0.997 | 4.33 | 0.0286 | 515 |
| DP095-10-E-M2 | 27,679 | 349 | 0.9971 | 2.85 | 0.2052 | 411 |
| DP095-10-E-M3 | 27,679 | 328 | 0.9978 | 4.07 | 0.0364 | 384 |
| DP096-3-C-F1 | 27,679 | 409 | 0.9971 | 4.33 | 0.0434 | 556 |
| DP096-3-C-F2 | 27,679 | 327 | 0.9972 | 3.01 | 0.1656 | 400 |
| DP096-3-C-F3 | 27,679 | 410 | 0.9971 | 3.76 | 0.0866 | 479 |
| DP096-3-C-M1 | 27,679 | 233 | 0.9978 | 2.86 | 0.1272 | 306 |
| DP096-3-C-M2 | 27,679 | 188 | 0.9983 | 2.7 | 0.1288 | 225 |
| DP096-3-C-M3 | 27,679 | 345 | 0.998 | 4.07 | 0.0435 | 386 |
| DP096-3-E-F1 | 27,679 | 387 | 0.9976 | 4.15 | 0.0388 | 450 |
| DP096-3-E-F2 | 27,679 | 330 | 0.9978 | 3.27 | 0.1239 | 391 |
| DP096-3-E-F3 | 27,679 | 404 | 0.9977 | 4.61 | 0.0215 | 463 |
| DP096-3-E-M1 | 27,679 | 340 | 0.9977 | 2.69 | 0.3056 | 405 |
| DP096-3-E-M2 | 27,679 | 346 | 0.9968 | 3.03 | 0.118 | 447 |
| DP096-3-E-M3 | 27,679 | 388 | 0.9979 | 3.75 | 0.1199 | 426 |
| DP096-10-C-F1 | 27,679 | 392 | 0.997 | 3.92 | 0.0589 | 479 |
| DP096-10-C-F2 | 27,679 | 375 | 0.9967 | 3.03 | 0.2 | 480 |
| DP096-10-C-F3 | 27,679 | 430 | 0.996 | 4.12 | 0.0406 | 562 |
| DP096-10-E-F1 | 27,679 | 326 | 0.9979 | 3.43 | 0.0782 | 370 |
| DP096-10-E-F2 | 27,679 | 382 | 0.9978 | 4.06 | 0.0571 | 431 |
| DP096-10-E-F3 | 27,679 | 362 | 0.997 | 2.85 | 0.1778 | 420 |
| DP096-10-E-M1 | 27,679 | 369 | 0.9971 | 3.69 | 0.0559 | 444 |
| DP096-10-E-M2 | 27,679 | 424 | 0.9974 | 3.98 | 0.056 | 483 |
| DP096-10-E-M3 | 27,679 | 409 | 0.9969 | 3.53 | 0.1156 | 494 |
| DP097-3-C-F1 | 27,679 | 318 | 0.9981 | 3.78 | 0.0592 | 381 |
| DP097-3-C-F2 | 27,679 | 358 | 0.9983 | 4.45 | 0.0217 | 384 |
| DP097-3-C-F3 | 27,679 | 317 | 0.998 | 4.04 | 0.0426 | 372 |
| DP097-3-C-M1 | 27,679 | 407 | 0.9978 | 4.37 | 0.0272 | 456 |
| DP097-3-C-M2 | 27,679 | 381 | 0.9969 | 3.6 | 0.0758 | 491 |
| DP097-3-C-M3 | 27,679 | 369 | 0.9978 | 3.95 | 0.0447 | 428 |
| DP097-3-E-F1 | 27,679 | 416 | 0.9973 | 4.37 | 0.0266 | 481 |
| DP097-3-E-F2 | 27,679 | 332 | 0.9981 | 3.75 | 0.0936 | 363 |
| DP097-3-E-F3 | 27,679 | 325 | 0.998 | 4.06 | 0.0371 | 371 |
| DP097-3-E-M1 | 27,679 | 247 | 0.9978 | 3.32 | 0.0812 | 315 |
| DP097-3-E-M2 | 27,679 | 280 | 0.9974 | 2.91 | 0.1166 | 360 |
| DP097-3-E-M3 | 27,679 | 355 | 0.9969 | 3.33 | 0.0774 | 427 |
| DP097-10-C-F1 | 27,679 | 273 | 0.9974 | 3.03 | 0.1066 | 370 |
| DP097-10-C-F2 | 27,679 | 284 | 0.9981 | 3.79 | 0.0442 | 333 |
| DP097-10-C-F3 | 27,679 | 351 | 0.9975 | 4.08 | 0.0501 | 422 |
| DP097-10-C-M1 | 27,679 | 364 | 0.997 | 3.93 | 0.0363 | 429 |
| DP097-10-C-M2 | 27,679 | 358 | 0.9971 | 3.82 | 0.0535 | 433 |
| DP097-10-C-M3 | 27,679 | 350 | 0.9969 | 3.68 | 0.0507 | 426 |
| DP097-10-E-F1 | 27,679 | 335 | 0.998 | 4.36 | 0.0236 | 386 |
| DP097-10-E-F2 | 27,679 | 321 | 0.9978 | 3.62 | 0.0581 | 375 |
| DP097-10-E-F3 | 27,679 | 322 | 0.9984 | 4.05 | 0.0365 | 349 |
| DP097-10-E-M1 | 27,679 | 417 | 0.9967 | 4.04 | 0.0467 | 508 |
| DP097-10-E-M2 | 27,679 | 318 | 0.9968 | 3.35 | 0.0626 | 395 |
| DP097-10-E-M3 | 27,679 | 267 | 0.9975 | 3.52 | 0.052 | 338 |
| DP098-3-C-F1 | 27,679 | 320 | 0.9961 | 2.45 | 0.1961 | 454 |
| DP098-3-C-F2 | 27,679 | 452 | 0.997 | 3.91 | 0.0513 | 526 |
| DP098-3-C-F3 | 27,679 | 453 | 0.9957 | 3.48 | 0.0743 | 563 |
| DP098-3-C-M1 | 27,679 | 399 | 0.9969 | 3.78 | 0.0677 | 477 |
| DP098-3-C-M2 | 27,679 | 495 | 0.996 | 4.2 | 0.0475 | 647 |
| DP098-3-C-M3 | 27,679 | 500 | 0.9967 | 4.41 | 0.0366 | 569 |
| DP098-3-E-F1 | 27,679 | 285 | 0.9973 | 3.19 | 0.0814 | 358 |
| DP098-3-E-F2 | 27,679 | 283 | 0.9971 | 3.2 | 0.0845 | 360 |
| DP098-3-E-F3 | 27,679 | 180 | 0.9979 | 2.2 | 0.2683 | 283 |
| DP098-3-E-M1 | 27,679 | 264 | 0.9975 | 2.89 | 0.1295 | 324 |
| DP098-3-E-M2 | 27,679 | 265 | 0.9976 | 2.96 | 0.1099 | 320 |
| DP098-3-E-M3 | 27,679 | 230 | 0.9975 | 2.63 | 0.1386 | 303 |
| DP098-10-C-F1 | 27,679 | 477 | 0.996 | 3.42 | 0.1084 | 577 |
| DP098-10-C-F2 | 27,679 | 430 | 0.9964 | 3.65 | 0.0632 | 540 |
| DP098-10-C-F3 | 27,679 | 408 | 0.9969 | 3.37 | 0.0807 | 476 |
| DP098-10-C-M1 | 27,679 | 399 | 0.9966 | 3.53 | 0.0897 | 498 |
| DP098-10-C-M2 | 27,679 | 454 | 0.996 | 3.51 | 0.1073 | 572 |
| DP098-10-C-M3 | 27,679 | 457 | 0.9968 | 3.98 | 0.0546 | 530 |
| DP098-10-E-F1 | 27,679 | 344 | 0.998 | 3.98 | 0.0442 | 397 |
| DP098-10-E-F2 | 27,679 | 236 | 0.9977 | 2.13 | 0.2862 | 313 |
| DP098-10-E-F3 | 27,679 | 297 | 0.9977 | 2.77 | 0.2087 | 362 |
| DP098-10-E-M1 | 27,679 | 285 | 0.9976 | 2.36 | 0.2486 | 335 |
| DP098-10-E-M2 | 27,679 | 349 | 0.9968 | 3.3 | 0.1044 | 462 |
| DP098-10-E-M3 | 27,679 | 239 | 0.9982 | 3.34 | 0.077 | 288 |
| DP099-3-C-F1 | 27,679 | 349 | 0.9971 | 3.53 | 0.0756 | 430 |
| DP099-3-C-F2 | 27,679 | 271 | 0.9986 | 3 | 0.1071 | 293 |
| DP099-3-C-F3 | 27,679 | 313 | 0.9976 | 3.34 | 0.0906 | 367 |
| DP099-3-C-M1 | 27,679 | 252 | 0.9978 | 3.04 | 0.0946 | 309 |
| DP099-3-C-M2 | 27,679 | 261 | 0.9973 | 2.97 | 0.1165 | 360 |
| DP099-3-C-M3 | 27,679 | 259 | 0.998 | 3.01 | 0.1036 | 323 |
| DP099-3-E-F1 | 27,679 | 340 | 0.9977 | 3.36 | 0.1007 | 405 |
| DP099-3-E-F2 | 27,679 | 341 | 0.9981 | 3.54 | 0.0947 | 380 |
| DP099-3-E-F3 | 27,679 | 323 | 0.9981 | 3.55 | 0.0749 | 369 |
| DP099-3-E-M1 | 27,679 | 203 | 0.9981 | 2.86 | 0.1108 | 256 |
| DP099-3-E-M2 | 27,679 | 179 | 0.9979 | 2.78 | 0.1072 | 266 |
| DP099-3-E-M3 | 27,679 | 203 | 0.9983 | 3.05 | 0.0956 | 248 |
| DP099-10-C-F1 | 27,679 | 293 | 0.9976 | 3.29 | 0.0813 | 398 |
| DP099-10-C-F2 | 27,679 | 287 | 0.9974 | 3.18 | 0.0889 | 369 |
| DP099-10-C-F3 | 27,679 | 281 | 0.9983 | 3.49 | 0.0648 | 315 |
| DP099-10-C-M1 | 27,679 | 306 | 0.9985 | 3.68 | 0.0758 | 342 |
| DP099-10-C-M2 | 27,679 | 304 | 0.9978 | 3.12 | 0.1049 | 369 |
| DP099-10-C-M3 | 27,679 | 319 | 0.9978 | 3.43 | 0.1121 | 367 |
| DP099-10-E-F1 | 27,679 | 334 | 0.9981 | 3.48 | 0.0956 | 377 |
| DP099-10-E-F2 | 27,679 | 300 | 0.9983 | 3.73 | 0.0567 | 347 |
| DP099-10-E-F3 | 27,679 | 301 | 0.9984 | 3.79 | 0.0529 | 339 |
| DP099-10-E-M1 | 27,679 | 260 | 0.9977 | 2.8 | 0.1414 | 311 |
| DP099-10-E-M2 | 27,679 | 281 | 0.9972 | 2.94 | 0.1226 | 372 |
| DP099-10-E-M3 | 27,679 | 275 | 0.9982 | 3.2 | 0.1018 | 309 |
| DP101-3-C-F1 | 27,679 | 393 | 0.9958 | 2.78 | 0.1217 | 494 |
| DP101-3-C-F2 | 27,679 | 445 | 0.9949 | 2.9 | 0.1335 | 595 |
| DP101-3-C-F3 | 27,679 | 447 | 0.9957 | 3.88 | 0.0419 | 555 |
| DP101-3-C-M1 | 27,679 | 429 | 0.9968 | 3.32 | 0.124 | 497 |
| DP101-3-C-M2 | 27,679 | 353 | 0.9966 | 2.74 | 0.1552 | 460 |
| DP101-3-C-M3 | 27,679 | 380 | 0.9962 | 2.88 | 0.1565 | 462 |
| DP101-3-E-F1 | 27,679 | 357 | 0.9968 | 3.73 | 0.045 | 450 |
| DP101-3-E-F2 | 27,679 | 204 | 0.9974 | 1.83 | 0.2217 | 270 |
| DP101-3-E-F3 | 27,679 | 334 | 0.9969 | 2.91 | 0.1478 | 419 |
| DP101-3-E-M1 | 27,679 | 492 | 0.9965 | 4.04 | 0.059 | 578 |
| DP101-3-E-M2 | 27,679 | 349 | 0.996 | 3.23 | 0.0839 | 466 |
| DP101-3-E-M3 | 27,679 | 572 | 0.996 | 4.51 | 0.0327 | 687 |
| DP101-10-C-F1 | 27,679 | 421 | 0.9959 | 3.39 | 0.0837 | 538 |
| DP101-10-C-F2 | 27,679 | 340 | 0.9967 | 3.58 | 0.0641 | 448 |
| DP101-10-C-F3 | 27,679 | 313 | 0.9971 | 3.48 | 0.0683 | 379 |
| DP101-10-C-M1 | 27,679 | 441 | 0.9967 | 3.68 | 0.0608 | 507 |
| DP101-10-C-M2 | 27,679 | 348 | 0.996 | 3.21 | 0.0923 | 478 |
| DP101-10-C-M3 | 27,679 | 452 | 0.9944 | 3.33 | 0.0872 | 668 |
| DP101-10-E-F1 | 27,679 | 458 | 0.997 | 4.04 | 0.0425 | 537 |
| DP101-10-E-F2 | 27,679 | 312 | 0.9964 | 3.06 | 0.1085 | 417 |
| DP101-10-E-F3 | 27,679 | 377 | 0.9961 | 3.3 | 0.0796 | 473 |
| DP101-10-E-M1 | 27,679 | 340 | 0.9965 | 2.74 | 0.1548 | 435 |
| DP101-10-E-M2 | 27,679 | 257 | 0.9969 | 2.63 | 0.1542 | 376 |
| DP101-10-E-M3 | 27,679 | 339 | 0.997 | 2.98 | 0.1432 | 455 |
| DP102-3-C-F1 | 27,679 | 362 | 0.9958 | 3.1 | 0.1038 | 513 |
| DP102-3-C-F2 | 27,679 | 308 | 0.9965 | 2.78 | 0.1709 | 424 |
| DP102-3-C-F3 | 27,679 | 354 | 0.9962 | 2.9 | 0.1366 | 457 |
| DP102-3-C-M1 | 27,679 | 360 | 0.9965 | 3.22 | 0.1042 | 474 |
| DP102-3-C-M2 | 27,679 | 395 | 0.9959 | 3.42 | 0.0966 | 549 |
| DP102-3-C-M3 | 27,679 | 314 | 0.9965 | 3.07 | 0.1167 | 434 |
| DP102-3-E-F1 | 27,679 | 526 | 0.9965 | 4.41 | 0.0326 | 608 |
| DP102-3-E-F2 | 27,679 | 388 | 0.9971 | 4.04 | 0.045 | 465 |
| DP102-3-E-F3 | 27,679 | 538 | 0.9963 | 4.36 | 0.0329 | 630 |
| DP102-3-E-M1 | 27,679 | 441 | 0.9959 | 3.53 | 0.0824 | 550 |
| DP102-3-E-M2 | 27,679 | 414 | 0.9953 | 3.03 | 0.1908 | 581 |
| DP102-3-E-M3 | 27,679 | 254 | 0.9969 | 2.91 | 0.1675 | 362 |
| DP102-10-C-F1 | 27,679 | 437 | 0.9964 | 4.02 | 0.0406 | 529 |
| DP102-10-C-F2 | 27,679 | 455 | 0.996 | 4.24 | 0.0267 | 551 |
| DP102-10-C-F3 | 27,679 | 208 | 0.9978 | 3.29 | 0.0738 | 276 |
| DP102-10-C-M1 | 27,679 | 464 | 0.996 | 3.98 | 0.0436 | 577 |
| DP102-10-C-M2 | 27,679 | 476 | 0.9961 | 3.82 | 0.0535 | 561 |
| DP102-10-C-M3 | 27,679 | 453 | 0.9955 | 3.89 | 0.0488 | 611 |
| DP102-10-E-F1 | 27,679 | 303 | 0.9971 | 3.54 | 0.0652 | 402 |
| DP102-10-E-F2 | 27,679 | 431 | 0.9969 | 4.22 | 0.0329 | 516 |
| DP102-10-E-F3 | 27,679 | 451 | 0.9962 | 4.22 | 0.0332 | 572 |
| DP102-10-E-M1 | 27,679 | 465 | 0.9963 | 4.16 | 0.041 | 562 |
| DP102-10-E-M2 | 27,679 | 438 | 0.9964 | 3.95 | 0.0526 | 522 |
| DP102-10-E-M3 | 27,679 | 344 | 0.9962 | 3.82 | 0.0443 | 517 |
| DP104-3-C-F1 | 27,679 | 414 | 0.9966 | 3.41 | 0.0766 | 495 |
| DP104-3-C-F2 | 27,679 | 351 | 0.9967 | 2.87 | 0.1283 | 453 |
| DP104-3-C-F3 | 27,679 | 412 | 0.9961 | 3.39 | 0.0776 | 538 |
| DP104-3-C-M1 | 27,679 | 269 | 0.9974 | 2.71 | 0.1494 | 340 |
| DP104-3-C-M2 | 27,679 | 431 | 0.9964 | 3.36 | 0.1199 | 520 |
| DP104-3-C-M3 | 27,679 | 523 | 0.9963 | 3.68 | 0.1309 | 620 |
| DP104-3-E-F1 | 27,679 | 240 | 0.9967 | 1.74 | 0.4084 | 374 |
| DP104-3-E-F2 | 27,679 | 318 | 0.9967 | 3.48 | 0.0719 | 458 |
| DP104-3-E-F3 | 27,679 | 270 | 0.9971 | 3.18 | 0.0949 | 366 |
| DP104-3-E-M1 | 27,679 | 312 | 0.9962 | 2.96 | 0.121 | 436 |
| DP104-3-E-M2 | 27,679 | 402 | 0.9963 | 4.04 | 0.0376 | 519 |
| DP104-3-E-M3 | 27,679 | 436 | 0.9965 | 3.81 | 0.0601 | 504 |
| DP104-10-C-F1 | 27,679 | 280 | 0.9968 | 2.87 | 0.1102 | 393 |
| DP104-10-C-F2 | 27,679 | 305 | 0.9972 | 2.74 | 0.1386 | 368 |
| DP104-10-C-F3 | 27,679 | 413 | 0.9971 | 3.46 | 0.0836 | 467 |
| DP104-10-C-M1 | 27,679 | 420 | 0.9961 | 2.91 | 0.2118 | 527 |
| DP104-10-C-M2 | 27,679 | 484 | 0.9964 | 3.82 | 0.0767 | 569 |
| DP104-10-C-M3 | 27,679 | 467 | 0.9951 | 3.46 | 0.1028 | 626 |
| DP104-10-E-F1 | 27,679 | 379 | 0.9969 | 3.29 | 0.1241 | 454 |
| DP104-10-E-F2 | 27,679 | 375 | 0.9964 | 2.98 | 0.1279 | 460 |
| DP104-10-E-F3 | 27,679 | 203 | 0.9978 | 2.55 | 0.1443 | 289 |
| DP104-10-E-M1 | 27,679 | 445 | 0.9971 | 3.94 | 0.0546 | 500 |
| DP104-10-E-M2 | 27,679 | 233 | 0.9972 | 2.76 | 0.1438 | 321 |
| DP104-10-E-M3 | 27,679 | 386 | 0.9966 | 2.85 | 0.158 | 452 |
| DP105-3-C-F1 | 27,679 | 356 | 0.9965 | 2.89 | 0.1223 | 457 |
| DP105-3-C-F2 | 27,679 | 339 | 0.9963 | 2.96 | 0.1253 | 453 |
| DP105-3-C-F3 | 27,679 | 422 | 0.9962 | 3.79 | 0.0566 | 529 |
| DP105-3-C-M1 | 27,679 | 375 | 0.9959 | 3.15 | 0.1377 | 504 |
| DP105-3-C-M2 | 27,679 | 456 | 0.9964 | 3.53 | 0.0784 | 533 |
| DP105-3-C-M3 | 27,679 | 502 | 0.9965 | 3.51 | 0.1233 | 578 |
| DP105-3-E-F1 | 27,679 | 296 | 0.9978 | 2.97 | 0.111 | 344 |
| DP105-3-E-F2 | 27,679 | 344 | 0.9979 | 3.6 | 0.0765 | 399 |
| DP105-3-E-F3 | 27,679 | 345 | 0.9979 | 4.1 | 0.0426 | 434 |
| DP105-3-E-M1 | 27,679 | 556 | 0.996 | 4.37 | 0.0327 | 669 |
| DP105-3-E-M2 | 27,679 | 560 | 0.9958 | 4.64 | 0.0268 | 691 |
| DP105-3-E-M3 | 27,679 | 529 | 0.9963 | 4.37 | 0.0353 | 620 |
| DP105-10-C-F1 | 27,679 | 346 | 0.9969 | 2.88 | 0.1303 | 417 |
| DP105-10-C-F2 | 27,679 | 421 | 0.9962 | 3.3 | 0.1173 | 524 |
| DP105-10-C-F3 | 27,679 | 477 | 0.9964 | 3.87 | 0.0569 | 564 |
| DP105-10-C-M1 | 27,679 | 316 | 0.9963 | 2.67 | 0.2194 | 430 |
| DP105-10-C-M2 | 27,679 | 468 | 0.9962 | 3.46 | 0.0911 | 559 |
| DP105-10-C-M3 | 27,679 | 488 | 0.9966 | 3.09 | 0.1797 | 553 |
| DP105-10-E-F1 | 27,679 | 308 | 0.9976 | 3.32 | 0.0951 | 371 |
| DP105-10-E-F2 | 27,679 | 284 | 0.9974 | 3.28 | 0.0728 | 346 |
| DP105-10-E-F3 | 27,679 | 560 | 0.9956 | 4.19 | 0.0436 | 687 |
| DP105-10-E-M1 | 27,679 | 354 | 0.9964 | 3.28 | 0.0802 | 464 |
| DP105-10-E-M2 | 27,679 | 322 | 0.9978 | 3.47 | 0.0704 | 365 |
| DP105-10-E-M3 | 27,679 | 257 | 0.9975 | 3.51 | 0.062 | 332 |
| DP117-3-C-F1 | 27,679 | 455 | 0.9953 | 3.89 | 0.0517 | 600 |
| DP117-3-C-F2 | 27,679 | 457 | 0.9963 | 4.01 | 0.0531 | 564 |
| DP117-3-C-F3 | 27,679 | 396 | 0.9957 | 3.26 | 0.1154 | 543 |
| DP117-3-C-M1 | 27,679 | 456 | 0.9965 | 4.1 | 0.0439 | 583 |
| DP117-3-C-M2 | 27,679 | 453 | 0.9967 | 4.25 | 0.0356 | 530 |
| DP117-3-C-M3 | 27,679 | 444 | 0.9959 | 3.23 | 0.1171 | 540 |
| DP117-3-E-F1 | 27,679 | 352 | 0.997 | 3.61 | 0.0997 | 421 |
| DP117-3-E-F2 | 27,679 | 473 | 0.997 | 4.44 | 0.0384 | 585 |
| DP117-3-E-F3 | 27,679 | 458 | 0.9967 | 4.64 | 0.0221 | 598 |
| DP117-3-E-M1 | 27,679 | 444 | 0.997 | 4.16 | 0.0471 | 523 |
| DP117-3-E-M2 | 27,679 | 437 | 0.9972 | 3.97 | 0.074 | 494 |
| DP117-3-E-M3 | 27,679 | 481 | 0.997 | 4.45 | 0.0302 | 549 |
| DP117-10-C-F1 | 27,679 | 454 | 0.997 | 4.31 | 0.0295 | 510 |
| DP117-10-C-F2 | 27,679 | 479 | 0.9967 | 4.62 | 0.0205 | 574 |
| DP117-10-C-F3 | 27,679 | 427 | 0.9957 | 3.67 | 0.0792 | 544 |
| DP117-10-C-M1 | 27,679 | 477 | 0.9966 | 4.17 | 0.0395 | 558 |
| DP117-10-C-M2 | 27,679 | 486 | 0.9973 | 4.61 | 0.0223 | 530 |
| DP117-10-C-M3 | 27,679 | 469 | 0.996 | 4.23 | 0.0293 | 578 |
| DP117-10-E-F1 | 27,679 | 359 | 0.9976 | 3.76 | 0.0603 | 405 |
| DP117-10-E-F2 | 27,679 | 371 | 0.9963 | 3.54 | 0.1075 | 500 |
| DP117-10-E-F3 | 27,679 | 459 | 0.9964 | 4.42 | 0.0258 | 553 |
| DP117-10-E-M1 | 27,679 | 467 | 0.997 | 4.42 | 0.0319 | 546 |
| DP117-10-E-M2 | 27,679 | 386 | 0.9967 | 4.02 | 0.0376 | 483 |
| DP117-10-E-M3 | 27,679 | 435 | 0.9966 | 3.99 | 0.0527 | 519 |
| DP118-3-C-F1 | 27,679 | 480 | 0.9967 | 4.14 | 0.0489 | 567 |
| DP118-3-C-F2 | 27,679 | 541 | 0.9957 | 4.26 | 0.0333 | 649 |
| DP118-3-C-F3 | 27,679 | 545 | 0.9966 | 4.3 | 0.0579 | 618 |
| DP118-3-C-M1 | 27,679 | 505 | 0.9968 | 3.52 | 0.1119 | 570 |
| DP118-3-C-M2 | 27,679 | 289 | 0.9965 | 2.14 | 0.2597 | 395 |
| DP118-3-C-M3 | 27,679 | 293 | 0.9965 | 2.34 | 0.1987 | 388 |
| DP118-3-E-F1 | 27,679 | 452 | 0.9973 | 4.66 | 0.0196 | 516 |
| DP118-3-E-F2 | 27,679 | 375 | 0.9976 | 4.36 | 0.0286 | 454 |
| DP118-3-E-F3 | 27,679 | 357 | 0.9979 | 4.03 | 0.0342 | 407 |
| DP118-3-E-M1 | 27,679 | 303 | 0.9974 | 2.35 | 0.3252 | 372 |
| DP118-3-E-M2 | 27,679 | 232 | 0.997 | 2.78 | 0.1607 | 332 |
| DP118-3-E-M3 | 27,679 | 453 | 0.9974 | 4.62 | 0.0209 | 519 |
| DP118-10-C-F1 | 27,679 | 481 | 0.9969 | 4.29 | 0.0391 | 560 |
| DP118-10-C-F2 | 27,679 | 439 | 0.9956 | 3.53 | 0.0811 | 575 |
| DP118-10-C-F3 | 27,679 | 454 | 0.9958 | 3.18 | 0.1738 | 567 |
| DP118-10-C-M1 | 27,679 | 458 | 0.9962 | 2.93 | 0.1796 | 555 |
| DP118-10-C-M2 | 27,679 | 199 | 0.9972 | 1.91 | 0.2686 | 330 |
| DP118-10-C-M3 | 27,679 | 324 | 0.996 | 2.38 | 0.2067 | 457 |
| DP118-10-E-F1 | 27,679 | 223 | 0.9974 | 2.35 | 0.1909 | 315 |
| DP118-10-E-F2 | 27,679 | 410 | 0.9968 | 3.85 | 0.0623 | 497 |
| DP118-10-E-M1 | 27,679 | 328 | 0.9973 | 3.14 | 0.1575 | 396 |
| DP118-10-E-M2 | 27,679 | 446 | 0.9973 | 4.32 | 0.0317 | 505 |
| DP118-10-E-M3 | 27,679 | 395 | 0.9975 | 4.34 | 0.0293 | 464 |
| DP119-3-C-F1 | 27,679 | 530 | 0.997 | 4.55 | 0.0361 | 591 |
| DP119-3-C-F2 | 27,679 | 477 | 0.9967 | 4.1 | 0.0571 | 543 |
| DP119-3-C-F3 | 27,679 | 473 | 0.9959 | 3.69 | 0.063 | 561 |
| DP119-3-C-M1 | 27,679 | 250 | 0.9965 | 2.07 | 0.2602 | 379 |
| DP119-3-C-M2 | 27,679 | 499 | 0.997 | 3.74 | 0.0898 | 569 |
| DP119-3-C-M3 | 27,679 | 443 | 0.996 | 3.2 | 0.1354 | 552 |
| DP119-3-E-F1 | 27,679 | 277 | 0.9976 | 2.81 | 0.1662 | 363 |
| DP119-3-E-F2 | 27,679 | 251 | 0.9979 | 1.99 | 0.427 | 300 |
| DP119-3-E-F3 | 27,679 | 276 | 0.9977 | 2.36 | 0.2827 | 330 |
| DP119-3-E-M1 | 27,679 | 342 | 0.9978 | 2.87 | 0.172 | 378 |
| DP119-3-E-M2 | 27,679 | 351 | 0.9978 | 3.2 | 0.149 | 395 |
| DP119-3-E-M3 | 27,679 | 346 | 0.9973 | 2.76 | 0.1974 | 402 |
| DP119-10-C-F1 | 27,679 | 386 | 0.9961 | 2.72 | 0.1801 | 489 |
| DP119-10-C-F2 | 27,679 | 496 | 0.9963 | 4.19 | 0.055 | 601 |
| DP119-10-C-F3 | 27,679 | 511 | 0.9962 | 4.1 | 0.0507 | 610 |
| DP119-10-C-M1 | 27,679 | 262 | 0.997 | 2.33 | 0.1945 | 332 |
| DP119-10-C-M2 | 27,679 | 411 | 0.9956 | 2.7 | 0.1854 | 541 |
| DP119-10-C-M3 | 27,679 | 395 | 0.9963 | 3.04 | 0.1389 | 481 |
| DP119-10-E-F1 | 27,679 | 358 | 0.9978 | 4.12 | 0.0311 | 412 |
| DP119-10-E-F2 | 27,679 | 207 | 0.9987 | 3.26 | 0.0769 | 246 |
| DP119-10-E-F3 | 27,679 | 312 | 0.9982 | 4.13 | 0.0317 | 365 |
| DP119-10-E-M1 | 27,679 | 435 | 0.9971 | 4.53 | 0.0244 | 528 |
| DP119-10-E-M2 | 27,679 | 383 | 0.997 | 3.64 | 0.0764 | 446 |
| DP119-10-E-M3 | 27,679 | 451 | 0.997 | 4.48 | 0.0297 | 541 |
| DP120-3-C-F1 | 27,679 | 383 | 0.9982 | 4.66 | 0.0169 | 423 |
| DP120-3-C-F2 | 27,679 | 421 | 0.9976 | 4.77 | 0.0148 | 488 |
| DP120-3-C-F3 | 27,679 | 375 | 0.9975 | 3.88 | 0.0532 | 427 |
| DP120-3-C-M1 | 27,679 | 449 | 0.9971 | 4.65 | 0.0211 | 532 |
| DP120-3-C-M2 | 27,679 | 433 | 0.9967 | 4.53 | 0.0237 | 551 |
| DP120-3-C-M3 | 27,679 | 277 | 0.9979 | 3.23 | 0.0948 | 336 |
| DP120-3-E-F1 | 27,679 | 434 | 0.9974 | 4.45 | 0.0332 | 516 |
| DP120-3-E-F2 | 27,679 | 316 | 0.9974 | 2.43 | 0.3422 | 396 |
| DP120-3-E-F3 | 27,679 | 371 | 0.9967 | 3.82 | 0.0451 | 487 |
| DP120-3-E-M1 | 27,679 | 338 | 0.9963 | 1.96 | 0.4687 | 448 |
| DP120-3-E-M2 | 27,679 | 317 | 0.9966 | 2.99 | 0.1494 | 431 |
| DP120-3-E-M3 | 27,679 | 310 | 0.9968 | 3.59 | 0.0583 | 452 |
| DP120-10-C-F1 | 27,679 | 362 | 0.9969 | 3.36 | 0.101 | 447 |
| DP120-10-C-F2 | 27,679 | 382 | 0.9972 | 3.71 | 0.0717 | 446 |
| DP120-10-C-F3 | 27,679 | 361 | 0.9975 | 4.16 | 0.0325 | 426 |
| DP120-10-C-M1 | 27,679 | 313 | 0.9979 | 3.94 | 0.0471 | 385 |
| DP120-10-C-M2 | 27,679 | 318 | 0.9979 | 4.07 | 0.0334 | 362 |
| DP120-10-C-M3 | 27,679 | 398 | 0.9971 | 3.89 | 0.0507 | 470 |
| DP120-10-E-F1 | 27,679 | 385 | 0.9974 | 3.83 | 0.0576 | 451 |
| DP120-10-E-F2 | 27,679 | 342 | 0.9966 | 2.78 | 0.1674 | 454 |
| DP120-10-E-F3 | 27,679 | 353 | 0.9969 | 3.29 | 0.1083 | 425 |
| DP120-10-E-M1 | 27,679 | 391 | 0.9979 | 4.02 | 0.0415 | 424 |
| DP120-10-E-M2 | 27,679 | 327 | 0.9967 | 3 | 0.1362 | 412 |
| DP120-10-E-M3 | 27,679 | 318 | 0.9969 | 2.9 | 0.1475 | 397 |
| DP121-3-C-F1 | 27,679 | 344 | 0.9975 | 3.21 | 0.111 | 423 |
| DP121-3-C-F2 | 27,679 | 325 | 0.9979 | 3.3 | 0.0824 | 369 |
| DP121-3-C-F3 | 27,679 | 342 | 0.9974 | 3.1 | 0.1213 | 420 |
| DP121-3-C-M1 | 27,679 | 405 | 0.9963 | 3.56 | 0.0679 | 551 |
| DP121-3-C-M2 | 27,679 | 352 | 0.9973 | 3.27 | 0.1169 | 418 |
| DP121-3-C-M3 | 27,679 | 335 | 0.9973 | 3 | 0.1472 | 422 |
| DP121-3-E-F1 | 27,679 | 250 | 0.9974 | 2.6 | 0.1528 | 344 |
| DP121-3-E-F2 | 27,679 | 289 | 0.9972 | 2.54 | 0.1658 | 366 |
| DP121-3-E-F3 | 27,679 | 241 | 0.9977 | 2.62 | 0.1438 | 322 |
| DP121-3-E-M1 | 27,679 | 265 | 0.9967 | 2.32 | 0.2625 | 389 |
| DP121-3-E-M2 | 27,679 | 339 | 0.9971 | 3.31 | 0.0837 | 405 |
| DP121-3-E-M3 | 27,679 | 257 | 0.9972 | 2.44 | 0.1746 | 328 |
| DP121-10-C-F1 | 27,679 | 349 | 0.9974 | 3.93 | 0.0406 | 424 |
| DP121-10-C-F2 | 27,679 | 362 | 0.9977 | 4.11 | 0.033 | 421 |
| DP121-10-C-F3 | 27,679 | 368 | 0.9977 | 4.34 | 0.0287 | 440 |
| DP121-10-C-M1 | 27,679 | 423 | 0.9966 | 3.79 | 0.0572 | 501 |
| DP121-10-C-M2 | 27,679 | 374 | 0.9973 | 3.71 | 0.0799 | 458 |
| DP121-10-C-M3 | 27,679 | 451 | 0.9966 | 4.14 | 0.0466 | 533 |
| DP121-10-E-F1 | 27,679 | 392 | 0.9976 | 4.23 | 0.037 | 453 |
| DP121-10-E-F2 | 27,679 | 186 | 0.9985 | 3.12 | 0.078 | 223 |
| DP121-10-E-F3 | 27,679 | 357 | 0.9969 | 3.49 | 0.0723 | 444 |
| DP121-10-E-M1 | 27,679 | 431 | 0.9964 | 3.76 | 0.0675 | 539 |
| DP121-10-E-M2 | 27,679 | 369 | 0.9971 | 3.64 | 0.0671 | 448 |
| DP121-10-E-M3 | 27,679 | 383 | 0.9977 | 4.06 | 0.0482 | 446 |
| DP123-3-C-F1 | 27,679 | 448 | 0.9969 | 4.12 | 0.0469 | 528 |
| DP123-3-C-F2 | 27,679 | 510 | 0.997 | 4.47 | 0.0307 | 564 |
| DP123-3-C-F3 | 27,679 | 433 | 0.9964 | 3.29 | 0.1184 | 516 |
| DP123-3-C-M1 | 27,679 | 414 | 0.9972 | 2.91 | 0.2169 | 462 |
| DP123-3-C-M2 | 27,679 | 530 | 0.9967 | 4.21 | 0.0499 | 593 |
| DP123-3-C-M3 | 27,679 | 449 | 0.9954 | 3.05 | 0.1742 | 568 |
| DP123-3-E-F1 | 27,679 | 267 | 0.9975 | 2.57 | 0.217 | 338 |
| DP123-3-E-F2 | 27,679 | 339 | 0.9978 | 3.25 | 0.1461 | 381 |
| DP123-3-E-F3 | 27,679 | 199 | 0.9979 | 2 | 0.2411 | 252 |
| DP123-3-E-M1 | 27,679 | 222 | 0.9977 | 2.86 | 0.111 | 303 |
| DP123-3-E-M2 | 27,679 | 244 | 0.9974 | 2.87 | 0.1137 | 322 |
| DP123-3-E-M3 | 27,679 | 268 | 0.9976 | 2.75 | 0.1699 | 354 |
| DP123-10-C-F1 | 27,679 | 428 | 0.9971 | 4.04 | 0.0508 | 492 |
| DP123-10-C-F2 | 27,679 | 467 | 0.9968 | 4.2 | 0.0367 | 534 |
| DP123-10-C-F3 | 27,679 | 418 | 0.9962 | 3.12 | 0.133 | 511 |
| DP123-10-C-M1 | 27,679 | 310 | 0.9965 | 2.35 | 0.2907 | 433 |
| DP123-10-C-M2 | 27,679 | 490 | 0.9965 | 3.97 | 0.054 | 558 |
| DP123-10-C-M3 | 27,679 | 403 | 0.9957 | 2.64 | 0.248 | 533 |
| DP123-10-E-F1 | 27,679 | 237 | 0.9977 | 2.09 | 0.2691 | 287 |
| DP123-10-E-F2 | 27,679 | 169 | 0.9983 | 1.94 | 0.2793 | 227 |
| DP123-10-E-F3 | 27,679 | 175 | 0.9978 | 1.86 | 0.3024 | 273 |
| DP123-10-E-M1 | 27,679 | 327 | 0.9969 | 2.94 | 0.1701 | 405 |
| DP123-10-E-M2 | 27,679 | 274 | 0.997 | 3.03 | 0.1124 | 359 |
| DP123-10-E-M3 | 27,679 | 353 | 0.9967 | 3.26 | 0.115 | 446 |
| DP124-3-C-F1 | 27,679 | 313 | 0.9972 | 3.89 | 0.0374 | 404 |
| DP124-3-C-F2 | 27,679 | 398 | 0.9977 | 3.91 | 0.0625 | 443 |
| DP124-3-C-F3 | 27,679 | 333 | 0.9977 | 3.85 | 0.0502 | 392 |
| DP124-3-C-M1 | 27,679 | 408 | 0.998 | 4.7 | 0.0185 | 442 |
| DP124-3-C-M2 | 27,679 | 398 | 0.9973 | 4.17 | 0.0544 | 505 |
| DP124-3-C-M3 | 27,679 | 399 | 0.9978 | 4.17 | 0.0376 | 458 |
| DP124-3-E-F1 | 27,679 | 316 | 0.9976 | 3.49 | 0.0752 | 370 |
| DP124-3-E-F2 | 27,679 | 371 | 0.9968 | 3.91 | 0.0416 | 469 |
| DP124-3-E-F3 | 27,679 | 299 | 0.9975 | 3.62 | 0.0584 | 386 |
| DP124-3-E-M1 | 27,679 | 418 | 0.9981 | 4.19 | 0.0478 | 454 |
| DP124-3-E-M2 | 27,679 | 381 | 0.9971 | 3.38 | 0.132 | 453 |
| DP124-3-E-M3 | 27,679 | 450 | 0.9967 | 3.7 | 0.0869 | 541 |
| DP124-10-C-F1 | 27,679 | 401 | 0.9969 | 4.1 | 0.0365 | 470 |
| DP124-10-C-F2 | 27,679 | 315 | 0.9973 | 4.07 | 0.0334 | 407 |
| DP124-10-C-F3 | 27,679 | 297 | 0.998 | 3.86 | 0.0563 | 368 |
| DP124-10-C-M1 | 27,679 | 429 | 0.9978 | 4.81 | 0.0147 | 488 |
| DP124-10-C-M2 | 27,679 | 451 | 0.9974 | 4.59 | 0.0243 | 526 |
| DP124-10-C-M3 | 27,679 | 325 | 0.9978 | 3.93 | 0.0478 | 384 |
| DP124-10-E-F1 | 27,679 | 337 | 0.9966 | 3.06 | 0.1081 | 434 |
| DP124-10-E-F2 | 27,679 | 313 | 0.9979 | 3.22 | 0.1331 | 352 |
| DP124-10-E-F3 | 27,679 | 213 | 0.9984 | 2.68 | 0.2063 | 254 |
| DP124-10-E-M1 | 27,679 | 412 | 0.9973 | 4.46 | 0.0233 | 491 |
| DP124-10-E-M3 | 27,679 | 281 | 0.9977 | 3.78 | 0.053 | 337 |
| DP125-3-C-F1 | 27,679 | 321 | 0.9978 | 3.11 | 0.1408 | 371 |
| DP125-3-C-F2 | 27,679 | 152 | 0.9988 | 2.15 | 0.2303 | 178 |
| DP125-3-C-F3 | 27,679 | 258 | 0.9982 | 3.18 | 0.1257 | 303 |
| DP125-3-C-M1 | 27,679 | 256 | 0.9968 | 2.8 | 0.1424 | 384 |
| DP125-3-C-M2 | 27,679 | 312 | 0.9966 | 3.31 | 0.0827 | 396 |
| DP125-3-C-M3 | 27,679 | 250 | 0.9975 | 1.83 | 0.3482 | 310 |
| DP125-3-E-F1 | 27,679 | 276 | 0.9984 | 3.76 | 0.0606 | 317 |
| DP125-3-E-F2 | 27,679 | 362 | 0.9974 | 3.05 | 0.1562 | 415 |
| DP125-3-E-F3 | 27,679 | 277 | 0.9968 | 2.83 | 0.1286 | 417 |
| DP125-3-E-M1 | 27,679 | 324 | 0.9971 | 3.12 | 0.1372 | 403 |
| DP125-3-E-M2 | 27,679 | 245 | 0.9975 | 2.47 | 0.185 | 310 |
| DP125-3-E-M3 | 27,679 | 283 | 0.9976 | 2.59 | 0.2191 | 341 |
| DP125-10-C-F1 | 27,679 | 363 | 0.9973 | 3.8 | 0.0641 | 438 |
| DP125-10-C-F2 | 27,679 | 254 | 0.9979 | 3.43 | 0.0822 | 323 |
| DP125-10-C-F3 | 27,679 | 220 | 0.9985 | 3.61 | 0.0592 | 271 |
| DP125-10-C-M1 | 27,679 | 343 | 0.9969 | 3.56 | 0.0753 | 424 |
| DP125-10-C-M2 | 27,679 | 374 | 0.9969 | 3.81 | 0.0651 | 454 |
| DP125-10-C-M3 | 27,679 | 301 | 0.9982 | 3.82 | 0.0513 | 342 |
| DP125-10-E-F1 | 27,679 | 229 | 0.9975 | 2.16 | 0.2251 | 298 |
| DP125-10-E-F2 | 27,679 | 234 | 0.9977 | 2.55 | 0.1641 | 286 |
| DP125-10-E-F3 | 27,679 | 345 | 0.9974 | 3.22 | 0.1401 | 406 |
| DP125-10-E-M1 | 27,679 | 389 | 0.9972 | 3.53 | 0.1115 | 462 |
| DP125-10-E-M2 | 27,679 | 351 | 0.9967 | 3.24 | 0.1181 | 429 |
| DP125-10-E-M3 | 27,679 | 344 | 0.9971 | 3.42 | 0.0967 | 406 |
| DP126-3-C-F1 | 27,679 | 396 | 0.9965 | 3.87 | 0.0652 | 530 |
| DP126-3-C-F2 | 27,679 | 450 | 0.9957 | 3.32 | 0.105 | 605 |
| DP126-3-C-F3 | 27,679 | 395 | 0.9965 | 3.27 | 0.097 | 490 |
| DP126-3-C-M1 | 27,679 | 371 | 0.9966 | 2.47 | 0.2352 | 445 |
| DP126-3-C-M2 | 27,679 | 469 | 0.9956 | 3.16 | 0.1564 | 579 |
| DP126-3-C-M3 | 27,679 | 435 | 0.996 | 3.18 | 0.1091 | 537 |
| DP126-3-E-F1 | 27,679 | 462 | 0.9977 | 4.7 | 0.0202 | 503 |
| DP126-3-E-F2 | 27,679 | 449 | 0.9965 | 4.49 | 0.024 | 574 |
| DP126-3-E-F3 | 27,679 | 431 | 0.9975 | 4.53 | 0.023 | 486 |
| DP126-3-E-M1 | 27,679 | 511 | 0.996 | 4.46 | 0.0414 | 638 |
| DP126-3-E-M2 | 27,679 | 474 | 0.9968 | 4.41 | 0.0361 | 574 |
| DP126-3-E-M3 | 27,679 | 484 | 0.9974 | 4.68 | 0.0197 | 544 |
| DP126-10-C-F1 | 27,679 | 357 | 0.9971 | 3.53 | 0.0894 | 445 |
| DP126-10-C-F2 | 27,679 | 426 | 0.9963 | 3.25 | 0.1116 | 509 |
| DP126-10-C-F3 | 27,679 | 263 | 0.9967 | 2.19 | 0.2684 | 352 |
| DP126-10-C-M1 | 27,679 | 337 | 0.9959 | 2.11 | 0.261 | 452 |
| DP126-10-C-M2 | 27,679 | 352 | 0.9962 | 2.52 | 0.232 | 450 |
| DP126-10-C-M3 | 27,679 | 454 | 0.9962 | 3.63 | 0.0718 | 559 |
| DP126-10-E-F1 | 27,679 | 244 | 0.9975 | 2.95 | 0.1011 | 322 |
| DP126-10-E-F2 | 27,679 | 406 | 0.9977 | 4.19 | 0.0355 | 458 |
| DP126-10-E-F3 | 27,679 | 427 | 0.9963 | 3.96 | 0.0424 | 532 |
| DP126-10-E-M1 | 27,679 | 462 | 0.9968 | 4.4 | 0.0305 | 542 |
| DP126-10-E-M2 | 27,679 | 356 | 0.9967 | 3.6 | 0.064 | 440 |
| DP126-10-E-M3 | 27,679 | 485 | 0.9975 | 4.82 | 0.0158 | 543 |
| DP127-3-C-F1 | 27,679 | 413 | 0.9969 | 4.01 | 0.0499 | 500 |
| DP127-3-C-F2 | 27,679 | 460 | 0.9967 | 3.67 | 0.0632 | 526 |
| DP127-3-C-F3 | 27,679 | 512 | 0.9964 | 4.64 | 0.024 | 650 |
| DP127-3-C-M1 | 27,679 | 446 | 0.9964 | 3.19 | 0.133 | 517 |
| DP127-3-C-M2 | 27,679 | 435 | 0.9961 | 3.33 | 0.1022 | 534 |
| DP127-3-C-M3 | 27,679 | 419 | 0.9959 | 3.05 | 0.1256 | 526 |
| DP127-3-E-F1 | 27,679 | 293 | 0.9973 | 2.53 | 0.2029 | 352 |
| DP127-3-E-F2 | 27,679 | 313 | 0.9975 | 3 | 0.1136 | 370 |
| DP127-3-E-F3 | 27,679 | 291 | 0.997 | 2.8 | 0.1361 | 376 |
| DP127-3-E-M1 | 27,679 | 146 | 0.9987 | 2.29 | 0.1963 | 183 |
| DP127-3-E-M2 | 27,679 | 294 | 0.997 | 2.93 | 0.1111 | 381 |
| DP127-3-E-M3 | 27,679 | 189 | 0.9971 | 2.57 | 0.1588 | 343 |
| DP127-10-C-F1 | 27,679 | 411 | 0.9968 | 4.03 | 0.0389 | 479 |
| DP127-10-C-F2 | 27,679 | 417 | 0.996 | 3.06 | 0.1107 | 520 |
| DP127-10-C-F3 | 27,679 | 498 | 0.9967 | 4.58 | 0.0236 | 593 |
| DP127-10-C-M1 | 27,679 | 459 | 0.9956 | 2.91 | 0.1625 | 591 |
| DP127-10-C-M2 | 27,679 | 472 | 0.9962 | 3.35 | 0.107 | 553 |
| DP127-10-C-M3 | 27,679 | 415 | 0.9957 | 2.9 | 0.1463 | 530 |
| DP127-10-E-F1 | 27,679 | 352 | 0.9982 | 4.39 | 0.0265 | 401 |
| DP127-10-E-F2 | 27,679 | 357 | 0.9971 | 3.96 | 0.0661 | 480 |
| DP127-10-E-F3 | 27,679 | 359 | 0.9983 | 4.36 | 0.0306 | 404 |
| DP127-10-E-M1 | 27,679 | 391 | 0.9973 | 4.31 | 0.0276 | 505 |
| DP127-10-E-M2 | 27,679 | 373 | 0.9974 | 4.41 | 0.0226 | 440 |
| DP127-10-E-M3 | 27,679 | 388 | 0.9971 | 4.36 | 0.0249 | 478 |
| DP128-3-C-F1 | 27,679 | 468 | 0.9964 | 3.61 | 0.0934 | 538 |
| DP128-3-C-F2 | 27,679 | 370 | 0.9966 | 2.68 | 0.2082 | 467 |
| DP128-3-C-F3 | 27,679 | 443 | 0.9964 | 3.75 | 0.0749 | 548 |
| DP128-3-C-M1 | 27,679 | 449 | 0.9967 | 3.22 | 0.127 | 512 |
| DP128-3-C-M2 | 27,679 | 385 | 0.9959 | 2.61 | 0.1984 | 512 |
| DP128-3-C-M3 | 27,679 | 415 | 0.9955 | 3.16 | 0.1327 | 549 |
| DP128-3-E-F1 | 27,679 | 300 | 0.997 | 3.55 | 0.061 | 397 |
| DP128-3-E-F2 | 27,679 | 440 | 0.9969 | 4.25 | 0.0342 | 516 |
| DP128-3-E-F3 | 27,679 | 306 | 0.9968 | 3.39 | 0.0984 | 418 |
| DP128-3-E-M1 | 27,679 | 177 | 0.9978 | 2.59 | 0.1495 | 247 |
| DP128-3-E-M2 | 27,679 | 272 | 0.9966 | 2.88 | 0.1118 | 393 |
| DP128-3-E-M3 | 27,679 | 192 | 0.9974 | 2.65 | 0.1446 | 327 |
| DP128-10-C-F1 | 27,679 | 344 | 0.9959 | 2.9 | 0.1555 | 491 |
| DP128-10-C-F2 | 27,679 | 515 | 0.9963 | 3.71 | 0.1115 | 592 |
| DP128-10-C-F3 | 27,679 | 511 | 0.9959 | 4.06 | 0.0579 | 645 |
| DP128-10-C-M1 | 27,679 | 482 | 0.9952 | 3.27 | 0.1221 | 611 |
| DP128-10-C-M2 | 27,679 | 388 | 0.9961 | 2.6 | 0.2027 | 514 |
| DP128-10-C-M3 | 27,679 | 529 | 0.9964 | 4 | 0.0627 | 612 |
| DP128-10-E-F1 | 27,679 | 382 | 0.9962 | 3.91 | 0.0382 | 508 |
| DP128-10-E-F2 | 27,679 | 367 | 0.9974 | 3.89 | 0.0508 | 430 |
| DP128-10-E-F3 | 27,679 | 331 | 0.9967 | 2.96 | 0.1764 | 407 |
| DP128-10-E-M1 | 27,679 | 246 | 0.9975 | 2.7 | 0.1327 | 315 |
| DP128-10-E-M2 | 27,679 | 188 | 0.9976 | 1.92 | 0.3693 | 286 |
| DP128-10-E-M3 | 27,679 | 254 | 0.9978 | 3.22 | 0.0832 | 317 |
| DP129-3-C-F1 | 27,679 | 525 | 0.9961 | 3.93 | 0.0935 | 634 |
| DP129-3-C-F2 | 27,679 | 379 | 0.9965 | 3.54 | 0.0848 | 482 |
| DP129-3-C-F3 | 27,679 | 477 | 0.9961 | 3.37 | 0.1384 | 573 |
| DP129-3-C-M1 | 27,679 | 407 | 0.9963 | 2.77 | 0.1558 | 482 |
| DP129-3-C-M2 | 27,679 | 377 | 0.9959 | 3.01 | 0.1221 | 492 |
| DP129-3-C-M3 | 27,679 | 455 | 0.996 | 3.43 | 0.1086 | 568 |
| DP129-3-E-F1 | 27,679 | 477 | 0.9972 | 4.6 | 0.0203 | 532 |
| DP129-3-E-F2 | 27,679 | 484 | 0.996 | 3.83 | 0.0685 | 601 |
| DP129-3-E-F3 | 27,679 | 384 | 0.996 | 3.54 | 0.0652 | 506 |
| DP129-3-E-M1 | 27,679 | 248 | 0.9972 | 2.74 | 0.1222 | 352 |
| DP129-3-E-M2 | 27,679 | 371 | 0.9969 | 3.37 | 0.0858 | 447 |
| DP129-3-E-M3 | 27,679 | 303 | 0.9967 | 2.83 | 0.132 | 403 |
| DP129-10-C-F1 | 27,679 | 523 | 0.9965 | 3.76 | 0.1204 | 605 |
| DP129-10-C-F2 | 27,679 | 378 | 0.9966 | 3.43 | 0.1024 | 487 |
| DP129-10-C-F3 | 27,679 | 437 | 0.9964 | 3.03 | 0.1747 | 515 |
| DP129-10-C-M1 | 27,679 | 434 | 0.9967 | 2.85 | 0.179 | 493 |
| DP129-10-C-M2 | 27,679 | 405 | 0.9962 | 3.26 | 0.0995 | 501 |
| DP129-10-C-M3 | 27,679 | 305 | 0.9967 | 2.61 | 0.1529 | 426 |
| DP129-10-E-F1 | 27,679 | 430 | 0.9965 | 3.67 | 0.0846 | 531 |
| DP129-10-E-F2 | 27,679 | 272 | 0.9974 | 2.75 | 0.1248 | 341 |
| DP129-10-E-F3 | 27,679 | 339 | 0.9965 | 3.05 | 0.1014 | 453 |
| DP129-10-E-M1 | 27,679 | 375 | 0.9963 | 3.44 | 0.0705 | 476 |
| DP129-10-E-M2 | 27,679 | 312 | 0.9972 | 3.12 | 0.092 | 377 |
| DP129-10-E-M3 | 27,679 | 399 | 0.9958 | 3.47 | 0.0722 | 512 |
| DP130-3-C-F1 | 27,679 | 462 | 0.9961 | 3.65 | 0.0843 | 573 |
| DP130-3-C-F2 | 27,679 | 499 | 0.9961 | 4.15 | 0.047 | 608 |
| DP130-3-C-F3 | 27,679 | 484 | 0.9974 | 4.08 | 0.0541 | 528 |
| DP130-3-C-M1 | 27,679 | 381 | 0.9965 | 2.78 | 0.1726 | 484 |
| DP130-3-C-M2 | 27,679 | 371 | 0.996 | 2.96 | 0.1238 | 482 |
| DP130-3-C-M3 | 27,679 | 393 | 0.9958 | 3.02 | 0.1479 | 517 |
| DP130-10-C-F1 | 27,679 | 354 | 0.9964 | 3.28 | 0.1069 | 464 |
| DP130-10-C-F2 | 27,679 | 425 | 0.9962 | 3.67 | 0.0633 | 526 |
| DP130-10-C-F3 | 27,679 | 428 | 0.9971 | 3.7 | 0.076 | 488 |
| DP130-10-C-M1 | 27,679 | 298 | 0.9973 | 3.07 | 0.1098 | 371 |
| DP130-10-C-M2 | 27,679 | 340 | 0.997 | 3.23 | 0.0859 | 405 |
| DP130-10-C-M3 | 27,679 | 437 | 0.996 | 3.14 | 0.149 | 542 |
| DP132-3-C-F1 | 27,679 | 486 | 0.9969 | 3.94 | 0.0633 | 543 |
| DP132-3-C-F2 | 27,679 | 402 | 0.997 | 2.93 | 0.1872 | 462 |
| DP132-3-C-F3 | 27,679 | 353 | 0.9974 | 3.47 | 0.1091 | 435 |
| DP132-3-C-M1 | 27,679 | 445 | 0.9959 | 2.9 | 0.1532 | 549 |
| DP132-3-C-M2 | 27,679 | 280 | 0.9972 | 2.36 | 0.2173 | 350 |
| DP132-3-C-M3 | 27,679 | 412 | 0.995 | 2.79 | 0.16 | 609 |
| DP132-3-E-F1 | 27,679 | 306 | 0.9978 | 3.76 | 0.0443 | 367 |
| DP132-3-E-F2 | 27,679 | 346 | 0.997 | 3.53 | 0.0743 | 463 |
| DP132-3-E-F3 | 27,679 | 314 | 0.9982 | 3.6 | 0.0626 | 352 |
| DP132-3-E-M1 | 27,679 | 322 | 0.9974 | 3.39 | 0.0769 | 405 |
| DP132-3-E-M2 | 27,679 | 252 | 0.9969 | 3.03 | 0.0842 | 360 |
| DP132-3-E-M3 | 27,679 | 312 | 0.9973 | 3.37 | 0.0729 | 410 |
| DP132-10-C-F1 | 27,679 | 439 | 0.9965 | 3.61 | 0.0828 | 534 |
| DP132-10-C-F2 | 27,679 | 521 | 0.997 | 3.92 | 0.0949 | 592 |
| DP132-10-C-F3 | 27,679 | 339 | 0.9967 | 2.81 | 0.201 | 432 |
| DP132-10-C-M1 | 27,679 | 405 | 0.9958 | 2.62 | 0.1674 | 506 |
| DP132-10-C-M2 | 27,679 | 266 | 0.9973 | 2.4 | 0.212 | 332 |
| DP132-10-C-M3 | 27,679 | 496 | 0.9966 | 3.77 | 0.0776 | 582 |
| DP132-10-E-F1 | 27,679 | 328 | 0.9975 | 3.37 | 0.1114 | 380 |
| DP132-10-E-F2 | 27,679 | 287 | 0.998 | 3.46 | 0.071 | 359 |
| DP132-10-E-F3 | 27,679 | 311 | 0.998 | 3.42 | 0.0935 | 364 |
| DP132-10-E-M1 | 27,679 | 339 | 0.9982 | 4.03 | 0.0486 | 381 |
| DP132-10-E-M2 | 27,679 | 305 | 0.9978 | 3.35 | 0.1114 | 381 |
| DP132-10-E-M3 | 27,679 | 338 | 0.9975 | 3.48 | 0.1221 | 413 |
| DP133-3-C-F1 | 27,679 | 441 | 0.9974 | 4.08 | 0.0436 | 486 |
| DP133-3-C-F2 | 27,679 | 329 | 0.997 | 3.06 | 0.1211 | 399 |
| DP133-3-C-F3 | 27,679 | 453 | 0.9974 | 3.87 | 0.0573 | 499 |
| DP133-3-C-M1 | 27,679 | 363 | 0.9965 | 2.97 | 0.1268 | 458 |
| DP133-3-C-M2 | 27,679 | 288 | 0.996 | 2.37 | 0.2055 | 437 |
| DP133-3-C-M3 | 27,679 | 523 | 0.9967 | 3.97 | 0.081 | 599 |
| DP133-3-E-F1 | 27,679 | 386 | 0.9978 | 4.43 | 0.0251 | 431 |
| DP133-3-E-F2 | 27,679 | 287 | 0.9969 | 3.29 | 0.0886 | 391 |
| DP133-3-E-F3 | 27,679 | 366 | 0.9961 | 3.28 | 0.1024 | 461 |
| DP133-3-E-M1 | 27,679 | 378 | 0.9976 | 4.23 | 0.0336 | 438 |
| DP133-3-E-M2 | 27,679 | 376 | 0.9975 | 4.01 | 0.0422 | 428 |
| DP133-3-E-M3 | 27,679 | 249 | 0.9973 | 1.76 | 0.4804 | 333 |
| DP133-10-C-F1 | 27,679 | 418 | 0.9965 | 3.69 | 0.0636 | 511 |
| DP133-10-C-F2 | 27,679 | 339 | 0.9962 | 2.56 | 0.1884 | 440 |
| DP133-10-C-F3 | 27,679 | 446 | 0.9961 | 3.62 | 0.0698 | 584 |
| DP133-10-C-M1 | 27,679 | 317 | 0.9967 | 2.36 | 0.2003 | 394 |
| DP133-10-C-M2 | 27,679 | 244 | 0.9969 | 2.25 | 0.2236 | 394 |
| DP133-10-C-M3 | 27,679 | 508 | 0.9968 | 4.03 | 0.0716 | 585 |
| DP133-10-E-F1 | 27,679 | 434 | 0.9961 | 3.77 | 0.0764 | 522 |
| DP133-10-E-F2 | 27,679 | 423 | 0.9975 | 4.56 | 0.0222 | 501 |
| DP133-10-E-F3 | 27,679 | 311 | 0.9971 | 3.42 | 0.0686 | 396 |
| DP133-10-E-M1 | 27,679 | 437 | 0.9963 | 4.31 | 0.0301 | 547 |
| DP133-10-E-M2 | 27,679 | 395 | 0.9978 | 4.29 | 0.0306 | 454 |
| DP133-10-E-M3 | 27,679 | 436 | 0.9971 | 4.36 | 0.0401 | 503 |
| DP134-3-C-F1 | 27,679 | 434 | 0.9965 | 3.54 | 0.0983 | 523 |
| DP134-3-C-F2 | 27,679 | 385 | 0.9969 | 3.09 | 0.1378 | 466 |
| DP134-3-C-F3 | 27,679 | 371 | 0.996 | 3 | 0.1151 | 457 |
| DP134-3-C-M1 | 27,679 | 451 | 0.9958 | 3.16 | 0.1307 | 566 |
| DP134-3-C-M2 | 27,679 | 457 | 0.9962 | 3.16 | 0.1343 | 555 |
| DP134-3-C-M3 | 27,679 | 420 | 0.9963 | 3.04 | 0.127 | 506 |
| DP134-3-E-F1 | 27,679 | 582 | 0.9966 | 4.74 | 0.0206 | 681 |
| DP134-3-E-F2 | 27,679 | 489 | 0.9966 | 4.64 | 0.0216 | 580 |
| DP134-3-E-F3 | 27,679 | 583 | 0.9965 | 4.77 | 0.0198 | 663 |
| DP134-3-E-M1 | 27,679 | 384 | 0.9971 | 3.92 | 0.0976 | 512 |
| DP134-3-E-M2 | 27,679 | 405 | 0.9976 | 4.27 | 0.0518 | 459 |
| DP134-3-E-M3 | 27,679 | 384 | 0.9979 | 3.95 | 0.0855 | 432 |
| DP134-10-C-F1 | 27,679 | 426 | 0.997 | 3.66 | 0.0802 | 493 |
| DP134-10-C-F2 | 27,679 | 201 | 0.9978 | 2.18 | 0.248 | 269 |
| DP134-10-C-F3 | 27,679 | 331 | 0.9961 | 2.92 | 0.1172 | 444 |
| DP134-10-C-M1 | 27,679 | 417 | 0.9961 | 2.86 | 0.1579 | 509 |
| DP134-10-C-M2 | 27,679 | 437 | 0.9963 | 3.07 | 0.1503 | 593 |
| DP134-10-C-M3 | 27,679 | 440 | 0.9964 | 3.08 | 0.141 | 537 |
| DP134-10-E-F1 | 27,679 | 439 | 0.996 | 4.01 | 0.0439 | 544 |
| DP134-10-E-F2 | 27,679 | 298 | 0.9977 | 3.6 | 0.0736 | 339 |
| DP134-10-E-F3 | 27,679 | 408 | 0.9959 | 3.85 | 0.0465 | 555 |
| DP134-10-E-M1 | 27,679 | 436 | 0.9974 | 4.16 | 0.0496 | 489 |
| DP134-10-E-M2 | 27,679 | 410 | 0.9967 | 4.09 | 0.0429 | 524 |
| DP134-10-E-M3 | 27,679 | 421 | 0.9966 | 4.08 | 0.0366 | 514 |
| DP135-3-C-F1 | 27,679 | 326 | 0.9968 | 3.1 | 0.1089 | 419 |
| DP135-3-C-F2 | 27,679 | 464 | 0.9965 | 3.6 | 0.0971 | 557 |
| DP135-3-C-F3 | 27,679 | 464 | 0.996 | 3.25 | 0.1715 | 579 |
| DP135-3-C-M1 | 27,679 | 405 | 0.9964 | 3.05 | 0.134 | 493 |
| DP135-3-C-M2 | 27,679 | 409 | 0.9964 | 2.77 | 0.1909 | 506 |
| DP135-3-C-M3 | 27,679 | 388 | 0.9962 | 3.26 | 0.1065 | 502 |
| DP135-3-E-F1 | 27,679 | 424 | 0.9975 | 4.5 | 0.0213 | 511 |
| DP135-3-E-F2 | 27,679 | 432 | 0.9963 | 3.9 | 0.0508 | 518 |
| DP135-3-E-F3 | 27,679 | 445 | 0.9967 | 3.99 | 0.0436 | 524 |
| DP135-3-E-M1 | 27,679 | 403 | 0.9969 | 3.68 | 0.0774 | 472 |
| DP135-3-E-M2 | 27,679 | 406 | 0.9971 | 3.5 | 0.0973 | 494 |
| DP135-3-E-M3 | 27,679 | 380 | 0.9977 | 4.05 | 0.0448 | 430 |
| DP135-10-C-F1 | 27,679 | 213 | 0.9973 | 2.36 | 0.196 | 306 |
| DP135-10-C-F2 | 27,679 | 429 | 0.996 | 2.94 | 0.1906 | 531 |
| DP135-10-C-F3 | 27,679 | 476 | 0.996 | 3.27 | 0.1652 | 594 |
| DP135-10-C-M1 | 27,679 | 398 | 0.9959 | 2.91 | 0.1468 | 515 |
| DP135-10-C-M2 | 27,679 | 331 | 0.9964 | 2.04 | 0.2786 | 423 |
| DP135-10-C-M3 | 27,679 | 394 | 0.9965 | 3.29 | 0.1077 | 475 |
| DP135-10-E-F1 | 27,679 | 474 | 0.9967 | 4.39 | 0.0273 | 561 |
| DP135-10-E-F2 | 27,679 | 389 | 0.9973 | 3.55 | 0.0975 | 448 |
| DP135-10-E-F3 | 27,679 | 361 | 0.9966 | 2.87 | 0.1968 | 448 |
| DP135-10-E-M1 | 27,679 | 426 | 0.996 | 3.5 | 0.1044 | 551 |
| DP135-10-E-M2 | 27,679 | 355 | 0.9967 | 3.35 | 0.0812 | 446 |
| DP135-10-E-M3 | 27,679 | 440 | 0.997 | 3.93 | 0.0534 | 510 |
| DP136-3-C-F1 | 27,679 | 499 | 0.9955 | 3.69 | 0.0741 | 626 |
| DP136-3-C-F2 | 27,679 | 564 | 0.9959 | 4.53 | 0.0295 | 675 |
| DP136-3-C-F3 | 27,679 | 501 | 0.9966 | 4.26 | 0.0466 | 592 |
| DP136-3-C-M1 | 27,679 | 455 | 0.9965 | 3.66 | 0.0795 | 530 |
| DP136-3-C-M2 | 27,679 | 475 | 0.9963 | 3.74 | 0.0566 | 557 |
| DP136-3-C-M3 | 27,679 | 404 | 0.9973 | 3.78 | 0.0617 | 466 |
| DP136-3-E-F1 | 27,679 | 415 | 0.9969 | 3.96 | 0.0453 | 498 |
| DP136-3-E-F2 | 27,679 | 413 | 0.996 | 3.45 | 0.0834 | 522 |
| DP136-3-E-F3 | 27,679 | 285 | 0.9972 | 3.26 | 0.0992 | 350 |
| DP136-3-E-M1 | 27,679 | 407 | 0.9965 | 3.81 | 0.0518 | 502 |
| DP136-3-E-M2 | 27,679 | 478 | 0.9956 | 3.69 | 0.0694 | 610 |
| DP136-3-E-M3 | 27,679 | 536 | 0.996 | 4.39 | 0.0311 | 639 |
| DP136-10-C-F1 | 27,679 | 469 | 0.996 | 3.79 | 0.0709 | 569 |
| DP136-10-C-F2 | 27,679 | 487 | 0.9961 | 4.04 | 0.0423 | 564 |
| DP136-10-C-F3 | 27,679 | 440 | 0.9956 | 3.67 | 0.0594 | 600 |
| DP136-10-C-M1 | 27,679 | 470 | 0.9965 | 3.77 | 0.0646 | 542 |
| DP136-10-C-M2 | 27,679 | 465 | 0.996 | 3.74 | 0.0861 | 554 |
| DP136-10-C-M3 | 27,679 | 442 | 0.9968 | 3.83 | 0.0743 | 513 |
| DP136-10-E-F1 | 27,679 | 418 | 0.9975 | 4.25 | 0.0378 | 496 |
| DP136-10-E-F2 | 27,679 | 378 | 0.9973 | 3.99 | 0.0469 | 457 |
| DP136-10-E-F3 | 27,679 | 524 | 0.9967 | 4.6 | 0.025 | 588 |
| DP136-10-E-M1 | 27,679 | 476 | 0.9971 | 4.72 | 0.0182 | 539 |
| DP136-10-E-M2 | 27,679 | 449 | 0.9965 | 3.8 | 0.0618 | 520 |
| DP136-10-E-M3 | 27,679 | 410 | 0.9963 | 3.95 | 0.0416 | 517 |
| DP137-3-C-F1 | 27,679 | 220 | 0.9983 | 2.61 | 0.1751 | 271 |
| DP137-3-C-F2 | 27,679 | 284 | 0.9973 | 2.72 | 0.132 | 350 |
| DP137-3-C-F3 | 27,679 | 232 | 0.9983 | 2.71 | 0.1599 | 281 |
| DP137-3-C-M1 | 27,679 | 391 | 0.9975 | 4.11 | 0.0376 | 451 |
| DP137-3-C-M2 | 27,679 | 412 | 0.9973 | 4.46 | 0.0229 | 493 |
| DP137-3-C-M3 | 27,679 | 302 | 0.9981 | 3.15 | 0.1209 | 348 |
| DP137-3-E-F1 | 27,679 | 547 | 0.9971 | 4.84 | 0.0209 | 613 |
| DP137-3-E-F2 | 27,679 | 342 | 0.9977 | 3.61 | 0.0912 | 398 |
| DP137-3-E-F3 | 27,679 | 374 | 0.9977 | 3.75 | 0.0743 | 454 |
| DP137-3-E-M1 | 27,679 | 359 | 0.997 | 3.37 | 0.083 | 442 |
| DP137-3-E-M2 | 27,679 | 316 | 0.9973 | 2.73 | 0.1556 | 368 |
| DP137-3-E-M3 | 27,679 | 335 | 0.9969 | 2.95 | 0.1127 | 401 |
| DP137-10-C-F1 | 27,679 | 202 | 0.9976 | 2.3 | 0.1882 | 274 |
| DP137-10-C-F2 | 27,679 | 201 | 0.998 | 2.55 | 0.185 | 261 |
| DP137-10-C-F3 | 27,679 | 281 | 0.9973 | 2.59 | 0.2095 | 365 |
| DP137-10-C-M1 | 27,679 | 385 | 0.9968 | 3.69 | 0.0742 | 470 |
| DP137-10-C-M2 | 27,679 | 411 | 0.9958 | 3.96 | 0.0506 | 563 |
| DP137-10-E-F1 | 27,679 | 405 | 0.9963 | 2.91 | 0.1761 | 527 |
| DP137-10-E-F2 | 27,679 | 335 | 0.9975 | 3.13 | 0.1474 | 400 |
| DP137-10-E-F3 | 27,679 | 358 | 0.9974 | 3.9 | 0.0656 | 469 |
| DP137-10-E-M1 | 27,679 | 347 | 0.997 | 3.53 | 0.0687 | 420 |
| DP137-10-E-M2 | 27,679 | 354 | 0.997 | 3.76 | 0.043 | 457 |
| DP137-10-E-M3 | 27,679 | 308 | 0.997 | 3.21 | 0.0876 | 382 |
| DP138-3-C-F1 | 27,679 | 405 | 0.9964 | 3.9 | 0.0499 | 522 |
| DP138-3-C-F2 | 27,679 | 484 | 0.9966 | 4.19 | 0.0397 | 565 |
| DP138-3-C-F3 | 27,679 | 481 | 0.997 | 4.46 | 0.0266 | 558 |
| DP138-3-C-F4 | 27,679 | 446 | 0.9969 | 4.18 | 0.0432 | 524 |
| DP138-3-C-F5 | 27,679 | 452 | 0.9968 | 4.11 | 0.0502 | 535 |
| DP138-3-E-F1 | 27,679 | 479 | 0.9958 | 3.86 | 0.0515 | 602 |
| DP138-3-E-F2 | 27,679 | 441 | 0.9966 | 4.26 | 0.028 | 525 |
| DP138-3-E-F3 | 27,679 | 421 | 0.9969 | 4.04 | 0.0376 | 496 |
| DP138-3-E-M1 | 27,679 | 375 | 0.9971 | 3.79 | 0.0443 | 428 |
| DP138-3-E-M2 | 27,679 | 500 | 0.9963 | 4.31 | 0.0323 | 584 |
| DP138-10-C-F1 | 27,679 | 480 | 0.9974 | 4.69 | 0.0194 | 527 |
| DP138-10-C-F2 | 27,679 | 410 | 0.9971 | 4.1 | 0.0456 | 489 |
| DP138-10-C-F3 | 27,679 | 520 | 0.9958 | 4.58 | 0.0249 | 651 |
| DP138-10-C-F4 | 27,679 | 472 | 0.9971 | 4.64 | 0.0197 | 533 |
| DP138-10-C-F5 | 27,679 | 454 | 0.9968 | 3.98 | 0.0779 | 529 |
| DP138-10-E-F1 | 27,679 | 472 | 0.9965 | 4.4 | 0.0246 | 550 |
| DP138-10-E-F2 | 27,679 | 482 | 0.9957 | 4.18 | 0.0324 | 599 |
| DP138-10-E-F3 | 27,679 | 501 | 0.9956 | 4.15 | 0.0405 | 643 |
| DP138-10-E-M1 | 27,679 | 489 | 0.9961 | 4.34 | 0.0291 | 615 |
| DP138-10-E-M2 | 27,679 | 450 | 0.9967 | 4.1 | 0.0451 | 539 |
| DP139-3-C-F1 | 27,679 | 497 | 0.9964 | 3.99 | 0.0574 | 600 |
| DP139-3-C-F2 | 27,679 | 445 | 0.9956 | 3.08 | 0.1642 | 585 |
| DP139-3-C-F3 | 27,679 | 410 | 0.9955 | 2.84 | 0.2002 | 531 |
| DP139-3-C-M1 | 27,679 | 363 | 0.9963 | 2.47 | 0.2333 | 468 |
| DP139-3-C-M2 | 27,679 | 392 | 0.9965 | 3.19 | 0.1181 | 473 |
| DP139-3-C-M3 | 27,679 | 434 | 0.996 | 3.28 | 0.1199 | 552 |
| DP139-10-C-F1 | 27,679 | 483 | 0.9969 | 3.74 | 0.0827 | 548 |
| DP139-10-C-F2 | 27,679 | 515 | 0.9966 | 3.77 | 0.0784 | 596 |
| DP139-10-C-F3 | 27,679 | 286 | 0.9971 | 2.34 | 0.2658 | 363 |
| DP139-10-C-M1 | 27,679 | 374 | 0.9966 | 2.5 | 0.2136 | 463 |
| DP139-10-C-M2 | 27,679 | 381 | 0.9966 | 2.99 | 0.1445 | 462 |
| DP139-10-C-M3 | 27,679 | 380 | 0.9962 | 2.57 | 0.1985 | 468 |
| DP140-3-C-F1 | 27,679 | 572 | 0.9956 | 4.75 | 0.017 | 687 |
| DP140-3-C-F2 | 27,679 | 481 | 0.9958 | 4.24 | 0.0375 | 590 |
| DP140-3-C-F3 | 27,679 | 561 | 0.9956 | 4.71 | 0.0173 | 686 |
| DP140-3-C-F4 | 27,679 | 512 | 0.9955 | 4.36 | 0.0314 | 643 |
| DP140-3-C-F5 | 27,679 | 593 | 0.9957 | 4.77 | 0.0182 | 684 |
| DP140-3-E-F1 | 27,679 | 327 | 0.9961 | 3.87 | 0.0436 | 474 |
| DP140-3-E-F2 | 27,679 | 481 | 0.9956 | 4.16 | 0.0395 | 606 |
| DP140-3-E-F3 | 27,679 | 432 | 0.9964 | 3.72 | 0.0733 | 525 |
| DP140-3-E-M1 | 27,679 | 427 | 0.9968 | 3.92 | 0.0557 | 502 |
| DP140-3-E-M2 | 27,679 | 595 | 0.9965 | 4.93 | 0.0219 | 674 |
| DP140-10-C-F1 | 27,679 | 534 | 0.9962 | 4.77 | 0.0161 | 632 |
| DP140-10-C-F2 | 27,679 | 518 | 0.9957 | 4.33 | 0.0341 | 635 |
| DP140-10-C-F3 | 27,679 | 456 | 0.997 | 4.76 | 0.0158 | 541 |
| DP140-10-C-F4 | 27,679 | 574 | 0.9966 | 4.76 | 0.0218 | 652 |
| DP140-10-C-F5 | 27,679 | 467 | 0.9951 | 3.77 | 0.0552 | 618 |
| DP140-10-E-F1 | 27,679 | 501 | 0.9965 | 4.68 | 0.0198 | 614 |
| DP140-10-E-F2 | 27,679 | 424 | 0.9969 | 4.23 | 0.0331 | 507 |
| DP140-10-E-F3 | 27,679 | 470 | 0.9952 | 3.7 | 0.0714 | 601 |
| DP140-10-E-M1 | 27,679 | 423 | 0.9953 | 3.46 | 0.0938 | 581 |
| DP140-10-E-M2 | 27,679 | 343 | 0.9963 | 3.32 | 0.1216 | 478 |
| Total | 12,225,944 | 3,933 |  |  |  |  |
| * DP represented deep-sea sediment samples, while 3/10 indicated the mouse fecal samples collected on day 3/10. C/E represented the control group/experiment group. F/M showed female/male mice. The number indicated the repeat. | | | | | | |

| **Table S3. The significantly increased or decreased bacteria in the gut microbiota of mice treated with the viruses purified from deep-sea sediments.** | | |
| --- | --- | --- |
| **Sample** | **Increased bacteria** | **Decreased bacteria** |
| DP008 | *Bifidobacterium, Lactobacillus, Parasutterella* | *Lachnospiraceae FCS020 group, Anaerotruncus, Bacteroides, Candidatus Soleaferrea, Christensenellaceae R-7 group, Christensenellaceae_uncultured, Clostridium sensu stricto 1, Coriobacteriaceae_uncultured, Enterorhabdus, Erysipelotrichaceae_uncultured, Family XIII AD3011 group, Family XIII UCG002, Lactococcus, Ruminococcaceae UCG-010, Oscillibacter, Parabacteroides, Peptococcus, Rhodospirillaceae_uncultured, Ruminiclostridium, Ruminiclostridium 9, Ruminococcaceae UCG-005, Ruminococcaceae UCG-013, Ruminococcaceae_uncultured, [Eubacterium] brachy group, [Eubacterium] coprostanoligenes group, [Eubacterium] nodatum group* |
| DP010 | *Acetitomaculum, Anaerostipes, Enterococcus, Faecalibaculum, Ruminococcaceae UCG-010, Prevotellaceae UCG-001, Rhodospirillaceae_uncultured, Ruminococcus 1* | *Candidatus Stoquefichus, Lachnospiraceae UCG-001, Lactobacillus, [Eubacterium] nodatum group* |
| DP016 | *Lachnospiraceae NK4A136 group, Lachnospiraceae_Unclassified, Roseburia, Anaerotruncus, Enterorhabdus, Lachnoclostridium, Lachnospiraceae_uncultured, Oscillibacter, Peptococcaceae_uncultured, Ruminiclostridium 5, Ruminiclostridium 9, Ruminococcaceae UCG-005, Ruminococcaceae_uncultured* | *Bacteroidales S24-7 group_norank, Alloprevotella, Marvinbryantia, [Eubacterium] ventriosum group* |
| DP018 | *Lactobacillus, Lachnospiraceae NK4A136 group, Ruminococcaceae UCG-005, Candidatus Saccharimonas, Lachnospiraceae UCG-006, Ruminococcaceae NK4A214 group, [Eubacterium] brachy group* | *Citrobacter, Aerococcus, Klebsiella, Escherichia-Shigella, Glutamicibacter, Parabacteroides, Staphylococcus, [Eubacterium] coprostanoligenes group, Enterococcus, Solibacillus* |
| DP020 | *Enterorhabdus* | *Lachnospiraceae FCS020 group, Acetitomaculum, Roseburia, Lachnospiraceae_uncultured, [Ruminococcus] torques group, Anaerotruncus, Tyzzerella, Ruminiclostridium, Clostridiales vadinBB60 group_norank, Alistipes* |
| DP029 | *Alloprevotella, Acetitomaculum, Erysipelatoclostridium, Ruminococcaceae UCG-014, Butyricimonas, Parasutterella, Mollicutes RF9_norank, Clostridiales vadinBB60 group_norank, Ruminococcaceae UCG-010, Candidatus Soleaferrea, Streptococcus, Staphylococcus, Ruminococcus 1, Turicibacter, Tyzzerella 3* | *Christensenellaceae_uncultured, Tyzzerella, Ruminococcaceae UCG-005* |
| DP048 | *Bilophila, Clostridiales vadinBB60 group_norank, Ruminococcaceae UCG-010, Alistipes, Butyricicoccus, Ruminococcaceae UCG-009, Oscillibacter, Lachnospiraceae FCS020 group, Parabacteroides* | *Lactobacillus, Candidatus Saccharimonas* |
| DP053 | *Lachnoclostridium, Family XIII UCG-001, [Eubacterium] xylanophilum group, Ruminiclostridium, Anaerovorax, Lachnospiraceae FCS020 group, Lachnospiraceae NK4A136 group, Butyricicoccus, Marvinbryantia, Lachnospiraceae UCG-001, Anaerotruncus, Lachnospiraceae_uncultured, Ruminococcaceae UCG-005, Ruminiclostridium 6, Oscillibacter, Ruminococcaceae UCG-009* | *Ruminococcaceae UCG-010, [Eubacterium] coprostanoligenes group, Alistipes, [Ruminococcus] torques group, Ruminococcus 2* |
| DP054 | *Odoribacter, Ruminococcaceae UCG-003, Ruminiclostridium, Ruminococcaceae UCG-009, Ruminiclostridium 9, Christensenellaceae R-7 group, Roseburia, Oscillibacter, Blautia, Anaerotruncus, Coprococcus 1, Candidatus Soleaferrea, Family XIII UCG-002, Anaerovorax, Lachnospiraceae_uncultured, Peptococcaceae_uncultured, Parabacteroides, Ruminococcaceae UCG-010, Ruminococcus 1, Ruminococcaceae_uncultured, Tyzzerella, [Eubacterium] brachy group* | *Candidatus Stoquefichus, Lactobacillus* |
| DP059 |  | *Lachnospiraceae UCG-006, Ruminococcaceae UCG-009, [Eubacterium] xylanophilum group, Lachnospiraceae_uncultured, Tyzzerella, Family XIII UCG-001, Acetitomaculum, Marvinbryantia, Lachnoclostridium, Peptococcaceae_uncultured, Lachnospiraceae FCS020 group, Ruminococcaceae UCG-005, Ruminococcus 2* |
| DP061 | *Enterococcus, Coprococcus 1, Brevundimonas, Blautia, Acetitomaculum, Anaerovorax, Lachnospiraceae NK4A136 group, Family XIII UCG-001, Acetatifactor, Enterorhabdus, Hydrogenispora, Sporobacter, Lachnospiraceae_uncultured, Ruminiclostridium 9* | *Bacteroidales S24-7 group_norank, Bacteroides, Ruminococcus 2, Rikenellaceae RC9 gut group* |
| DP062 |  | *Desulfovibrio, Ruminococcaceae UCG-010, Ruminiclostridium 9, Ruminococcus 1, Ruminococcaceae UCG-014, Peptococcaceae_uncultured, Candidatus Arthromitus, Coprococcus 1, [Eubacterium] ventriosum group, Blautia, Tyzzerella 3* |
| DP064 | *Enterorhabdus, Butyricicoccus, Anaerotruncus, Family XIII UCG-001, Lachnospiraceae FCS020 group, Lachnospiraceae_Unclassified, Lachnospiraceae NK4A136 group, Family XIII UCG-002, Oscillibacter, Lachnospiraceae_uncultured, Mucispirillum, Lachnoclostridium, Lachnospiraceae UCG-006, Roseburia, Ruminiclostridium 5, Ruminiclostridium 6, Ruminiclostridium 9, Ruminococcaceae UCG-005, [Eubacterium] xylanophilum group, Ruminococcaceae_uncultured, [Eubacterium] brachy group, [Ruminococcus] torques group* | *Bacteroidales S24-7 group_norank, Bacteroides, Clostridium sensu stricto 1, Candidatus Saccharimonas, Anaerovorax, Faecalibaculum, Flavobacteriaceae_uncultured, Parabacteroides, Rhodospirillaceae_uncultured, Rikenellaceae RC9 gut group, Turicibacter, [Eubacterium] coprostanoligenes group, [Eubacterium] nodatum group* |
| DP071 |  | *Christensenellaceae R-7 group, Christensenellaceae_uncultured, Family XIII UCG-001, Erysipelotrichaceae_uncultured, Butyricicoccus, Lachnospiraceae UCG-006, Ruminococcaceae UCG-005, Lachnoclostridium, Enterorhabdus, Anaerotruncus, Ruminococcus 1, Acetitomaculum, Ruminococcaceae_uncultured, Ruminiclostridium 5, Ruminococcaceae UCG-009, [Eubacterium] nodatum group* |
| DP078 | *Coprococcus 1, Marvinbryantia, Lachnospiraceae UCG-006, Ruminiclostridium 9, Family XIII UCG-001, Roseburia, Butyricicoccus, Streptococcus, Ruminiclostridium 5, Ruminococcaceae UCG-009* | *Eggerthella, Christensenellaceae R-7 group, Peptococcaceae_uncultured, Ruminococcaceae UCG-010, Candidatus Soleaferrea* |
| DP087 | *Anaerotruncus, Oscillibacter, Lachnospiraceae FCS020 group, Butyricimonas, Lachnospiraceae NK4A136 group, Ruminiclostridium 9, Erysipelotrichaceae_uncultured, Peptococcaceae_uncultured, Clostridiales vadinBB60 group_norank, Roseburia, Tyzzerella* | *Citrobacter, Lactobacillus, Klebsiella* |
| DP088 |  | *Ruminococcaceae UCG-010, Alistipes, Peptococcaceae_uncultured, Enterococcus, Ruminococcus 2, Christensenellaceae R-7 group, Family XIII UCG-002, Ruminococcus 1, Parabacteroides, Streptococcus, Butyricimonas, [Eubacterium] coprostanoligenes group* |
| DP090 | *Allobaculum* | *Oscillibacter, Ruminococcaceae NK4A214 group, Ruminiclostridium, Lachnospiraceae UCG-006, Ruminococcaceae UCG-005, Family XIII UCG-001, Lachnospiraceae_uncultured, Ruminiclostridium 5, Roseburia, Ruminococcaceae_uncultured, Anaerotruncus* |
| DP093 |  | *Butyricimonas, Erysipelotrichaceae_Unclassified, Christensenellaceae R-7 group, Anaeroplasma, Family XIII AD3011 group, Alloprevotella, Bacteroides, Enterococcus, Candidatus Soleaferrea, Romboutsia, Acetitomaculum, Mesorhizobium, Parabacteroides, Ruminococcaceae UCG-014, Peptococcaceae_uncultured, Ruminococcus 1, Ruminococcus 2* |
| DP098 | *Lachnospiraceae NK4A136 group, Butyricicoccus, Marvinbryantia, Blautia, Candidatus Saccharimonas, Lachnoclostridium, Enterorhabdus, Roseburia* | *Enterococcus, Escherichia-Shigella, Parabacteroides, Bacteroides, Ruminococcus 1, Alistipes, Ruminococcaceae UCG-013* |
| DP105 | *Parasutterella, Enterorhabdus* | *Coprococcus 1, Family XIII UCG-001, Oscillibacter, Clostridiales vadinBB60 group_norank, Ruminiclostridium 6, Ruminiclostridium, Lachnospiraceae UCG-006, Anaerotruncus, Lachnospiraceae_uncultured, Lachnospiraceae FCS020 group, Anaerovorax, Lachnospiraceae NK4A136 group, Ruminococcus 1, Ruminiclostridium 9, Streptococcus, Tyzzerella, [Eubacterium] coprostanoligenes group, Ruminococcaceae UCG-005, Ruminococcaceae_uncultured, Ruminococcaceae UCG-009, [Eubacterium] xylanophilum group* |
| DP117 | *Lachnospiraceae UCG-001, Acinetobacter, Pandoraea, Bacteroides, Turicibacter* | *Family XIII UCG-001, Ruminiclostridium, Ruminococcaceae UCG-009, Ruminococcaceae UCG-005, Peptococcus, Anaerotruncus, Christensenellaceae_uncultured, Oscillibacter, Ruminiclostridium 9, [Eubacterium] xylanophilum group, [Eubacterium] brachy group, Ruminococcaceae_uncultured, Tyzzerella, Streptococcus, Ruminiclostridium 6, Lachnospiraceae NK4A136 group* |
| DP119 | *Ruminiclostridium 5, Ruminococcaceae UCG-009, Ruminococcaceae UCG-005, Ruminiclostridium, Anaerotruncus, Lachnospiraceae_uncultured, Ruminococcaceae NK4A214 group, Alistipes, Ruminiclostridium 9, Enterorhabdus, Lachnospiraceae NK4A136 group, Acetitomaculum, Bacteroidales S24-7 group_norank* | *Eggerthella, Christensenellaceae_uncultured, Lactobacillus* |

| Target | Primer sequence(5' to 3') |
| --- | --- |
| GAPDH-forward | GGTATCGTGGAAGGACTCATGAC |
| GAPDH-reverse | ATGCCAGTGAGCTTC CCGTTCAG |
| IL-1β-forward | GAAATGCCACCTTTTGACAGTG |
| IL-1β-reverse | CTGGATGCTCTCATCAGGACA |
| IL-10-forward | GGACAACATACTGCTAACCGAC |
| IL-10-reverse | CCTGGGGCATCACTTCTACC |
| IL-6-forward | GATGCTACCAAACTGGATATAATC |
| IL-6-reverse | GGTCCTTAGCCACTCCTTCTGTG |
| IL-17-forward | CTCTCCACCGCAATGAAGAC |
| IL-17-reverse | AGCTTTCCCTCCGCATTGA |
| IL-25-forward | CTAACCTGCTCCAGTCAGCC |
| IL-25-reverse | CACCTAATCTGGG TCGCTCC |
| TNF-α-forward | CATCTTCTCAAAATTCGAGTGACAA |
| TNF-α-reverse | TGGGAGTAGACAAGGTACAACCC |

**Table S4. Primers used for Quantitative real-time PCR**
